# A transcriptome-wide identification of ATP-binding cassette (ABC) transporters revealed participation of ABCB subfamily in abiotic stress management of *Glycyrrhiza glabra* L

**DOI:** 10.1186/s12864-024-10227-z

**Published:** 2024-03-27

**Authors:** Ritu Devi, Pooja Goyal, Bhawna Verma, Shahnawaz Hussain, Fariha Chowdhary, Palak Arora, Suphla Gupta

**Affiliations:** 1 Plant Biotechnology Division, Jammu, India; 2 CSIR-Indian Institute of Integrative Medicine, Canal Road, Jammu, 180001 India; 3 Registered from Guru Nanak Dev University, Amritsar, India; 4 Academy of Scientific and Innovative Research (AcSIR), Ghaziabad, 201002 India

**Keywords:** *Glycyrrhiza glabra*, ATP-binding cassette transporter, Nucleotide binding domain, Transmembrane binding domain, Phytohormones, Gene expression, N-1-naphthylphthalamic acid

## Abstract

Transcriptome-wide survey divulged a total of 181 ABC transporters in *G. glabra* which were phylogenetically classified into six subfamilies. Protein–Protein interactions revealed nine putative GgABCBs (-B6, -B14, -B15, -B25, -B26, -B31, -B40, -B42 &-B44) corresponding to five AtABCs orthologs (-B1, -B4, -B11, -B19, &-B21). Significant transcript accumulation of *ABCB6* (31.8 folds), -*B14* (147.5 folds), -*B15* (17 folds), *-B25* (19.7 folds), -*B26* (18.31 folds), *-B31* (61.89 folds), *-B40* (1273 folds) and -*B42* (51 folds) was observed under the influence of auxin. Auxin transport-specific inhibitor, N-1-naphthylphthalamic acid, showed its effectiveness only at higher (10 µM) concentration where it down regulated the expression of *ABCBs*, *PINs* (PIN FORMED) and *TWD1* (TWISTED DWARF 1) genes in shoot tissues, while their expression was seen to enhance in the root tissues. Further, qRT-PCR analysis under various growth conditions (*in-vitro*, field and growth chamber), and subjected to abiotic stresses revealed differential expression implicating role of *ABCBs* in stress management. Seven of the nine genes were shown to be involved in the stress physiology of the plant. *GgABCB6*, *15*, *25* and *ABCB31* were induced in multiple stresses, while *GgABCB26*, *40* & *42* were exclusively triggered under drought stress. No study pertaining to the ABC transporters from *G. glabra* is available till date. The present investigation will give an insight to auxin transportation which has been found to be associated with plant growth architecture; the knowledge will help to understand the association between auxin transportation and plant responses under the influence of various conditions.

**Supplementary Information:**

The online version contains supplementary material available at 10.1186/s12864-024-10227-z.

## Background

The ATP-binding cassette (ABC) super family is one of the largest ubiquitously present proteins known to mediate energy-driven transportation of diverse compounds in a living cell. The cargo for transportation ranges from small molecules like phytohormones to large metabolites like carbohydrates and amino acids which can be transported (import/export) independent of the trans-membrane (H^+^ electrochemical) potential difference [1]. This membrane spanning freight-carriers confer multidrug resistance, ion regulation, lipid translocation, nutrient uptake, detoxification and heavy metal tolerance directly, thereby promoting growth processes in an organism. Recent studies have highlighted their role in combating salt stress [2] and biotic and abiotic stress by maintaining homeostasis [3].

Structurally, in eukaryotes the ABC super family proteins are characterized by Trans-Membrane Domain (TMD) and Nuclear Binding Domain (NBD) which assemble to form full-sized/half-sized proteins constituting the core unit of an ABC transporter. Further classification of the super family is based on the number and orientation of the two characteristic domains and the architecture of several conserved motifs present in the NBD region of the transporter proteins [4]. A single subunit of TMD-NBD domain having forward orientation is predominantly present in ABCA to ABCD whereas the sub-family ABCG has reverse organization (NBD-TMD). The sub-families ABCE and ABCF have only two NBDs, while ABCI being most primitive possess single subunit of either NBD or accessory domain [4]. The mandatory NBD domain constitutes two major conserved sub-domains, a catalytic core domain having Walker A (GX4GK[ST]) and Walker B [(RK)X3GX3L (hydrophobic)3] boxes connected through the α-helical domain with Q loop having conserved glutamate residue, and the C loop (having the signature LSGGQ motif). The Q loop senses the γ-phosphate and attacks water in the presence of Mg^2+^; the signature sequence interacts with it to initiate ATP hydrolysis. Subsequently, the walker A of the adjacent NBD forms a pocket in association, capturing the ATP molecule inside. The conformational changes resulting in the process drives the channelization of the substrate into and out of the cell [5].Contrary to NBD, the TMD is considerably less conserved and aid in recognition and translocation of the substrate. The less conservancy in TMDs potentially invites diversity in acceptor substrate recognition. In recent years, structures of both, the importers and exporters, have been determined at high resolution and in multiple conformations that have allowed the elucidation of their molecular mechanism. However, a high degree of sequence similarity between ABC proteins could show redundancy and overlapping functionality, but does not essentially imply functional equivalence. Nevertheless, the large and functionally diverse complement of the ABC transporter super family has been categorized into eight sub-families (A-H) based on domain framework. In plants, the ABC transporters constitute both membrane bound and soluble proteins. The genome-wide sequencing, and sequence data analysis revealed presence of up to four times higher ABC transporters in the terrestrial plants compared to the non-terrestrial species [3]. The land plants have been shown to harbor more than 100 ABCs as compared to 49 ABCs in *Homo sapiens* or 79 ABCs in *E. coli*. Literature reports several studies in plants like *Brassica napus* (314), *Sorghum* (261), *Capsicum annum* (200), *Solanum lycopersicum* (154), the model plant *Arabidopsis thaliana* (130) and *Oryza sativa* (126) possessing large numbers of ABC family proteins. The most probable reason behind higher numbers and multiple copies of ABCs in plant relates to its photosynthetic capacity, metabolic diversity, absence of dedicated excretory system, adaptability, and immobile nature which requires well developed transportation system for dispersing the solute(s) across the plant tissues. In general, studies indicate participation of members of ABCB subfamily predominantly in the transport of plant hormones; ABCC subfamily in cellular detoxification by pumping toxins and heavy metals out of cells or vacuoles; and ABCG subfamily of PDR subgroup in managing abiotic stress and hormone regulation [3].

The aerial tissues of terrestrial plants are constantly exposed to various environmental and physiological stresses. Their first line of defense is the lipophilic cutin and waxes containing hydrophobic cuticle composed of secondary metabolites which are transported through ABC members and exclusively localized in the plasma membrane. ABCG12 (CER5) was the first ABC transporter identified as cuticular lipids carrier. Subsequently, several ABCGs have been reported to be involved in transportation of array of compounds required for the formation of cell wall for defense against pathogen (AtABCG40), maintaining endogenous ABA (AtABCG25), cytokinin (AtABCG14) and auxin homeostasis (AtABCB19), stomatal movement (AtABCC5), detoxification (AtABCI16) and induced by stress [3, 6–9]. Fifteen different ABC genes were identified in modulating the tolerance against salt and drought stresses from *Betula halophila* [10]. Recent publications on the transcriptome-wide computational analysis of the members of the Fabaceae family has revealed their sizeable numbers ranging between *Glycine max* with 271, *Cajanus cajan* with 159 and *Lotus japonicas* with 91 members. No reports of transcriptome-wide identification of ABC transporters are available from Glycyrrhiza species. The present study reports mining of ABC transporters from *G. glabra* species, their classification and *in-silico* characterization. Further, based on the protein–protein interaction studies, putative role of nine of the selected ABCB subfamily genes were elucidated under abiotic stress by expression analysis specifically.

## Methods

### Computational retrieval from the transcriptome resource data

For the retrieval of the ABC transporter genes from the *in-house* tissue specific transcriptome data (PRJNA664636)) of the *G. glabra* plant, the local BLAST program of BioEdit was used [11]. A Hidden Markov Model (HMM) profile of ABC transporters (PF00005) was retrieved from protein family database [12] (https://pfam.xfam.org/) and used as query with an e-value threshold of 10^–5.^A local similarity search method (tBLASTn) was performed to search the candidate ABC transporter genes with default matrix parameter against the *G. glabra* local library. The BLAST outcome identified a total of 250 contig hits. The obtained contigs were subjected to ORF finder tool (https://www.ncbi.nlm.nih.gov/orffinder/) to get full-length coding sequence of the ABC transporter genes. All the retrieved coding sequences were translated in ExPASy translate tool [13] (https://web.expasy.org/translate/) and validated by performing online BLAST (BLASTp) and conserved domain database (https://www.ncbi.nlm.nih.gov/cdd/) searches. Redundancy among the ABC transporter protein sequences was eliminated to generate a unique set of sequences for further analysis. The full-length sequence information of the mined genes was submitted to NCBI (accession numbers listed in Table 1). Further, the structural topology of GgABC proteins were determined by HMMScan tool (https://www.ebi.ac.uk/Tools/hmmer/search/hmmscan) and the pseudogenes were identified by comparing the domain architecture of each identified full-length ABCs with domain structure of *A. thaliana*. The genes were considered incomplete if they lacked required number of domains on comparing the domain architecture of each identified full-length ABCs with domain structure of *A. thaliana*. Additionally, physicochemical properties like isoelectric point (pI), molecular weight and instability index of each of the protein was determined by ExPASy ProtParam (http://web.expasy.org/protparam/) servers. The *N-glycosylation* (http://www.cbs.dtu.dk/services/NetNGlyc/) and *O-glycosylation* (http://www.cbs.dtu.dk/services/NetOGlyc-3.1/) sites were predicted and sub-cellular localization was determined by using WoLFPSORT [14] (https://wolfpsort.hgc.jp/) bioinformatics tools. Conserved motifs of each subfamily of GgABC transporter proteins were predicted using Multiple Expectation Maximization for Motif Elicitation (MEME:http://meme-suite.org/tools/meme) tool having minimum and maximum motif width 6 and 50, respectively [15]. The *TWD1* and *PIN 1* and *PIN3/5/7* genes were mined similarly from the transcriptome resource of *G. glabra* using the protein sequence of TWISTED DWARF1 (TWD1) (Q9LDC0.1), PIN1 (Q9C6B8.1) and PIN3/4/7 (Q9S7Z8.1) of *A. thaliana*.

**Table 1:** Physico-chemical parameters of ABC transporter proteins identified from *G. glabra.* The gene with accession number, Length (amino acid), Isoelectric point (pI), Molecular weight, Instability index, Subcellular localization and *N-* and *O – glycosylation*

Gene	Accession no	Protein Size (aa)	pI	Mw (kDa)	Instability Index	Subcellular localization	N-Glc	O-Glc
GgABCA1	MT379662	553	8.46	61.9	28.13	P	2	4
GgABCA2	MT379663	542	8.64	60.2	26.94	P	2	4
GgABCA3	MT379664	772	8.20	85.6	32.69	P	4	10
GgABCA4	MT379665	459	8.77	51.4	30.35	P	2	4
GgABCA5	MT379666	948	6.81	106	33.47	P	7	4
GgABCA6	MT379667	974	6.69	107	35.59	P	8	27
GgABCA7	MT379668	258	6.41	29.1	33.90	CP	0	1
GgABCA8	MT379669	236	6.33	26.5	26.78	GC	0	5
GgABCA9	MT379670	216	9.89	23.6	37.18	CY	0	3
GgABCA10	MT379671	155	9.38	17.2	40.93	N	0	1
GgABCA11	MT379672	292	8.15	32.1	32.90	N	1	0
GgABCB1	MT379673	553	9.65	60.9	39.78	P	0	2
GgABCB2	MT379674	453	8.64	49.6	37.30	P	1	0
GgABCB3	MT379675	205	5.96	23.3	36.18	GC	0	0
GgABCB4	MT379676	507	6.49	55.9	36.76	P	2	2
GgABCB5	MT379677	633	8.46	68.2	31.44	P	4	1
GgABCB6	MT379678	906	6.71	97.4	33.65	P	3	6
GgABCB7	MT379679	409	9.21	44.8	37.53	CP	0	4
GgABCB8	MT379680	694	8.15	75.7	39.08	P	2	19
GgABCB9	MT379681	465	9.19	51	34.09	P	2	3
GgABCB10	MT379682	414	8.83	45.5	31.75	CP	1	1
GgABCB11	MT379683	702	8.69	76.9	29.93	P	4	5
GgABCB12	MT379684	573	8.54	63.1	31.25	P	3	0
GgABCB13	MT379685	880	8.91	96.9	39.60	P	2	29
GgABCB14	MT379686	599	8.36	65.9	35.74	P	2	0
GgABCB15	MT379687	1179	9.05	128.9	38.31	P	2	30
GgABCB16	MT379688	403	6.12	43.4	23.29	CP	2	0
GgABCB17	MT379689	231	5.52	25.8	46.02	CY	1	0
GgABCB18	MT379690	181	5.10	19.5	55.19	EC	0	0
GgABCB19	MT379691	664	5.02	73.1	48.60	P	2	1
GgABCB20	MT379692	544	8.66	58.7	34.23	P	1	1
GgABCB21	MT379693	298	6.67	31.7	42.23	CP	1	1
GgABCB22	MT379694	573	7.64	63.8	36.43	P	5	0
GgABCB23	MT379695	496	7.18	55.5	39.80	P	5	0
GgABCB24	MT379696	314	6.25	34	42.19	CP	1	0
GgABCB25	MT379697	603	8.88	66.1	34.19	P	6	18
GgABCB26	MT379698	943	8.45	103	38.55	P	6	0
GgABCB27	MT379699	383	6.77	41.4	46.99	CP	0	2
GgABCB28	MT379700	1255	8.71	137.1	31.48	P	3	3
GgABCB29	MT379701	485	9.40	53.3	38.49	P	1	0
GgABCB30	MT379702	1396	6.04	155.4	49.67	P	7	0
GgABCB31	MT379703	168	6.42	17.8	26.69	CY	0	0
GgABCB32	MT379704	903	8.17	100.8	45.12	P	7	25
GgABCB33	MT379705	1403	6.17	155.4	47.11	P	8	8
GgABCB34	MT379706	1132	8.12	125.7	44.08	P	7	6
GgABCB35	MT379707	405	6.12	43.6	22.26	CP	2	0
GgABCB36	MT379708	348	5.59	37.6	23.37	CY	2	0
GgABCB37	MT379709	368	5.83	39.9	32.43	CY	0	1
GgABCB38	MT379710	638	5.76	70.7	50.90	P	5	27
GgABCB39	MT379711	696	9.01	76.1	32.07	P	4	8
GgABCB40	MT379712	810	8.65	88.5	36.66	V	2	12
GgABCB41	MT379713	535	8.42	58.2	34.11	V	2	7
GgABCB42	MT379714	881	9.00	96.8	40.31	P	1	28
GgABCB43	MT379715	801	6.81	87	38.30	P	3	19
GgABCB44	MT379716	802	7.57	87.1	40.41	P	2	0
GgABCB45	MT379717	786	8.21	86.5	41.80	P	4	22
GgABCC1	MT379718	1348	8.03	150	40.53	P	5	0
GgABCC2	MT379719	1483	8.18	165.1	41.73	P	5	0
GgABCC3	MT379720	1087	6.89	121.2	43.39	P	3	0
GgABCC4	MT379721	1529	7.97	170.8	32.71	P	7	5
GgABCC5	MT379722	1126	6.86	125.7	30.19	P	5	1
GgABCC6	MT379723	1502	7.67	167.7	31.71	P	7	4
GgABCC7	MT379724	846	8.04	94.3	30.33	P	3	0
GgABCC8	MT379725	1466	6.54	162.8	35.28	P	4	4
GgABCC9	MT379726	671	6.34	75.2	39.10	P	7	6
GgABCC10	MT379727	1483	8.46	166.5	37.88	P	8	9
GgABCC11	MT379728	1476	7.88	166.1	37.56	P	10	8
GgABCC12	MT379729	249	5.90	27.9	39.90	N	3	0
GgABCC13	MT379730	880	8.91	96.9	39.60	P	2	29
GgABCC14	MT379731	599	8.36	65.9	35.74	P	2	10
GgABCC15	MT379732	1179	9.05	128.9	38.31	P	2	30
GgABCC16	MT379733	403	6.12	43.4	23.29	V	2	0
GgABCC17	MT379734	231	5.52	25.8	46.02	V	1	0
GgABCC18	MT379735	181	5.10	19.5	55.19	P	0	0
GgABCC19	MT379736	664	5.02	73.1	48.60	P	2	1
GgABCC20	MT379737	544	8.66	58.7	34.23	P	1	0
GgABCC21	MT379738	302	8.16	33.3	40.68	EC	1	1
GgABCC22	MT379739	824	8.84	92.9	36.17	P	2	0
GgABCC23	MT379740	706	7.16	79.1	36.50	P	3	5
GgABCC24	MT379741	269	5.85	30.1	33.57	CP	1	0
GgABCC25	MT379742	730	5.94	81.3	42.66	P	2	5
GgABCC26	MT379743	803	6.08	89.4	40.02	P	2	4
GgABCC27	MT379744	1051	8.09	117.7	37.13	P	2	3
GgABCC28	MT379745	1348	8.79	151.1	36.23	P	6	9
GgABCC29	MT379746	823	9.10	92.4	35.10	P	1	3
GgABCC30	MT379747	1176	7.71	131.5	39.98	P	2	12
GgABCC31	MT379748	1050	8.32	117.7	39.30	P	2	12
GgABCC32	MT379749	444	7.00	49.5	49.90	P	0	0
GgABCC33	MT379750	305	5.61	33.5	42.63	P	0	0
GgABCC34	MT379751	366	4.83	40.6	31.95	CP	0	4
GgABCC35	MT379752	686	4.86	75.6	32.73	P	1	4
GgABCC36	MT379753	647	4.98	71.3	30.32	P	1	2
GgABCC37	MT379754	161	4.79	18	43.89	CP	2	0
GgABCC38	MT379755	159	5.05	18.2	38.68	CY	1	0
GgABCC39	MT379756	806	5.84	89.9	40.54	P	3	4
GgABCC40	MT379757	609	8.51	68.4	44.57	P	2	1
GgABCC41	MT379758	1326	6.86	149.1	41.89	P	2	8
GgABCC42	MT379759	687	6.20	77.3	42.57	P	2	0
GgABCC43	MT379760	680	6.52	76.5	42.57	P	2	0
GgABCC44	MT379761	337	9.40	37.3	48.13	CP	1	0
GgABCC45	MT379762	514	6.22	57.2	45.03	P	2	1
GgABCC46	MT379763	183	8.28	20.2	52.47	P	0	0
GgABCC47	MT379764	133	8.39	14.9	49.29	CY	0	0
GgABCC48	MT379765	704	9.12	79.5	35.93	P	2	0
GgABCC49	MT379766	1013	8.60	113.9	39.66	P	2	9
GgABCC50	MT379767	1431	8.01	160.8	39.52	P	2	9
GgABCC51	MT379768	1053	8.14	118.4	39.17	P	2	7
GgABCC52	MT379769	1213	7.31	136.5	40.84	P	1	7
GgABCC53	MT379770	1192	7.30	134.2	40.65	P	1	8
GgABCC54	MT379771	530	8.66	59.3	41.69	CP	2	0
GgABCF1	MT379772	396	6.88	45.8	38.54	CP	0	2
GgABCF2	MT379773	471	5.77	53.5	37.91	CY	1	2
GgABCF3	MT379774	328	8.30	36.8	40.06	CP	0	2
GgABCF4	MT379775	297	6.66	33	33.82	P	0	0
GgABCF5	MT379776	314	6.49	34.8	35.50	CP	0	0
GgABCF6	MT379777	179	7.93	19.5	39.65	N	3	12
GgABCF7	MT379778	710	5.88	80	33.81	CY	2	9
GgABCF8	MT379779	710	5.88	80	33.81	CY	2	9
GgABCF9	MT379780	699	6.33	78.5	31.45	CP	4	16
GgABCF10	MT379781	654	5.51	73.1	30.02	CP	5	13
GgABCF11	MT379782	559	5.46	62.3	27.51	CP	4	13
GgABCF12	MT379783	413	5.25	46	36.73	CP	2	5
GgABCF13	MT379784	283	8.51	32.1	28.10	M	0	2
GgABCG1	MT379785	755	8.94	83	38.02	P	5	9
GgABCG2	MT379786	776	9.31	85.7	41.17	P	8	35
GgABCG3	MT379787	759	8.92	83.8	40.08	P	1	4
GgABCG4	MT379788	638	8.83	70.9	36.32	P	1	2
GgABCG5	MT379789	1086	7.57	124.1	40.44	P	3	10
GgABCG6	MT379790	1003	7.54	114.6	41.53	P	3	1
GgABCG7	MT379791	587	8.10	66.5	37.11	P	2	4
GgABCG8	MT379792	1398	7.48	157.8	35.22	P	5	4
GgABCG9	MT379793	660	8.86	74.3	45.57	P	2	0
GgABCG10	MT379794	605	9.11	68.2	43.74	P	2	0
GgABCG11	MT379795	185	9.42	19.7	33.63	CY	1	0
GgABCG12	MT379796	365	9.36	39.2	31.08	CY	2	2
GgABCG13	MT379797	617	9.41	68.4	34.38	P	1	1
GgABCG14	MT379798	1154	9.04	130	34.20	P	3	5
GgABCG15	MT379799	673	8.62	75	37.21	P	3	5
GgABCG16	MT379800	867	8.90	98.4	37.03	P	2	3
GgABCG17	MT379801	683	9.08	75.6	41.32	P	1	2
GgABCG18	MT379802	685	9.09	76	37.20	P	1	0
GgABCG19	MT379803	282	5.48	30.4	38.62	CY	0	0
GgABCG20	MT379804	313	5.40	34.6	34.81	CY	1	3
GgABCG21	MT379805	835	9.38	92.9	42.18	P	4	5
GgABCG22	MT379806	703	8.83	78.4	32.43	P	3	21
GgABCG23	MT379807	663	8.48	74.3	39.15	P	3	0
GgABCG24	MT379808	695	7.12	77.4	41.57	P	2	0
GgABCG25	MT379809	374	6.61	40.9	45.43	N	1	2
GgABCG26	MT379810	333	6.53	36.3	46.41	N	1	1
GgABCG27	MT379811	302	7.64	32.9	48.16	N	0	1
GgABCG28	MT379812	624	9.12	69.5	42.62	P	1	3
GgABCG29	MT379813	1089	8.84	124.7	39.02	P	5	8
GgABCG30	MT379814	673	6.42	74.2	39.42	P	2	3
GgABCG31	MT379815	315	5.59	33.8	48.81	CP	1	0
GgABCG32	MT379816	635	8.09	70	40.28	P	1	1
GgABCG33	MT379817	157	6.29	16.9	34.82	CP	0	0
GgABCG34	MT379818	647	8.55	72.9	45.42	P	3	1
GgABCG35	MT379819	601	9.18	67.6	45.67	P	3	4
GgABCG36	MT379820	656	6.56	72.5	41.18	P	1	1
GgABCG37	MT379821	606	9.35	67.5	47.41	P	2	2
GgABCG38	MT379822	598	9.21	67.4	39.64	P	2	9
GgABCG39	MT379823	427	7.03	46.7	44.48	N	1	15
GgABCG40	MT379824	355	8.47	38.7	48.63	CY	1	12
GgABCG41	MT379825	536	7.20	59.7	44.45	P	1	1
GgABCG42	MT379826	296	8.91	32.3	49.62	N	2	11
GgABCG43	MT379827	317	9.68	34.9	44.46	EC	4	25
GgABCG44	MT379828	347	9.65	38.2	44.44	CP	4	30
GgABCG45	MT379829	854	5.77	96.8	39.26	P	2	13
GgABCG46	MT379830	588	8.83	65.3	34.66	P	2	3
GgABCG47	MT379831	643	5.92	72.1	32.37	P	4	4
GgABCG48	MT379832	387	7.16	43.3	37.82	CY	2	4
GgABCG49	MT379833	617	8.43	69.6	38.55	CY	2	2
GgABCG50	MT379834	113	8.62	12.2	35.54	CP	0	0
GgABCI1	MT379835	294	6.26	32.4	44.93	M	2	0
GgABCI2	MT379836	193	9.33	21.2	48.12	M	2	0
GgABCI3	MT379837	202	9.14	22	37.81	CP	1	4
GgABCI4	MT379838	270	6.44	29.6	41.15	CP	1	4
GgABCI5	MT379839	186	5.93	20	36.20	N	0	0
GgABCI6	MT379840	266	5.78	28.8	36.62	CY	3	2
GgABCI7	MT379841	229	9.99	25.9	43.80	M	0	0
GgABCI8	MT379842	312	8.65	33.8	43.08	CP	1	9

*CP* Chloroplast, *CY* Cytoplasm, *EC* Extracellular, *GC* Golgi complex, *M* Mitochondria, *N* Nucleus, *P* Plastid, *V* Vacuole

### Multiple sequence alignment and phylogenetic classification

The mined ABC super family genes were classified into various subfamilies (A-I) on the basis of conserved NBDs. The ABC transporter protein sequences of *A. thaliana* (120) retrieved from TAIR [16](http://www.Arabidopsis.org/) and *G. uralensis* (110) downloaded from the publicly available transcriptome reference source(http://ngs-data-archive.psc.riken.jp/Gur-genome/download.pl.) were used for phylogenetic assessment. Initially, the retrieved sequences from the two plant species along with *G. glabra* species mined from the *in-house* transcriptome were searched in HMMScan to get the NBD region. The conserved NBD region was aligned using CLUSTALW for the construction of phylogenetic tree using MEGA 7.0 with neighbor-joining (JTT substitution model) and partial deletion method with 1,000 bootstrap replicates [17]. The phylogenetic tree was represented using iTOL [18] (https://itol.embl.de/). The protein–protein interactions were predicted employing STRING (https://doi.org/10.1093/nar/gkw937) taking *A. thaliana* as a model plant using Markov clustering with inflation factor of 8.5 [19]. The functions were putatively assigned to ABC proteins based on the respective Arabidopsis orthologs in the protein network.

### Plant material and treatments

Two months old *in-vitro* cultured plants were grown in SPB medium published earlier [20] under controlled conditions of 25 °C (± 1.5) temperature and a 16 h light/8 h dark cycle (light intensity of 200 mmol m^–2^ s^–1^) and were used for the treatments and the expression studies. The plants were subjected to eight different abiotic stresses including phytohormones (Naphthaleneacetic acid and Methyl Jasmonate), cold, dark, UV rays, senescence, drought and carbon starvation. For the phytohormone treatments, three plants per time point (two sets of 21 plants) grown in liquid SPB medium were individually subjected to NAA (50 µM) and MeJA (100 µM) treatments for 0, 4, 8, 16, 32, 64, and 128 h. Further for abiotic stress, six sets each having three plants were independently subjected to six treatments. Dark and carbon starvation were given for 48 h, UV irradiation for 12 h, and drought and cold treatment (4 °C) for 24 h. For senescence, green and yellow leaves of the *in-vitro* grown plants were used. Further, a set of 9 plants (comprising of 3 plants each) were subjected to two concentrations of N-1-naphthylphthalamic acid (5 µM & 10 µM) treatments. The treated and untreated control plants were *in-vitro* grown for 15 days to see the effect of auxin-specific inhibitor on the expression profile of *ABCBs* and associated proteins. All the respective controls were kept under similar respective conditions. The control and treated plants were harvested at the appropriate times as indicated, frozen in liquid nitrogen and stored at -80 °C for RNA extraction. The cDNA synthesized from each of the plant sets were used for expression studies. The expression dynamics of the *ABCB* genes was performed in three biological replicates using qRT-PCR using actin as an endogenous control. The expression studies were also performed in different tissues (aerial and underground) grown under three different conditions (*In-vitro*, hardening unit and field).

### RNA isolation and Real-time expression studies

Total RNA of the plant samples harvested from the *in-vitro* aerial and underground tissues, grown under different conditions and variously treated shoot tissues along with respective controls were isolated using the Quick-RNA Plant Miniprep Kit (Zymo, USA). RNA integrity was checked on a 1% agarose gel and quantity were determined using NanoDrop 2000C spectrophotometer (Thermo Scientific, USA). Synthesis of cDNAs (from 2 µg of RNA) was carried out using Superscript VILO cDNA Synthesis Kit (Thermo Scientific, USA). qRT-PCRs were performed using the SYBR Green PCR Master Mix (Takara, Japan) for each tissue sample. Gene-specific RT primers having amplicon size between 100 to 150 bp were designed manually (Supplementary Table S2). *β-Actin* was used as internal reference and for normalization. Initially, semi-quantitative PCR was done to see the response of the nine genes in the selected eight treatments. Only genes which were found to be up regulated or highly down regulated were assessed further for real-time quantification using real-time PCR. The qRT-PCR amplification reaction was performed in a 10 μl reaction volume having 5 μl of SYBR Green PCR Master Mix, 0.2 μl of each primer (10 pm), 0.2 μl of ROX, 1 μl cDNA template (100 ng/μl) for all the selected nine genes. PCRs with no template (negative control) were also performed for each primer pair. The real-time PCR (Applied Biosystems, USA) with following conditions: 30 s at 95 °C, 3 s at 95 °C, optimized Tm 60 °C for 1 min (40 cycles) followed by 95 °C (15 s), 60 °C (30 s) and 95 °C (15 s) in MicroAmp fast reaction tubes (Applied Biosystems, USA) was used. The specificity of desired amplicons was analyzed by melt curve (55 °C to 95 °C) after 40 cycles. Three technical replicates and at least three independent biological replicates were performed for each treatment.

## Results and discussion

### Identification of ABC transporter genes in *G. glabra*

A total of 250 ABC transporter contig hits were retrieved from the *in-house* transcriptome data of *G. glabra* by performing local similarity search with HMM profile (PF00005). The obtained hits were verified manually, confirming the integrity of the conserved domains and motifs in the ABC proteins. Out of the 250 contigs, 181 sequences were full-length ABC transporter genes and the remaining 69 were found to be partial/pseudogenes [21] arising due to assembly error or mutational changes. Structural topology is considered one of the crucial features of ABC transporters. The ABCs family genes from *G glabra* plant were characterized on the basis of TMD and NBD (Supplementary Table S3). The arrangements of the two domains were found to be full (4 domains each) in 20 members, half (2 domain each) in 101 members and quarter (only NBD) in 60 GgABCs reflecting membrane bound or soluble nature, respectively. A typical four domain ABC transporter gene encodes 1200 amino acids long protein [4]. In the present study, the identified 181 full-length ABC genes encoded 113amino acids (ABCG50) to 1529 amino acids (ABCC4) long proteins. Their isoelectric points ranged between 4.8 (ABCC37) to 10.0 (ABCI7) and instability index was calculated to be between 55.2 (ABCB18 & ABCC18) to 22.3 (ABCB35). More than 62% (113 members) proteins were localized in plastids followed by 15% (27 members) in chloroplast and, merely 6% (11 members) each in nucleus and cytoplasm. Majority of the proteins (61.8%) were either *N*-or *O-glycosylated* and only 8.3% had no glycosylation as post translational modification. It was seen that *N-glycosylation* (42 proteins) was more than *O-glycosylation* (12 proteins) among the identified ABC proteins. Maximum *N-glycosylation* (10) was seen in ABCC11, while ABCG2 had maximum *O-glycosylated* (35). The detailed physiochemical parameters of GgABCs are listed in Table 1.

Out of the total mined full-length sequences (181), 121 ABCs were intrinsic transmembrane proteins having TMDs in their sequence organization belonging to four sub-families (GgABCA, GgABCB, GgABCC and GgABCG). Sixty genes were non-intrinsic soluble proteins containing one or more NBDs distributed across 5 subfamilies (GgABCA, GgABCB, GgABCC, GgABCG, GgABCF) and all the 8 members of GgABCI subfamily. The transmembrane proteins (121) were full sized and half-sized. Full-sized (with 2 domains each of TMD &NBD) proteins were found in GgABCB (5 members), GgABCC (14 members) and GgABCG (1 member) sub-families. Among them all the members of the GgABCBs & GgABCCs subfamilies exhibited a forward topological (TMD-NBD) × 2 orientation except one from GgABCG subfamily. The half-sized architecture (TMD-NBD) was primarily distributed in ABCA (6 members), ABCB (30 members), ABCC (30 members) and ABCG (35 members) subfamilies. All the half sized protein in subfamily ABCA (6 members) had forward orientation, while subfamilies ABCB, ABCC and ABCG possessed forward (TMD-NBD) × 1 orientation in 21, 28 and 6 members, respectively. The detail is presented in Supplementary Table S3.

### Phylogenetic analysis of ABC transporters in *G. glabra*

**Fig. 1:**
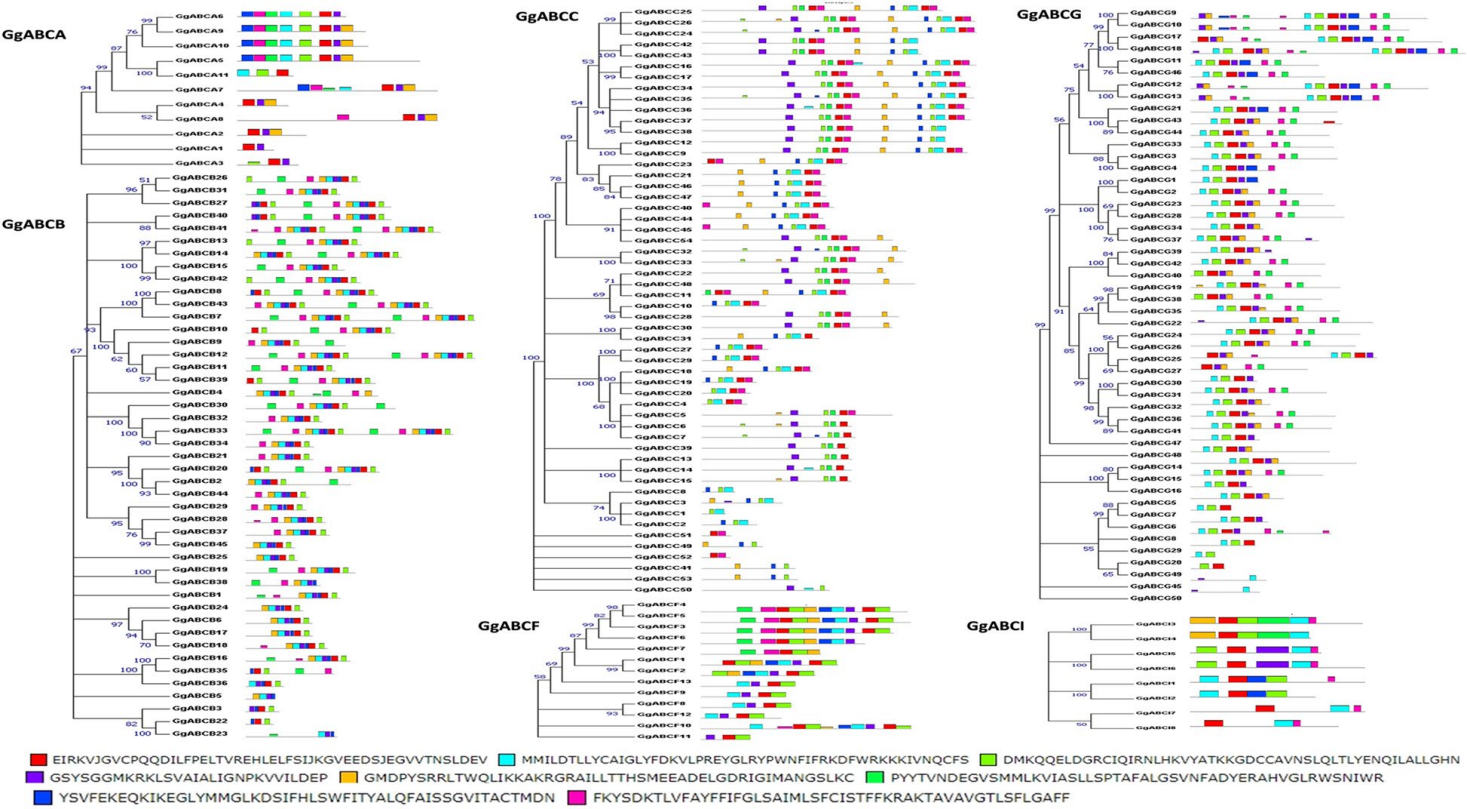
Motif visualization and classification of GgABC family proteins. Sub-families (ABCA, ABCB, ABCC, ABCF, ABCG and ABCI) were phylogenetically classified with the architecture of conserved NBD (left side). The conserved motifs (13) are represented in different color (right side). The tree was constructed using NJ method with 100 bootstrap values; conserved motifs were predicted by MEME program

The 181 GgABC protein sequences and 120 known sequences of *A. thaliana* ABC transporters were aligned and phylogenetic tree was constructed using neighbor-joining method. The tree classified 181 GgABC transporters into six sub-families (Supplementary Fig. S1). The phylogeny showed subfamily ABCC with 54 members to be the largest group, followed by 50 members of ABCG and 45 members of ABCB genes. Further, ABCF and ABCA sub-families with 13 and 11 members respectively were categorized in different groups. Eight members of ABCI subfamily, rarely found in plants, were also identified in the present study. The protein members of ABCD and ABCE sub-families of ABC transporters could not be identified in *G.glabra*. The analysis of motif sequences revealed sulphate and lipid transporter motifs in ABCA subfamily, xenobiotic motif in ABCB & ABCC, presence of ion chelate motif in ABCG, while tri galactosyl diacyl glycerol motif constituted the major part in ABCI (Fig. 1).

### ABC transporters subfamilies in *G. glabra*

**Fig. 2:**
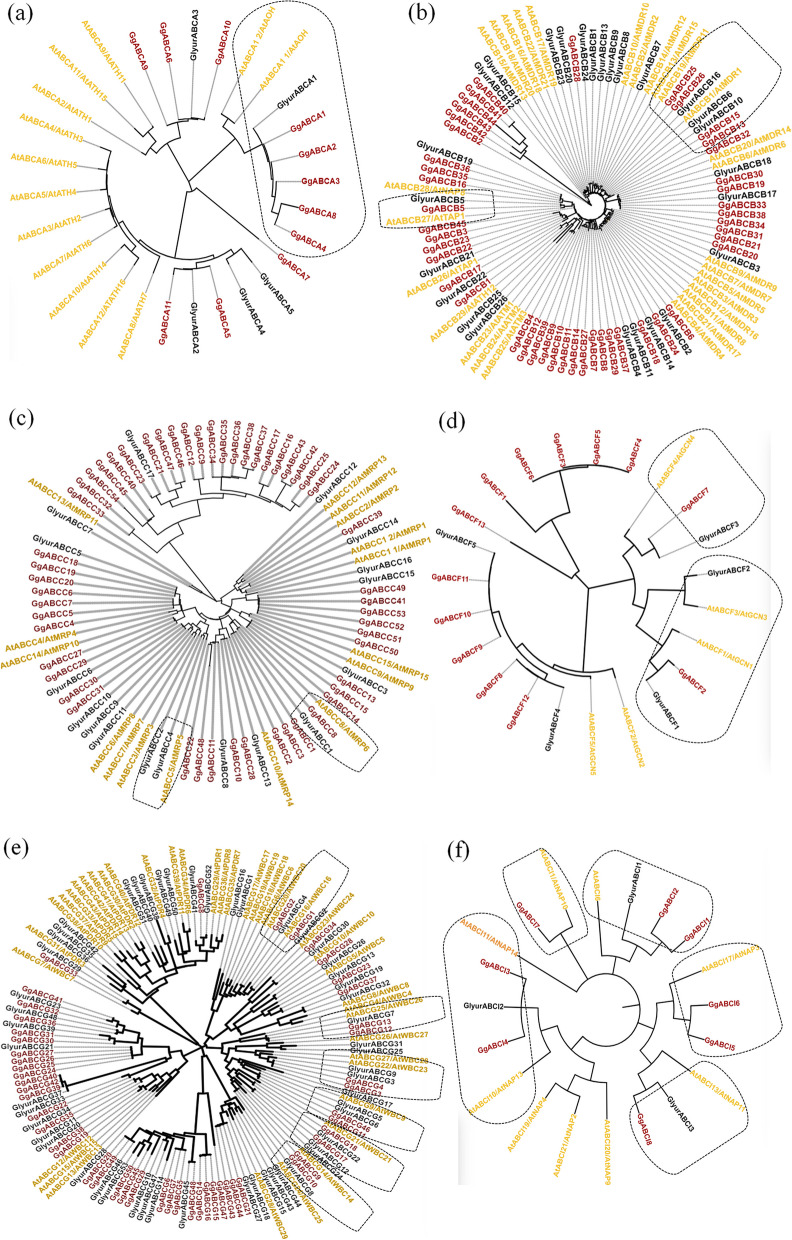
Phylogenetic tree of GgABC family. **a** ABCA subfamily comprising of ABCAs from *Glycyrrhiza glabra* (11), *Arabidopsis thaliana* (12) and *Glycyrrhiza uralensis* (5); (**b**) ABCB subfamily ABCBs of *Glycyrrhiza glabra* (45), *Arabidopsis thaliana* (29) and *Glycyrrhiza uralensis* (26); (**c**) ABCC subfamily. ABCCs of *Glycyrrhiza glabra* (54), *Arabidopsis thaliana* (15) and *Glycyrrhiza uralensis* (17); (**d**) ABCF subfamily. ABCFs of *Glycyrrhiza glabra* (13), *Arabidopsis thaliana* (5) and *Glycyrrhiza uralensis* (5); (**e**) ABCG subfamily. ABCGs of *Glycyrrhiza glabra* (50), *Arabidopsis thaliana* (41) and *Glycyrrhiza uralensis* (53); (**f**) ABCI subfamily. ABCIs of *Glycyrrhiza glabra* (8), *Arabidopsis thaliana* (9) and *Glycyrrhiza uralensis* (3). All the trees were subjected to phylogenetic analysis with 1000 bootstrap value. The tree was constructed using MEGA7 and represented by iTOL

The ABCA transporters showed the presence of both full and half sized structure; later being conserved in plants and prokaryotes [22]. In *G. glabra*, 11 members of ABCA subfamily had six half and five quarter sized proteins. All half-sized ABC transporters had forward orientation (TMD-NBD) with Rim domain of 503 aa at the N-terminal with GgABCA6 being the longest protein encoding 974 amino acids. Majority of the A subfamily proteins were *N-glycosylated* (63.6%) and localized in plastids (54.5%). Among all the 11 ABCA proteins, 4 were acidic (A5-A8) and 7 GgABCAs (A1-A4; A9-A11) were found to be basic proteins. (Table 1). All the proteins were found to be stable except GgABCA10 (40.93). The role of ABCA subfamily in animal studies has been described in intracellular trafficking, cholesterol homeostasis, lipid metabolism, pulmonary surfactant secretion and retinal transport [23] but its function in plants is not well characterized. In Arabidopsis, AtABCA1 is the only full-sized one homologue (AOH) protein implicated in seed and pollen germination [24, 25]. The half-sized AtABCAs are called ABC two homologues (ATH). In the transcriptome resource of *G.glabra*, four ATH (ABCA1-A4) proteins homologous to AtABCA1 with 5’ trimmed transmembrane domain were identified. This truncation can be an event of gene deletion during diversification or sequence alignment errors. In the phylogenetic clustering of the ABCA subfamily with *A. thaliana* and *G. uralensis,* all the four ATHs clustered with full sized AOH proteins of GlyurABCA1 and AtABCA1 (Fig. 2a). Single AOH- type protein had been reported in several dicot species such as *L.japonica* [25], *Vitis vinifera* [26], *Solanum lycopersicum* [27] and *A. thaliana* [4].

The ABCB was found to be the third largest (45 members) subfamily of ABC transporters in *G.glabra*. GgABCBs comprised of 5 full-sized, 30 half and 10 quarter sized ABC transporters. Among them all were forward-type, localized in plastid except four ABCBs (B15, B28, B30 & B33) which were true full-sized (four domains) and ABCB34 which had 5’ trimmed TMD. Among the 30 half-sized ABCBs, 21 were found to be organized in forward orientation and 9 GgABCs were (B13, B26, B38, B40-B45) in reverse orientation (Supplementary Table S3). Previous reports also demonstrated the unique topological arrangements in *Brassica rapa* [28], *Salvia miltiorrhiza* [29]*, S. lycopersicum* [27]and *Ananas comosus* [30] where they have been termed as pseudogenes. The subfamily identified proteins ranged from 168 (ABCB31) to 1403 (ABCB33) amino acids. Out of the 45 ABCBs, 18 were acidic and the rest were found to be basic proteins predominantly having *N-glycosylation*. The instability index was in between 22.26 (ABCB35) to 55.19 (ABCB18). Most of the ABC transporters (31) were stable in nature. The WoLFPSORT prediction showed that ABCBs had plastid (66.7%), chloroplast (15.6%), cytoplasmic (8.9%), nuclear (4.5%) and vacuolar (2.2%) localizations. Similarly, in Arabidopsis, the half-size ABCBs have been reported to reside in mitochondria [7], chloroplast [31] and vacuolar membrane [32]. Alignment studies showed the half size transporter GgABCB5 to be homologous to AtABCB27 with known function in Aluminum and Iron sequestration [33]. Phylogenetic classification closely clustered GgABCB13, -B15, -B25and -B26 with the characterized AtABCB1 and AtABCB19, which have shown to play crucial role in auxin transportation [34, 35] (Fig. 2b).

The 54 members of ABCC subfamily constituted the largest family of ABC identified in *G. glabra* comprising of 14 full-sized, 30 half-sized and 10 quarter-sized ABCCs. All the full-sized ABCCs followed the forward orientation, while 2 members in half-sized proteins (ABCC26 &ABCC39) had reversed arrangements. The ABCC subfamily protein length ranged between 133 (GgABCC47) to 1529 (GgABCC4) amino acids. The iso-electric point of 23 ABCCs were found to be acidic, 30 were basic and only one (ABCC32) protein was neutral in nature. The instability index varied between 23.29 (ABCC16) to 55.19 (ABCC18). Out of the 54 members, only 30 ABCCs proteins were found to be stable preponderance (88.9%) being *N-glycosylated*. Most of the members (79.6%) of the GgABCC subfamily were found to be localized in plastid except five in chloroplast (GgABCC24, -C34, -C37, -C44 & -C54), two each in vacuole (GgABCC16 &-C17), cytoplasm (GgABCC38 & -C47), one each in nucleus (GgABCC12) and in extracellular space (GgABCC21) (Table 1). Literature however cites most of the plant ABCCs are sub-cellularly localized as vacuolar proteins and a few have been reported to reside on the plasma membrane [36].The partially trimmed NBD domain (3’) was seen in the full sized ABCCs (-C1, -C28, -C52 &-C53) and half sized ABCCs (-C44, -C45, -C48 &-C54), while a single ABCC33 had 5’ trimmed TMD (supplementary Table S3). In the present study, 12 GgABCCs (-C12, -C16, -C17, -C21, -C24, -C32, -C33, -C34, -C37, -C38, -C46 & -C47) showed the presence of P-loop NTPase super family domain, while 10 GgABCCs (-C23, -C27, -C39, -C40, -C42, -C43, -C44, -C45 &-C54) had PLN03130 domain. The ABCC subfamily had membrane bound sulfonylurea receptors (SUR1 and SUR2), chloride channel cystic fibrosis trans membrane conductance regulator (CFTR) associated proteins. All the members of ABCC subfamily in *G.glabra* belonged to multi-drug resistance associated proteins involved in the transportation of glutathione-and glucuronide-conjugates, ions, toxins, and endogenous compounds [37–39].The phylogenetic tree (Fig. 2c) clustered the AtABCC8 in the same branch as GgABCC8 and GlyurABC1. Also, AtABCC5 was found clustered with GlyurABC4 and GlyurABC2 indicating their involvement in inositol hexakisphosphate transport and stomatal regulation [40, 41]. The ABCD & ABCE subfamilies were not found in *G. glabra* species.

Only 13 soluble quarter-sized fused NBDs belonging to ABCFs were identified in *G. glabra*. Notably, the proteins of this subfamily had an extended part of NBD in their sequence organization. Eight protein members (F1, F2, F3 & F8-F12) of this subfamily had an 89 amino acids long extension domain (IPR032781) in their sequence organization and majority of them were found to be localized in chloroplast only (F1, F3, F9, F10, F11 &F12). This characteristic domain is used by the proteins to stabilize the interaction mechanism on ribosome binding [42]**.** This type of arrangement was also seen in many plant species like *A. thaliana*, *G. uralensis*, *S. miltiorrhiza* and *G. max* [29, 43]. The length of the protein members in the sub-family ranged between 179 (ABCF6) to 710 (ABCF7; ABCF8) amino acids. All the GgABCFs were found to be stable except ABCF3 and majority having *N-glycosylation* (61.5%). ABCFs were also seen in cytoplasm (F2, F7 &F8), plastid (F4), nucleus (F6) and mitochondria (F13) showing involvement in diverse cellular processes. In human and yeast, ABCF proteins participate in gene regulation systems and ribosomal assembly [44]. In the phylogeny, the GgABCF2 protein was seen to be clustered with AtABCF1 & AtABCF3 with well supported sister clades together with GlyurABCF1 & GlyurABCF2. Studies have shown AtABCF1 functions together with AtABCF3 in translational regulation in response to pathogen infection [45]. AtABCF3 is also required for root growth [46] and hydrogen peroxide (H_2_O_2_) transport [47]. GgABCF7 transporter showed homology with AtABCF4 involved in protein degradation [48] suggesting the involvement of GgABCF2 & GgABCF7 in different physiological process (Fig. 2d).

ABCG sub-family represents the second largest cluster of ABC transporters in *G. glabra*. This sub-family possess both full and half sized ABC proteins, recognized as pleiotropic drug resistance (PDR) or white-brown complex (WBC) [39]. In ABCG sub-family of *G. glabra*, only one PDR containing protein (GgABCG8) was full-sized with reverse topology, 35 proteins were half-sized and 14 were quarter-sized ABC transporters. Among the half-sized transporters 29 members showed reverse orientation (NBD-TMD). Among all the 50 ABCG proteins, 10 were acidic, 39 members were found to be basic proteins and one neutral (ABCG39) in nature (Table 1). The GgABCG8 was found to be the longest protein (1398 amino acids;157KDa) and the smallest was ABCG50 (113 aa;12.2 kDa). Only 3 members of this subfamily had differential topology, the half size transporters ABCG16, ABCG48 having 3’ partial TMD and ABCG41 having 5’partial TMD. Majority of the GgABCG proteins were *N-glycosylated* (92%) and localized in plastids (66%) followed by nucleus (38.5%). Half-sized ABCGs have been reported to be localized in the plasma membrane, mitochondria, chloroplast and cytoplasm [36], while the full-size ABCGs are shown to be sub-cellularly localized as in Arabidopsis, rice and barely [49]. The length of ABCG proteins ranged between 113 (ABCG50) to 1398 (ABCG8) amino acids. Most of the proteins of this subfamily were basic in nature, except 10 ABCGs (G19, G20, G25, G26, G30, G31, G33, G36, G45 &G47). The instability index of the ABCG subfamily varied between 31.08 (ABCG12) to 49.62 (ABCG42). Half of the proteins of the subfamily was found to be stable. The proteins of ABCG subfamily have been reported to be involved in pathogen response, diffusion barrier formation and the transportation of various phytohormones, including abscisic acid (ABA), cytokinin, strigolactone and auxin derivatives in plants [50]. The half sized transporters GgABCG9 & GgABCG10 shared a sequence identity of almost 75% with AtABCG14, suggesting their involvement in lipid/sterol homoeostasis [51], vascular system development [51], long-distance cytokinin translocation [52, 53] and plant growth and immunity [54]. On the other hand, GgABCG1 and GgABCG2 were found homologous to AtABCG20 which has been demonstrated to be involved in various physiological processes such as seed dormancy [55], stomatal regulation [56] and suberin barrier formation [57] (Supplementary Table S3 and Fig. 2e).

The members of ABCI subfamily are non-intrinsic soluble proteins having single ATP binding domain [39]. ABCI members with quarter topology constitute the smallest subfamily of ABC transporters in *G. glabra.* The protein length ranged between 186 (ABCI5) to 312 (ABCI8) amino acids. Equal members of ABCI proteins were found to be acidic and basic localized in different compartments including, mitochondria (ABCI1, ABCI2 & ABCI7), chloroplast (ABCI3, ABCI4 & ABCI8), nucleus (ABCI5) and cytoplasm (ABCI6). Only three proteins of this subfamily (ABCI3, ABCI5 & ABCI6) were found to be stable at room temperature. Our study showed close proximity of I subfamily members with characterized ABCI subfamily members of *A. thaliana*. The GgABCI1 and GgABCI2 were found to be homologous to AtABCI6 which is shown to regulate the biosynthesis of Fe/S cluster [58]. Similarly, GgABCI7 was homologous to AtABCI1 reported to be involved in cytochrome-c maturation [59] and GgABCI8 showed homology with AtABCI13 implicated in lipid formation [60]. On the other hand, GgABCI5 &GgABCI6 were homologous to AtABCI17 which conferred tolerance to aluminum toxicity [61]. The GgABCI3 & GgABCI4 homologous to AtABCI10 which has been shown to be crucial for chloroplast metal transportation [62] (Fig. 2f).

### Protein–protein interaction

**Fig. 3:**
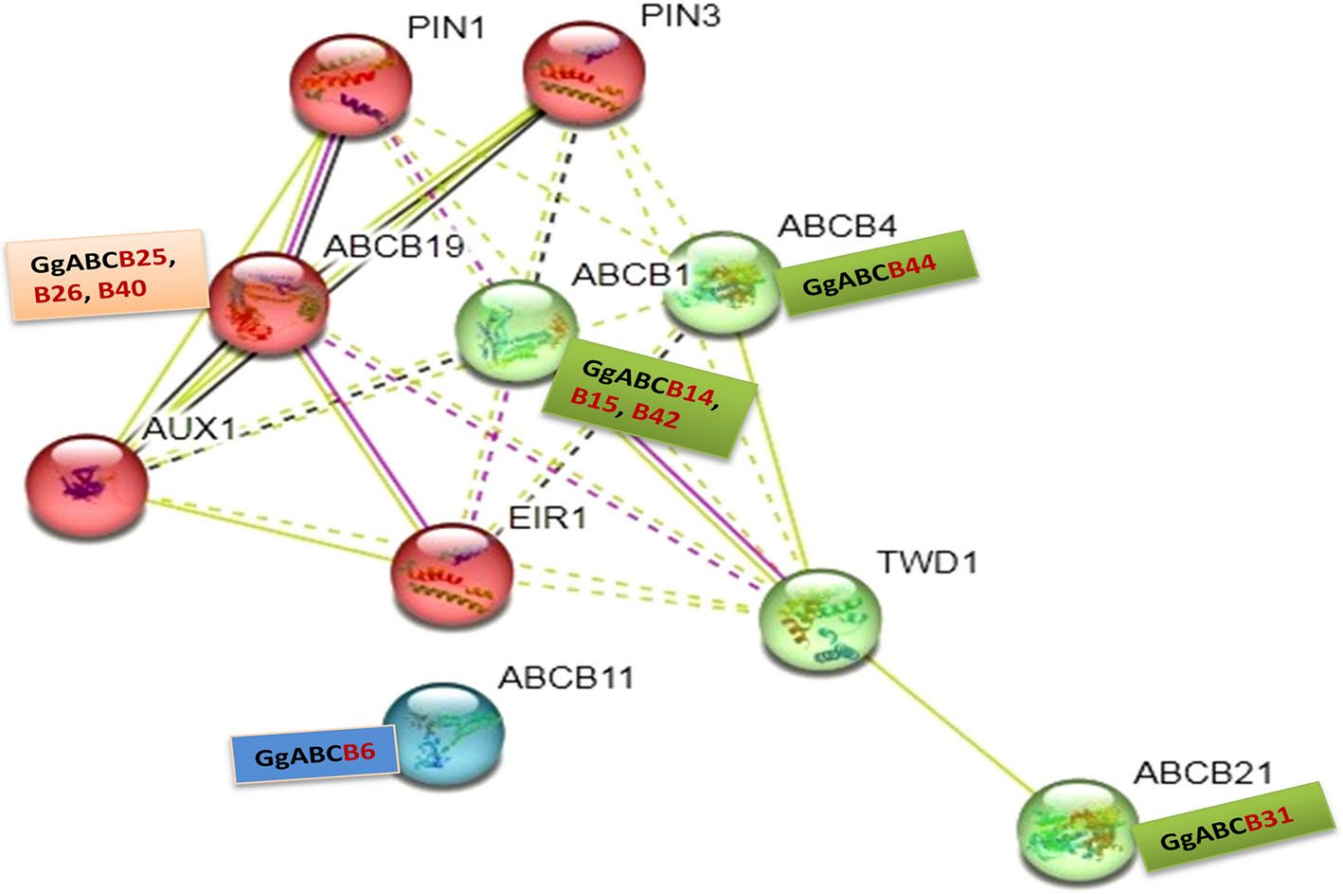
Protein–Protein interaction network of ABCB gene subfamily. Specific interactions between ABCBs and associated proteins based on Arabidopsis orthologs engaged in auxin transportation as predicted by STRING search tool. It predicted nine putative GgABCBs (-B6, -B14, -B15, -B25, -B26, -B31, -B40, -B42 &-B44) with their corresponding five AtABCs orthologs (-B1, -B4, -B11, -B19, &-B21)

The STRING pipeline predicts the interaction among taxonomically different proteins for deducing protein–protein associations. Many proteins become functionally active by interacting with another protein partner [63] jointly contributing to shared function of *cis* or *trans* in nature. The function can be inferred from curated databases or experimentally validated data available in public domains [19]. STRING based analysis can give an insight into the interacting network and a deeper insight for revealing the functional mechanism of a protein. In the current study, protein–protein interaction (PPI) network was integrated to predict the involvement of ABCB subfamily in auxin transportation with PPI enrichment *p*-value of 1.99^e−08^ [19]. The K-mean clustering was applied with clustering co-efficient of 0.645 using *A. thaliana* (taxonomic ID 3702) as a model organism. The interaction network displayed three clusters, largest being connected with five proteins ABCB19, AUX1, EIR1, PIN1 and PIN3 (red balls) of *A. thaliana* corresponding to AtABCB19 orthologs from *G. glabra* ABCB25, B26 &B40 (Fig. 3). The maximum co-expression score is observed between ABCB19 and AUX1 and PIN3 and AUX1 interacting partners (Supplementary Table S4 and Supplementary Fig. S2). Corroborating the findings, literature cites AtABCB19 protein mediating the polar transportation of auxin in association with PIN proteins [64]. The other interacting partner of the network, viz PIN1 (AT1G73590.1), PIN3 (AT1G70940.1), EIR1 (AT5G57090.1) and AUX1 (AT2G38120.1) were also reported to be involved in auxin transportation processes [65, 66]**.** The second largest cluster depicted the interaction network of four AtABC proteins (green balls) and their putative orthologs from *G. glabra*, namely AtABCB1 (GgABCB14, GgABCB15, GgABCB42), AtABCB4 (GgABCB44), AtABCB21 (GgABCB31) & TWD1. As a result, single AtABCB11 (blue ball) separated in the smallest cluster parallel to orthologs GgABCB6. The component localization of interactive partners (PIN1, PIN3, ABCB19, ABCB1, ABCB4, AUX1, EIR1, TWD1, ABCB11 and ABCB21) in protein–protein interaction network were shown in Supplementary Table S5. Based on the orthologs obtained from the intercom network, we selected and verified the involvement of nine putative GgABCBs in auxin transportation and stress biology of *G. glabra*.

### Expression dynamics of ABCB genes

#### Different growth conditions

**Fig. 4:**
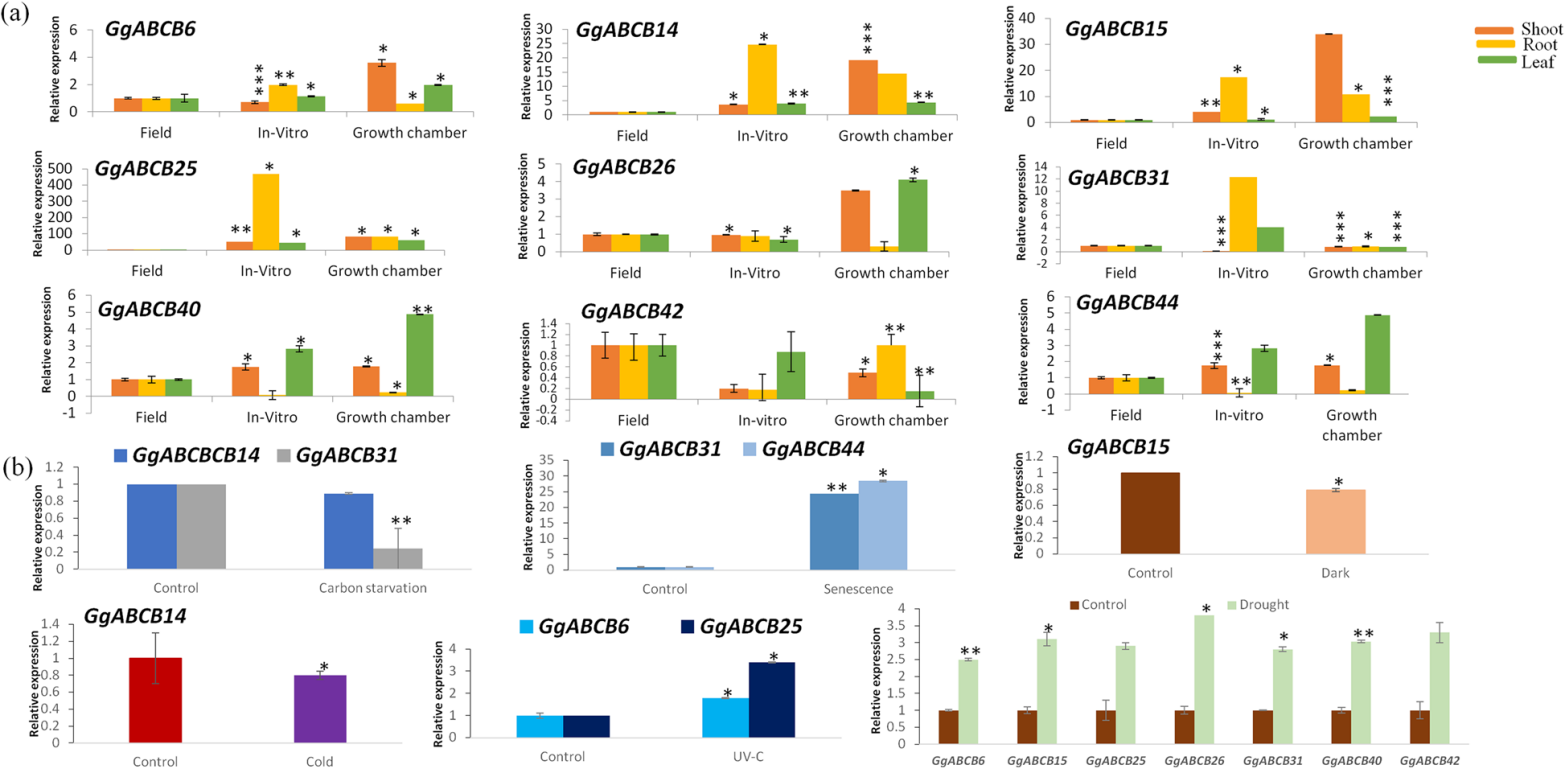
Real- time expression profiling of selected genes from ABCB subfamily of *Glycyrrhiza glabra*. The selected *GgABC* genes (**a**) under three different conditions (*in-vitro*, growth chamber and field) in tissues (shoot, root and leaf); (**b**) under six abiotic stresses; carbon starvation, senescence, cold, dark, UV-C and drought in shoot tissues. The Y-axis indicates relative expression level and X-axis indicates tissues under different conditions/stress. Three biological replicates were used to calculate error bars using standard deviation. Asterisks indicate that the corresponding gene was significantly up- or down regulated in a given treatment (*P ≤ 0.05; **P ≤ 0.01; ***P ≤ 0.001)

Gene expression pattern is a decisive factor for unraveling the function of particular genes. The differential expression profile of the selected *ABCB* subfamily genes were quantified in the aerial and underground tissues of *G. glabra* plant grown under three conditions (*in-vitro*, hardening unit and field) (Fig. 4a). RNA accumulation ranged between 0.02 to 5.9 folds in leaves, 1.5 to 8.3 folds in the shoot and 0.002 to 5.6 folds in root tissues across the nine selected ABC transporters genes. The *ABCB26* gene was found to be maximally up regulated in the leaves (5.9 folds) and shoot (3.6 folds) tissues of the plant grown in the growth chamber, while four genes-*ABCB6* (4.8 folds), *B14* (5.1 folds), *B15* (8.3 folds) and *B44* (5.5 folds) were upregulated only in the shoot tissues of the plants grown in the growth chamber as compared to the *in-vitro* grown plants. The gene *ABCB42* was unique in displaying hyper accumulation in the shoot (4.6 & 2.3 folds) and root tissues (5.6 folds each) of plants grown under field and growth chamber conditions, respectively. The data revealed the expression of selected ABC genes were more pronounced under hardening unit conditions than the plants grown under the field or *in-vitro* conditions. The probable reason for this could be the need for enhanced transportation under the glass house conditions on transferring the plants from the *in-vitro* conditions. The *in-vitro* conditions are optimal for the plant’s growth where all the required parameters are present in ample amount and no/minimum stress is experienced by the plant. Furthermore, the expression of *ABCB* transportation genes under study, were found to be more pronounced in the aerial tissues than the underground tissues in all the three conditions.

#### Abiotic stress

Recent reports have shown that the plant *ABCB* subfamily genes may be strongly inter-linked with abiotic stress [36, 67, 68]. Under nutrient starvation (N, Fe and P) and cold stress, upregulation of few *ABCB* transporter genes in *Orzya stativa*, *Zea mays* and *Citrullus lanatus* were observed [69–71]. In the current study, the *in-vitro* grown *G. glabra* plants (8 weeks old) when subjected to cold (4 °C) stress for 24 h and carbon starvation for 48 h, showed no altered expression of the selected *ABCB* genes. Hence, other abiotic stresses were explored to understand the role of *ABCB* genes in *Glycyrrhiza glabra*. The expression of the nine selected genes (*GgABCB6*, *B14*, *B15*, *B25*, *B26*, *B31*, *B40*, *B42* and *B44*) was assessed under various abiotic stress conditions like dark, UV irradiation, senescence and drought (Fig. 4b). Mannitol induced drought showed the maximum response of the *ABCB* genes. Out of the 9 genes studied, 7 genes (*ABCB6*, *B15*, *B25*, *B26*, *B31*, *B40*, *B42*) were found to be upregulated. The *ABCB26* (3.8folds) was maximally upregulated followed by *B42* (3.3 folds), and *ABCB15* & *40* each having 3 folds increase in their transcript levels. Two genes responded to senescence with *ABCB44* (28.4 folds) and *ABCB31* (24.4 folds) showing increased transcript accumulation. Under the ultra-violet radiations given for 12 h, the only *ABCB25* displayed increased (3.4 folds) transcript levels.

#### Phytohormone treatment

**Fig. 5:**
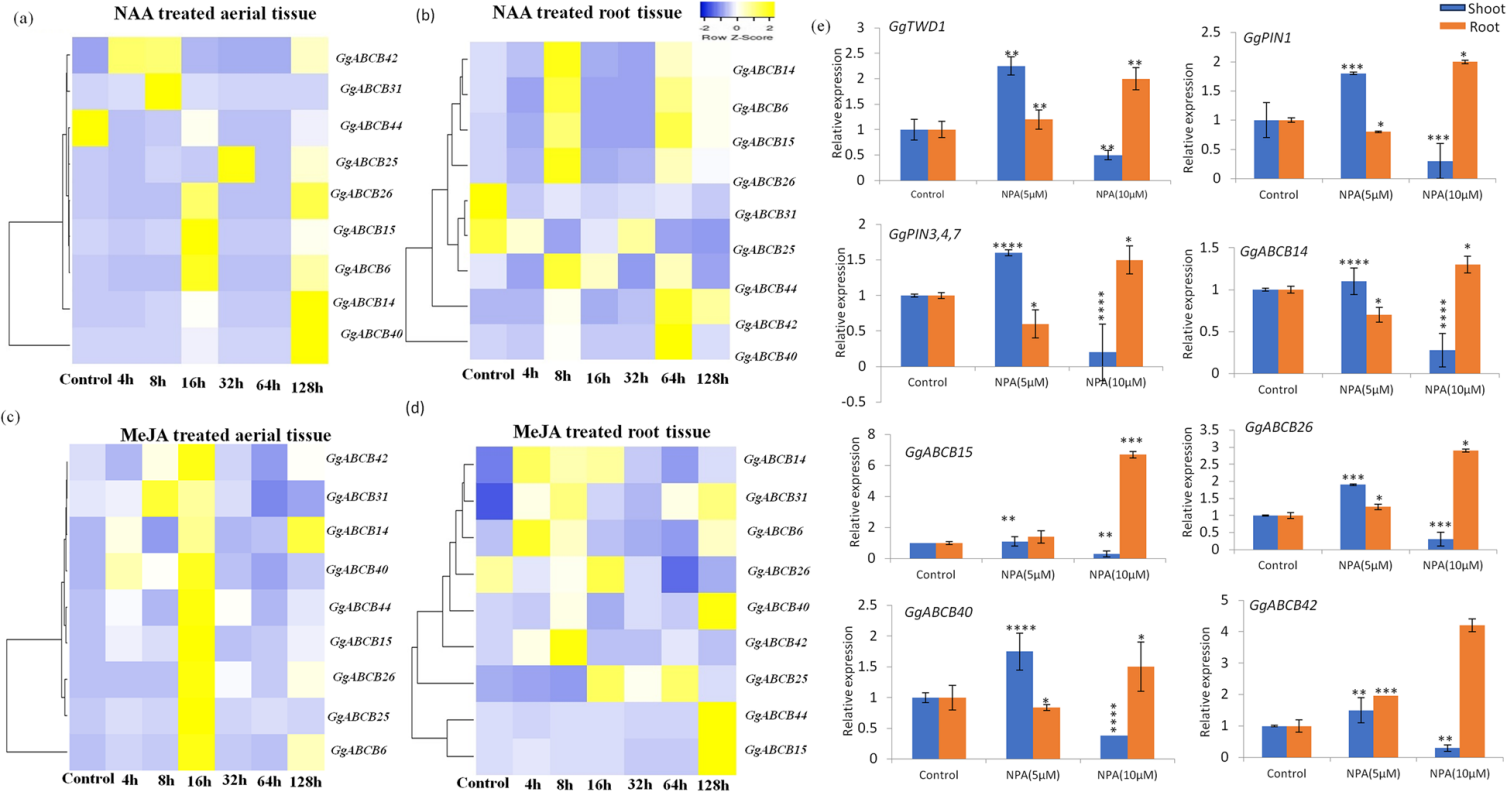
Expression analysis of selected *GgABCB* subfamily genes in shoot and root tissues under phytohormonal (**a** to **d**) and N-1-Naphthylphthalamic acid treatment (**e**). *GgABCB* genes are present in rows and treatment time duration in columns of the matrix. Heat map showing-Cluster analysis of *GgABCB* genes according to their expression profiles in Naphthaleneacetic acid (NAA) treated (**a**) shoots, (**b**) roots; and Methyl jasmonate (MeJA) treated (**c**) shoots, (**d**) roots for 0, 4, 8, 16, 32, 64 and 128 h time interval; (**e**) qRT-PCR expression (fold) represented in the form of bar graph of the *PIN1*, *PIN3/4/7*, *TWD1* (TWISTED DWARF1) and selected ABCB genes under the influence of NPA (N-1-naphthylphthalamic acid) post 15 days of treatment. *Actin* was used as an internal reference. Three biological replicates were used to calculate error bars using standard deviation. The Y-axis indicates relative expression level and X-axis indicates shoot/root tissues. Statistical significance was determined using One-way ANOVA in Graphpad prism 8.0 software. When compared with the control, single asterisk shows a significant difference with a *P*-value < 0.05. Double asterisks show a significant difference with a *P*-value < 0.01, three asterisks show a significant difference with a *P*-value < 0.001 and four asterisks shows a significant difference with a *P*-value < 0.0001

The *in-vitro* raised plants were subjected to two phytohormone treatments (Auxin and Methyl Jasmonate) for 0,4, 8, 16, 32, 64 and 128 h. The NAA treatments (50 µM) displayed maximum induction of *ABCB* genes (*ABCB14* & *40*) at 128 h. The *ABCB40* showed enhanced transcripts accumulation (1273 folds) (Fig. 5a) followed by *ABCB31* (61.9 folds), *ABCB42* (51.0 folds) and *ABCB6* (31.8 folds) at 8 h, 4 h and 16 h, respectively as compared to the untreated shoot. In the root tissues, however, the response was found to be less pronounced ranging between 0.02 to 22.6 folds. Also, the effect was observed only at two time points (8 h and 64 h). Two genes were up regulated at 8 h (*ABCB6* & *26*) and Four genes (*ABCB14*, *15*, 40 & *42*) at 64 h with *ABCB40* displaying the maximum transcripts (22.6 folds higher) in the root tissue as well when subjected to NAA treatment (Fig. 5b). The results have significance as the nine genes (*GgABCB6, GgABCB14, GgABCB15, GgABCB25, GgABCB26, GgABCB31, GgABCB40, GgABCB42* and *GgABCB44* were shown (STRING analysis) to be orthologs to *AtABCB1, AtABCB4, AtABCB11, AtABCB19* and *AtABCB21* genes which are known to be crucial players in auxin transportation. *ABCB21* functions as an importer and exporter under low and high IAA concentration respectively, thus, governing auxin concentrations in the plant cells [72]. The mutant of *AtPGP1* reduced efflux of natural and synthetic auxins [34]. Also, *ABCB19* and *ABCB4* both were found to be involved in auxin transportation wherein former facilitated seedling photomorphogenesis mediated by Photoreceptor [35]and latter regulates cellular auxin levels in the root epidermis [73]. Therefore, the inferred genes might have a vital role in the host plant in auxin transport which needs to be elucidated.

**Fig. 6:**
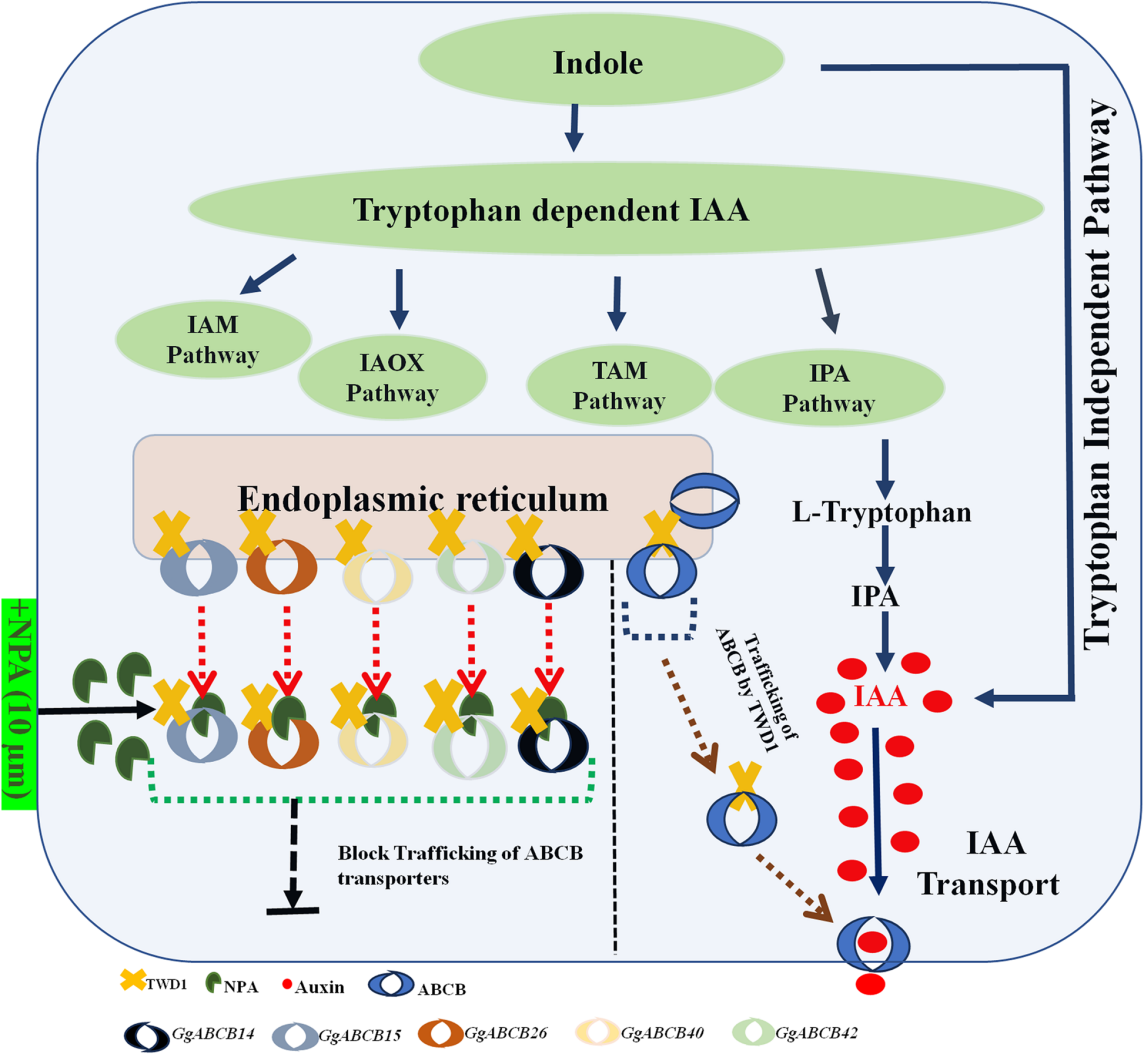
Schematic representation of IAA transportation under the influence of NPA. IAA transportation is mediated by ABCBs. A positive regulator, TWD1, PGP1-mediated auxin transporter, acts as a chaperone in association with ABCB subfamily for transportation from Endoplasmic reticulum to Plasma membrane. N-1-naphthylphthalamic acid (NPA), a polar auxin transport inhibitor, binds with high affinity to ABCBs and TWD1 complex of plant membranes [76, 77]. NPA (10 µM) disrupts TWD1-ABCB interaction resulting in inhibition of auxin transportation. Blue arrows indicate auxin biosynthesis pathways. Brown dotted arrows indicates trafficking of ABCB transporter by TWD1 from Endoplasmic reticulum to Plasma membrane, when no NPA is present. In the presence of NPA (10 µM), its binds to the ABCB –TWDI Complex and blocks the transportation

The NPA which is an auxin transport inhibitor was used to assess the effect of the selected genes in auxin transport. TWD1 which is a known positive regulator of PGP1-mediated auxin transport acts as a chaperone for transports in association with ABCB subfamily from endoplasmic reticulum to plasma membrane [74–76] N-1-naphthylphthalamic acid a non-competitive auxin efflux inhibitor [77] which blocks the basal polar auxin flow at low concentrations (< 5 mM), there by retarding the plant development. At higher concentrations ((> 5 mM), NPA seems to block polar auxin transport by affecting the trafficking components TWD1 which is an identified NPA target [76]. Further, to confirm and enhance the understanding about the participation of *ABCB* genes in auxin transport, studies using polar auxin transport inhibitors NPA was performed [67]. The *in-vitro* grown *G. glabra* plants were treated with two concentrations (5 µM &10 µM) of NPA to see their influence on the expression of *ABCBs* and *TWD1* gene from *G. glabra*. The expression studies revealed, in the shoot system, the NPA (10 µM) downregulated the expression of all the selected *ABCB* genes, namely, *ABCB14* (0.2 folds), *ABCB15*, *ABCB26*, *ABCB40* and *ABCB42* (0.3 folds) along with associated proteins-*TWD1* (0.4 folds), *PIN1* (0.3 folds) and *PIN3/4/7* (0.2 folds). However, in the root system the same concentration (10 µM) upregulated the expression (Fig. 5e) of *ABCB14* (1.3 folds), *ABCB15* (6.7 folds), *ABCB26* (2.9 folds), *ABCB40* (1.5 folds), *ABCB42* (4.2 folds) and associated proteins-*TWD1* (2 folds), *PIN1* (2 folds) and *PIN3/4/7* (1.5 folds). The expression data revealed the shoot system treated with higher concentration of NPA (10 µM) down regulated expression of all the selected *ABCB* genes and the auxin chaperon *GgTWD1* gene. However, in the root system the same concentration upregulated their expression. No effect was seen in the root and shoot tissues when subjected to lower (5 µM) concentration of NPA (Fig. 6). Shen and co-workers evaluated the effect of phytohormone stress on *Sorghum bicolour* showing few *PGP/ABCB* genes were upregulated by the treatment with polar auxin transport inhibitors (TIBA, NPA and NOA) especially in roots. They concluded that transcription of auxin transporter genes was controlled by the auxin transport inhibitors [67]. In another study, it was confirmed that the auxin is transported from the shoot to the root system controlling lateral root development. Particularly, the local application of NPA at the root-shoot junction decreased the number and density of lateral roots and reduced indole acetic acid (IAA) levels demonstrating its varied effects on the shoot and root tissues [78].

Methyl jasmonate treatment enhanced the expression of all the nine genes with 7 genes (*ABCB6*, *15*, *25*, *26*, *40*, *42* & *44*) displayed maximum transcript accumulation at 16 h of treatment (Fig. 5c). Under the influence of MeJA, *ABCB6* gene showed the maximum upregulation (258 folds) followed by *ABCB25* (32 folds), *ABCB26* (29.2folds), *ABCB15* (16.1folds) and *ABCB40* (14.1folds) in the shoot tissues at 16 h. In the root tissues *ABCB44* (20.2 folds) and *ABCB15* (13.5 folds) showed maximum induction at 128 h, while *ABCB6* (4.2), *ABCB14* (3.7) & *ABCB31* (3.1) were least induced by the treatment (Fig. 5d). Literature reports participation of *ABCB* subfamily genes under the influence of Methyl jasmonate in *Hevea Brasiliensis* [79] and *Betula halophila* [10] plants. One recent report has identified *AtABCG16* gene in the transport of jasmonic acid at the plasma membrane [1]. The physiological functions attributed to ABCB subfamily was predominantly in auxin/iron-sulfur cluster/ precursor transportation [80], accumulation of defense molecule [81], regulation of stomatal aperture and detoxification [33, 75]**.** Studies on the collaboration of ABCB subfamily in combating plant stress are sparse. The current study identified seven ABCB family members (*GgABCB6*, *15*, *25*, *26*, *31*, *40* & *42*) which were involved in manitol induced drought. They were also found to be preferentially expressed in the aerial tissues of the plant. Notably, the *GgABCB15*, *26* (3.8folds), *40* & *42* (3 folds each) were significantly upregulated in shoot tissues exclusively under drought stress. Five of the selected genes were seen to be induced by multiple stresses. For example, *GgABCB6*, *14*, *15* & *25* were upregulated under ultra-violet radiation and drought conditions, while the expression of *GgABCB31* was enhanced under senescence (24.4 folds) as well. The association of maximum number of *ABCB* subfamily genes in drought stress is probably due to their involvement in multiple physiological processes encompassing stomatal regulation and solute transportation during the insufficiency of water to the plant [82]. This interesting expression pattern may indicate that the selected *ABCB* transporter genes could dynamically behave as positive/negative regulators in different abiotic stress conditions.

## Conclusion

Overall, the present study reports mining of 181 members of ABC transporters and categorization into Six sub-families -ABCA, ABCB, ABCC, ABCF, ABCG and ABCI. The subfamily ABCI, rarely reported in plants, could be identified in *G. glabra,* however, ABCD & ABCE could not be identified from the available resource. Further, the ABCB gene family members present in the aerial tissues (leaf & stem) of the plant responded preferentially and variously to eight abiotic stresses. Five of the nine genes (*ABCB14*, *15*, *25*, *26* & *40*) studied responded to NAA treatment with *ABCB40* having the maximum transcripts accumulation in shoot (1273 folds) and root (22.6) both. Seven of the nine genes were shown to be involved in the stress physiology of the plant. *GgABCB6*, *14*, *15*, *25* and *ABCB31* were induced in multiple stresses, while *GgABCB26*, *40* & *42* were exclusively triggered under drought stress indicating their crucial role in plant adaptation and survival. The study proposes to further explore the involvement *in-depth* of the ABC family members in order to understand molecular mechanism of stress encountered by the sessile plant.

### Supplementary Information

**Additional file 1: Supplementary Figure S1.** Neighbour-Joining (JTT model) of phylogenetic tree comprising of 181 Glycyrrhiza glabra (dark green) and 120 Arabidopsis thaliana (pink) ABC transporter proteins. **Supplementary Figure S2.** Graphical representation of co-expression between interactive partners. **Supplementary Figure S3:** Spectral Analysis of RNA samples of shoot and tissues for library preparation. **Supplementary Table S1.** Protein sequence information of the identified ABC family proteins from Glycyrrhiza glabra. **Supplementary Table S2.** Primer sequences (forward/reverse) of the Real-time expression studies for the selected GgABCBs, GgPIN1, GgPIN3/4/7, GgTWD1 and Actin genes investigated in the present study. **Supplementary Table S3.** Topological inventory of ABC transporters identified from Glycyrrhiza glabra with their AtABC homologs identified in Arabidopsis thaliana. * indicates the trimmed 5’ or 3’ domain. Genes were identified by comparing with the domain architecture of Arabidopsis thaliana homolgous gene sequences [83–107]. **Supplementary Table S4:** Co-expression score between the interactive partners. **Supplementary Table S5:** Component localization of interactive partners in protein-protein interaction network. **Supplementary Table S6:** The quality control summary of RNA isolated for library preparation.

## Data Availability

The datasets generated and analyzed during the current study are available from the corresponding author on reasonable request.
