# Supplementary material for: A transcriptome-wide identification of ATP-binding cassette (ABC) transporters revealed participation of ABCB subfamily in abiotic stress management of Glycyrrhiza glabra L
Source: BMC Genomics. 2024 Mar 27;25:315. doi: 10.1186/s12864-024-10227-z (PMC10967134; doi:10.1186/s12864-024-10227-z)
Supplement: Supplementary file 1 — Additional file 1: Supplementary Figure S1. Neighbour-Joining (JTT model) of phylogenetic tree comprising of 181 Glycyrrhiza glabra (dark green) and 120 Arabidopsis thaliana (pink) ABC transporter proteins. Supplementary Figure S2. Graphical representation of co-expression between interactive partners. Supplementary Figure S3: Spectral Analysis of RNA samples of shoot and tissues for library preparation. Supplementary Table S1. Protein sequence information of the identified ABC family proteins from Glycyrrhiza glabra. Supplementary Table S2. Primer sequences (forward/reverse) of the Real-time expression studies for the selected GgABCBs, GgPIN1, GgPIN3/4/7, GgTWD1 and Actin genes investigated in the present study. Supplementary Table S3. Topological inventory of ABC transporters identified from Glycyrrhiza glabra with their AtABC homologs identified in Arabidopsis thaliana. * indicates the trimmed 5’ or 3’ domain. Genes were identified by comparing with the domain architecture of Arabidopsis thaliana homolgous gene sequences [83–107]. Supplementary Table S4: Co-expression score between the interactive partners. Supplementary Table S5: Component localization of interactive partners in protein-protein interaction network. Supplementary Table S6: The quality control summary of RNA isolated for library preparation. [file 12864_2024_10227_MOESM1_ESM.pdf]

A Transcriptome-wide identification of ATP-binding cassette (ABC) transporters revealed participation of ABCB subfamily in abiotic stress management of *Glycyrrhiza glabra* L.

Ritu Devi[1,2,4], Pooja Goyal[12,3], Bhawna Verma[1,2,4], Shahnawaz Hussain[1,2,4], Fariha Chowdhary[1,2,4], Palak Arora[1,2], Suphla Gupta[1,2,4*].

[1]Plant Biotechnology Division

[2]CSIR-Indian Institute of Integrative Medicine, Canal Road, Jammu -180001, India

[3]Registered from Guru Nanak Dev University, India

[4]Academy of Scientific and Innovative Research (AcSIR), Ghaziabad 201002, India

*Corresponding author

E-mail address: suphlabg@gmail.com;sgupta@iiim.res.in.

Orcid -ID. 0000-0001-6688-5575

Running Head: ABCBs in auxin transport and stress biology of *Glycyrrhiza*

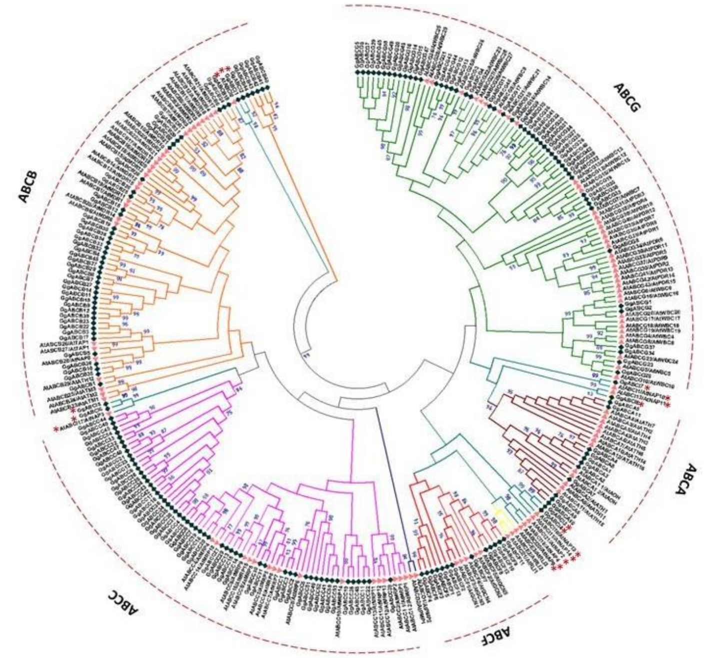

Supplementary Figure S1. Neighbour-Joining (JTT model) of phylogenetic tree comprising of 181 *Glycyrrhiza glabra* (dark green) and 120 *Arabidopsis thaliana* (pink) ABC transporter proteins. The N-terminal Nucleotide Binding Domain (NBD) was used for this phylogenetic tree construction. All the NBDs were aligned using CLUSTALW and the phylogenetic tree was constructed using MEGA7. The tree clustered into six subgroups (A to C, F, G &I). The cluster spikes are coded with different colors representing different subfamilies (ABCA; brown, ABCB; orange, ABCC; pink, ABCG; green and ABCF; red). The sequence members of the sub-family ABCI (cyan blue) (*) are dispersed in different clusters with other subfamilies.

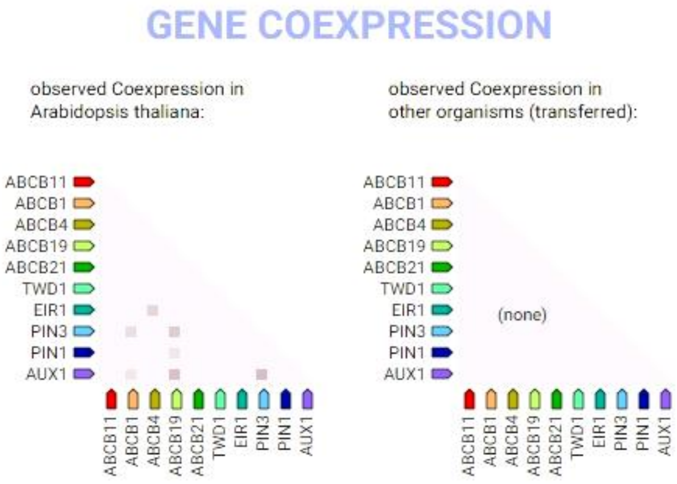

Supplementary Figure S2. Graphical representation of co-expression between interactive partners.

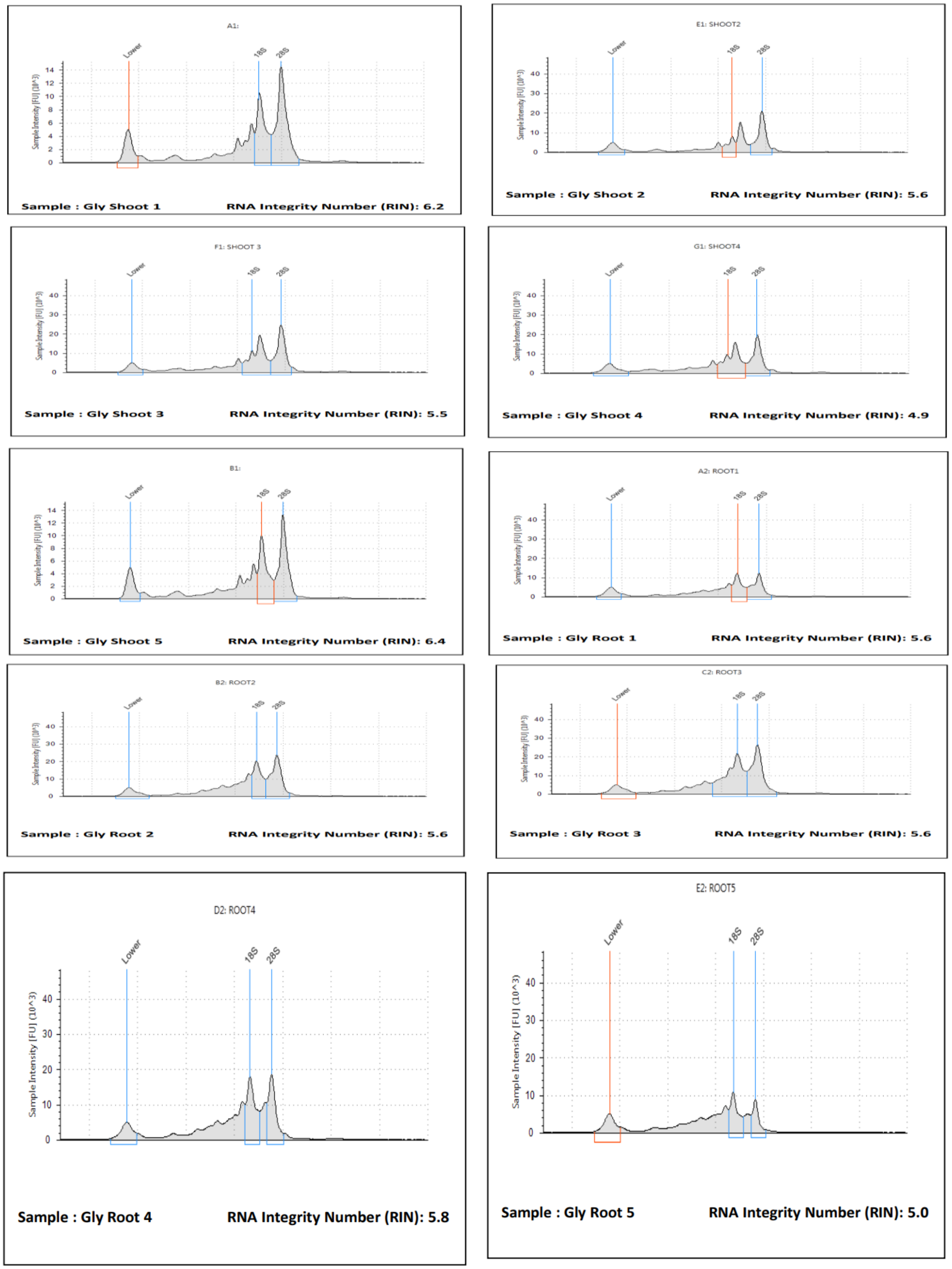

Supplementary Figure S3: Spectral Analysis of RNA samples of shoot and tissues for library preparation.

Supplementary Table S1. Protein sequence information of the identified ABC family proteins from G*lycyrrhiza glabra.*

S. No.	GgABCs	Accession no.	Protein sequence
1.	GgABCA1	MT379662	MGILYLLGFLYPISRLISYSVFEKEQKIKEGLYMMGLKDSIFHLSWFITYALQFAISSGVITACTMDNLFKYSDK<br>TLVFAYFFIFGLSAIMLSFCISTFFKRAKTAVAVGTLSFLGAFFPYYTVNDEGVSMMLKVIASLLSPTAFALGSV<br>NFADYERAHVGLRWSNIWRESSGVNFSACLLMMILDTLLYCAIGLYFDKVLPREYGLRYPWNFIFRKDFWRK<br>KKIVNQCFSNFKVKIAGKNSESEGNLLGEDTSKPAIEAISLDMKQQELDGRCIQIRNLHKVYATKKGDCCAVN<br>SLQLTLYENQILALLGHNGAGKSTTISMLVGLLPPTSGDALVFGKNIVSDIDEIRKVLGVCPQQDILFPELTVRE<br>HLELFSILKGVEEDSLEGVVTNMADEVGLADKINSVVRSLSGGMKRKLSLGIALIGNSKVIILDEPTSGMDPYS<br>KRLTWQLIKKIKKGRIILLTTHSMDEADELGDRIAIMANGSLKCCGSSLFLKHQYGVGYTLTLVKVCSFPEHA<br>VFNSYSCCFIIEKLFFLFEFPFTLVLSVSSYCFYSW
2.	GgABCA2	MT379663	MGILYLLGFLYPISRLISYSVFEKEQKIKEGLYMMGLKDSIFHLSWFITYALQFAISSGVITACTMDNLFKYSDK<br>TLVFAYFFIFGLSAIMLSFCISTFFKRAKTAVAVGTLSFLGAFFPYYTVNDEGVSMMLKVIASLLSPTAFALGSV<br>NFADYERAHVGLRWSNIWRESSGVNFSACLLMMILDTLLYCAIGLYFDKVLPREYGLRYPWNFIFRKDFWRK<br>KKIVNQCFSNFKVKIAGKNSESEGNLLGEDTSKPAIEAISLDMKQQELDGRCIQIRNLHKVYATKKGDCCAVN<br>SLQLTLYENQILALLGHNGAGKSTTISMLVGLLPPTSGDALVFGKNIVSDIDEIRKVLGVCPQQDILFPELTVRE<br>HLELFSILKGVEEDSLEGVVTNMADEVGLADKINSVVRSLSGGMKRKLSLGIALIGNSKVIILDEPTSGMDPYS<br>KRLTWQLIKKIKKGRIILLTTHSMDEADELGDRIAIMANGSLKCCGSSLFLKHQYGVGYTLTLVKSAPTASIAG<br>DIVYRHVPSATCVSEVFFSQNSSF
3.	GgABCA3	MT379664	MGILYLLGFLYPISRLISYSVFEKEQKIKEGLYMMGLKDSIFHLSWFITYALQFAISSGVITACTMDNLFKYSDK<br>TLVFAYFFIFGLSAIMLSFCISTFFKRAKTAVAVGTLSFLGAFFPYYTVNDEGVSMMLKVIASLLSPTAFALGSV<br>NFADYERAHVGLRWSNIWRESSGVNFSACLLMMILDTLLYCAIGLYFDKVLPREYGLRYPWNFIFRKDFWRK<br>KKIVNQCFSNFKVKIAGKNSESEGNLLGEDTSKPAIEAISLDMKQQELDGRCIQIRNLHKVYATKKGDCCAVN<br>SLQLTLYENQILALLGHNGAGKSTTISMLVGLLPPTSGDALVFGKNIVSDIDEIRKVLGVCPQQDILFPELTVRE<br>HLELFSILKGVEEDSLEGVVTNMADEVGLADKINSVVRSLSGGMKRKLSLGIALIGNSKVIILDEPTSGMDPYS<br>KRLTWQLIKKIKKGRIILLTTHSMDEADELGDRIAIMANGSLKCCGSSLFLKHQYGVGYTLTLVKSAPTASIAG<br>DIVYRHVPSATCVSEVGTEISFRLPMASSSAFERMFREIEGCMEKPVLSMEISGSGDKDSRGIESYGISVTTLEE<br>VFLRVAGCDYDEVECFKENNDSLISDSVASITLNDHPSTKMGYLEVFGNYKKILGFMSTMVGRACDLIFATVI<br>SFINFMGRQCCSCCLITRSTFWQHSKALFIKRAISARRDHKTIIFQLIIPAVFLFIGLLFLELKPHPDQKSLTLSTSY<br>FNPLLSGWGGGGPIPFNLSSPIAEKVCC
4.	GgABCA4	MT379665	MGILYLLGFLYPISRLISYSVFEKEQKIKEGLYMMGLKDSIFHLSWFITYALQFAISSGVITACTMDNLFKYSDK<br>TLVFAYFFIFGLSAIMLSFCISTFFKRAKTAVAVGTLSFLGAFFPYYTVNDEGVSMMLKVIASLLSPTAFALGSV<br>NFADYERAHVGLRWSNIWRESSGVNFSACLLMMILDTLLYCAIGLYFDKVLPREYGLRYPWNFIFRKDFWRK<br>KKIVNQCFSNFKVKIAGKNSESEGNLLGEDTSKPAIEAISLDMKQQELDGRCIQIRNLHKVYATKKGDCCAVN<br>SLQLTLYENQILALLGHNGAGKSTTISMLVGLLPPTSGDALVFGKNIVSDIDEIRKVLGVCPQQDILFPELTVRE<br>HLELFSILKGVEEDSLEGVVTNMADEVGLADKINSVVRSLSGGMKRKLSLGIALIGNSKVGHIFTAHFYFHILN<br>RTGIFWTYLAGYNSR
5.	GgABCA5	MT379666	MGDENTPNEPASFWTQANALLRKNLTFQKRNVKTNVKLILFPFVLCILLSLMQRLIDNQFDKAKYKCGCICTN<br>TQGDQCLEKACGIQYSDFDQVGACPIANPSEWPPLLQVPAPEYRAARTDFLPSDFPNPSCRTNGSCPVTLLFTG<br>NNQSLGQILSGNMIPSTFSINNSDIMDSLATNVLGSETETEYTNFLEPAFTSDLPIYYLQSQCTQNSTFSISVPIST<br>ASREQEVRCAQGLRLWRSSSSEVNNELYKGYRKGNTERQINEIAAGYDFLNANENIFNVSIWYNSTYRNDTG<br>FDSIALARIPRSVNLVSNAYLQFLLGPGTKMLFEFVKEMPKPETPTKLDLASLLGGLFFTWVILQLLPVVLTSL<br>VYEKQHKLRIMMKMHGLGDGPYWMISYGYFLAISILYMLCFVIFGSVIGLKFFTMNDYSIQFVFYFIYINLQIS<br>LAFLLASLFSNVKTATVIAYIGVFGTGLLAGFLFQFFVQDTSFPRGWIIVMELYPGFALYRGLYEFSQSSFSGDA<br>LGTHGMRWGDLSDSTNGMKEVLVIIFVEWLLVLFLAYYIDQVLSSGSWKSPPFFLKGFQKKPTSSFRKPSIQR<br>QGSKVFVMMEKPDVNQEREKVEQLLLEPTIDHAIVCDNLIKVYPGRDGNPEKFAVRGLSLALPQGECFGMLG<br>PNGAGKTSFINMMIGLTKPTSGTAFVQGLDIRTNMDGIYTSMGVCPQHDLLWESLTGREHLLFYGRLKNLKG<br>SALKQAVEESLKSVNLFHGGVADKQAGKYSGGMKRRLSVAISLIGDPRVVYMDEPSTGLDPASRKCLWNVV<br>KLAKRDRAIILTTHSMEEAEVLCDRLGIFVDGSLQCIGNPKELKGRYGGTYVFTMTTSMDHEEDVENLVQQL<br>SPNANKIYHISGTQKFELPKDEVRIANVFRAVETAKRNFTVSAWGLADTTLEDVFVKVARGAQAFDTLS
6.	GgABCA6	MT379667	MGLYNFKMMSSSTRGGLVLALQQYRALLKKNALLSWRGKRATMLQFLSPLIFIFLIFAVDKAIKAQTSTTSAY<br>KSVTDPPSIPSPPIPPCEDKFFVKLPCYDFVWSGDQSPKFQTIVGRIMDNNPGRPIPASKVKSFGDKAQVDTWLF<br>SNPMHCPAALHFAQKNDTVISYGIQTNSTSVAKRGKYEDPTLAFQLPLQLAAEREIARYLIGDPSFSWNVFLK<br>EFPHPGVSPISAVSTIGPTFFFAIAMFNFVLQISSLVTEKELKLRQAMNVMGLYDCAYWLSWLTWEAVVTLLS<br>SLLIVLFGMMFQFRFFLDNSFAVLFFLFFLFELNMTGLAFMLSAFISKSSSATTVGFSIFIVGFVTQLVSGRLYDR<br>SISSTFRNIWSLFPPNPFSQALTVLSDAVSTPEDHGVSWSKRGKCAVNDDDCVITINDIYMWLLATFFLWFVLA<br>IYFDNIIPNAMGVRKSILYFLNPSYWTGKGGQKVKEGGVCSCFGSAQHEEHDRPDDEDVLEEENAVKQQLTQ<br>GLVDANVSVQIHGLVKTYPGAFNISCCCKCKRSTPYHAVKGLWVNFAKDQLFCLLGPNGAGKTTAINCLTGI<br>TPVTDGDALIYGHSIQSSNGMSNIRKLIGVCPQFDILWDALSGQEHLQLFASIKGLPPASIKSITQTSLAEVRLM<br>DAAKVRAGSYSGGMKRRLSVAIALIGNPKLVILDEPTTGMDPITRRHVWDIIENAKRGRAIVLTTHSMEEADI<br>LSDRIGIMAKGRLRCIGTSIRLKSRFGTGFVANISFYGNNVEHGPANGDTISTEHREAVKQFFKNHLDVEPKEE<br>NNNFITFVIPHDREALLTNFFSELQDREEEFGISDIQLGLTTLEEVFLNIARQAELESAAAEGSLVTLTLTSGESV<br>QIPVGARFVGIPGTESAENPTGFMVEVYWEQDDTGTLCVSGHSQKVPIPQSVQLSSSPSARHQRNVSRSGSVH<br>GFVIDPSQVSSISFQ
7.	GgABCA7	MT379668	MIKKWWGKINIFPHNTTYLEPLLESSPETVVMDLDEDVDVKTERNRVLSGSIDNAIIYLHNLRKVYSEEKNHR<br>KKVAVDSLTFSVQEGECFGFLGTNGAGKTTTISMLCGEESPSDGTAFIFGKDICSHPKAAHKYIGYCPQFDALL<br>EYLTVQEHLELYARIKGVPDYRIDNVVMEKLVEFDLLKHANKPSFSLSGGNKRKLSVAIAMIGDPPIVILDEPS<br>TGSYSQSSYFFKHHENAFVHFNFYGCKKHSWALVLKS

8.	GgABCA8	MT379669	MMILDTLLYCAIGLYFDKVLPREYGLRYPWNFIFRKDFWRKKKIVNQCFSNFKVKIAGKNSESEGNLLGEDTSKPAIEAISLDMKQQELDGRCIQIRNLHKVYATKKGDCCAVNSLQLTLYENQILALLGHNGAGKSTTISMLVGLLPPTSGDALVFGKNIVSDIDEIRKVLGVCPQQDILFPELTVREHLELFSILKGVEEDSLEGVVTNMADEVSSIVLSLTTLIFITKIYKYS
9.	GgABCA9	MT379670	MMFSISLTALIYGHSIQSSNGMSNIRKLIGVCPQFDILWDALSGQEHLQLFASIKGLPPASIKSITQTSLAEVRLMDAAKVRAGSYSGGMKRRLSVAIALIGNPKLVILDEPTTGMDPITRRHVWDIIENAKRGRAIVLTTHSMEEADILSDRIGIMAKGRLRCIGTSIRLKSRFGTGFVANISFYGNNVEHGPANGDTISTEHREAVKQFFKNVR
10.	GgABCA10	MT379671	MMFSISLTALIYGHSIQSSNGMSNIRKLIGVCPQFDILWDALSGQEHLQLFASIKGLPPASIKSITQTSLAEVRLMDAAKVRAGSYSGGMKRRLSVAIALIGNPKLVILDEPVCKAGYFLSRIYFVDRITRFNLSFYFLDYWYGSNNKEACVGHN
11.	GgABCA11	MT379672	MSLVFSLTGFLLKSSCICLQMIGLTKPTSGTAFVQGLDIRTNMDGIYTSMGVCPQHDLLWESLTGREHLLFYGRLKNLKGSALKQAVEESLKSVNLFHGGVADKQAGKYSGGMKRRLSVAISLIGDPRVVYMDEPSTGLDPASRKCLWNVVKLAKRDRAIILTTHSMEEAEVLCDRLGIFVDGSLQCIGNPKELKGRYGGTYVFTMTTSMDHEEDVENLVQQLSPNANKIYHISGTQKFELPKDEVRIANVFRAVETAKRNFTVSAWGLADTTLEDVFVKVARGAQAFDTLS
12.	GgABCB1	MT379673	MINVSIANHNTTIPNSIPSSLRNRMPISSSLSSPSILPNLNPPRLLQFRTKFQTKQQQQQYLLPKLRIRSSTRTTHTLQFLKPYLVSQHKPILCGWLCSAVSVYSLSNLLSKFSAITTATTAVDAAQGFALGGLVLTRLVATYAQHSLLWEASLNAVYNLRAHVFDRVLHRELAYFEANDAVSAGDIAYRITAEASDLGLTIYALLNTIVPSTLQLSAMIMQMLVISPVLSLISAIVIPCMVLVVAFLGQELRKISKQSHISIAALSAYLNEVLPAILFVKANNAELSECARLKRLALVDCSAKLKKKRMKAVIPQVIQAIYFGVISILCAGSVVISRGSFDRCSLVSFVTSLLFLIEPIQDVAKAYNEWRQGEPAVERLVAMTKFKNKVVEKPDAVHLDHVIGDLKFCDVSFKYNDDMPLILNGLDLHVRTGEIVAIVGPSGGGKTTLVKLLLRLYDPISGSILIDNQNIQNIRLQSLRRHVGVVSQDITLFSGTVAENIGYRDLTTKIDMERVKRVAQTAHADEFIRKLPEGYKTNIGPRGSTLSGGQRQR
13.	GgABCB2	MT379674	MINTFFEPADELRKDSKFWALIFVALSVVAFSFHPLRSYFFAVAGSKLIKRIRLMCFEKIIHMEVGWFDKAEHSSGALGARLSTNAASIRTLVGDALGLLVQDIATVITALVIAFEANWQLSLVILVLLPLLLVNGHVQIRSMQGFSTNAKKLYEEASQVANDAVGNIRTVAAFCAEEKVMELYHKKCVGPVQTGIRQGIVSGTGFGLSLFFLFSVYACSFYAGAQLVKNGKTSISEVFRVFFSLTMAAVAISQSGFMAPGASKARSSAVSVFAILDQKSKIDPSDESGMTLQDVKGEIEFHHVTFKYPTRPSVPILRDLSLTIHSGQTVALVGESGSGKSTVISLLQRFYDPDSGQITLDGTEIQKLQLQWFRQQMGLVSQEPVLFNDTIRANIAYGKGGDATEAEIIAAAELANAHKFISSLQQVKLHSLQWTESVLKKLLNKNVYCKIFE
14.	GgABCB3	MT379675	MWDRFVSHEQGQNSLNRYSSFLITKKLFVQEPKLFRMDISSNIRYGCTRDITQEDVEWAAKQAYAHDFISALPNGYETLVDDDLLSGGQKQRIAIARAILRDPKILILDEATSALDAESEHNVKGVLRSVRSDSASRRSVIVIAHRLSTIQAADRIVVMDGGQVVEVGELLIYFECPKYSYQHSFLKLIILDIFISLFFFNSLTM
15.	GgABCB4	MT379676	MINKHQSIIISTFAGVLRNEVGWFDKPDNTVGSLTSRIISDTAMVKIIIADRMSVIVQCVSSILIATIVSMYVNWRMALVAWAVMPCHFIGGLIQAKSAKGFSGDYSAAHSEIVALASESATNIRTIASFCHEEQVLKKAKTSMEIPKKNYRKESIKYGIIQGFSLCLWNIAHAVALWYTTILVDRGQAKFEDGIRAYQIFSLTVPSITELYTLIPTVVTAINILTPAFQTLDRKTEIEPDIPDDSQPERIQGNVEFENVKFKYPSRPEVTVLDNFSLQIEAGSKVAFVGPSGAGKSSVLALLLRFYDPVAGKVLIDGRDLREYNLRWLRTQIGLVQQEPLLFNSSIRDNICYGNNGASESEIVEVAREANIHEFVSNLPNGYNTVVGEKGCQLSGGQKQRIAIARTLLKKPAILLLDEATSALDAESERIIVNAIKAMNLKEDSGLCSRTTQITVAHRLSTVINSDTIIVMDKGKIVEMGSHSTLIAEDVGLYSRLFRLQSFDETS
16.	GgABCB5	MT379677	MNGLRGERAPLLEAERGTGRGKRHDDASAGQASDLEHGDAVPAANVGFFRVFSLAKPEAGKLVVGTVALLIAATSSILVQKFGGKIIDIVSGDIRTPEEKDEALNAVKSTILEIFLIVVIGSICTALRAWLFYSASERVVARLRKDLFSHLVNQEIAFFDVTRTGELLSRLSEDTQIIKNAATTNLSEAMRNLSTAFIGLSFMFATSWQLTLLALAVVPVISVAVRKFGRFLRELSHKTQAAAAVASSIAEESFGAIRTVRSFAQEDYEVSRYSDKVEETLKLGLKQAKVVGLFSGGLNAASTLSVIIVVIYGANLTIKGAMSSGDLTSFILYSLSVGSSISGLSGLYTVVMKAAGASRRVFQILDRASSMPKSGNKCPLGDQDGEVELDDVWFSYPSRPTHSVLKGITLKLYPGSKVALVGPSGGGKTTIANLIERFYDPTKGKILVNGVPLVEISHKHLHRKISIVSQEPTLFNCSIEENIAYGFDGKVNAADIENAAKMANAHEFISNFPEKYQTFVGERGIRLSGGQKQRIAIARALLMDPKILLLDEATSALDAESEYLVQDAMDSIMKGRTVLVIAHRLSTVKTANTVAVISDGQVVESGTHDELLDKNGVYTALVRRQLQTTKAEI
17.	GgABCB6	MT379678	MVAKASLDGDIASTEMTGSTSHAPVPDPENNQEMGDRQKDSKKSKGKDQSNKTVPFCKLFSFADSWDYLLMFVGTIGAVGNGVSMPLMTIIIGDTIDAFGGNVNTKQIVHEVSKVSLEFAFVGIGAFLAAFLQVACWVITGERQAARIRALYLKAILRQDISFFDKETNSGEVVGRMSGDTVLIQEAMGEKVGKFIQYFAAFLGGLVVAFIKGWLLSLVLLSSLPLLVLSGSILSFAFAKMASRGQAAYSEAATIVERTIGSIRTVASFTGEKQAIAQYNQSLTKAYKTGVQEGVAVGLGLGTVRLFVYSSYALAVWFGGKMVLEKGYTGGEVISVFFAVLTGSLSLGQASPSLTAFAAGQAAAFKMFETIKRQPDIDAYDPAGRQLDDISGDIELREVCFSYPSRPNEMIFTGFSISISSGTTAALVGQSGSGKSTVVSLIERFYDPQAGEVLIDGINLREFQLKWIRQKIGLVSQEPVLFTCTIKENIAYGKDGATDEEIRAAAELANAAKFIDKLPQGLDTMVGEHGTQLSGGQKQRVAIARAILKDPRILLLDEATSALDAESERIVQEALDRIMINRTTVVVAHRLSTIRNVDTIAVIHQGKIVERGSHAELTKDPNGAYSQLIRLQEMKGSEQNVANDSNKPNSLGHSGRQSSQISFSLRSISQGSSGVGNSGRHSFSTSHVVPTTVGFIETADGGPQTPPPPVSSPPEVPFRRLAYLNRPEFPVLVIGTIAAVLHGVILPIFGLLMSEMITVFYKPADELRKDSKVWALVFVAVGVASLLIIPSRYYLFGIAGGKLIKRIRNMCFEKVVHMEVSWFDEAEHSSGALGARLSTDAASVRAMVGDALGLLVQDIATVITALVIAFEANWQLSLVILVLLPLLLVNGHVQIRSMQGFSTNAK
18.	GgABCB7	MT379679	MQQLFLKGFGGDYGHAYSRATSLAREAISNIRTVAAFGAEDRISIQFASELNKPNKQAFLRGHISGFGYGVTQLFAFCSYALGLWYASVLMKKKESNFGDLMKSFMILIITALAIAETLALTPDIVKGSQALGSVFSILHRRTAINPNDPSSKTITEVKGEIKFQNVCFKYPMRPDITIFQNLDLRVPAGKSLAVVGQSGSGKSTVIALVMRFYDPTSGSVLIDDCDIKSLNLRSLRLRIGLVQQEPALFSTTVYENIKYGKEEASEIEVMKAAKAANAHEFISRMPEGYKTEVGERGVQLSGGQKQRVAIARAILKDPSILLLDEATSALDTVSERLVQEALDKLMEGRTTILVAHRLSTVRAADGIAVLQHGRIAEMGSHERLMAKPGSIYKQLVSLQQEKRGQEDH
19.	GgABCB8	MT379680	MSNRTTIIVAHRLSTIRDVDTIIVLKNGQVVESGTHLELMSKNGEYVNLVSLQSSHNLTSSSTISRSGSSRNSSFREPSDNLNHEEELNTRELQSSDRGLPSNTASIPSILDLLKLNAPEWPYAVLGSVGAVMAGMEAPLFALGITHILTAFYSPHGSQIKQEVDRVALIFVGVAVVTIPIYLLQHYFYSLMGERLTARVRLLMFSAILTNEVAWFDLDENN

			TGSLTAMLAADATLVRSALADRLSTIVQNVALTVTAFVIAFTLSWKLTLVVAACLPLLIGASITEQLFLKGFGGDYGHAYSRATSLAREAISNIRTVAAFGAEDRISIQFASELNKPNKQAFLRGHISGFGYGVTQLFAFCSYALGLWYASVLMKKKESNFGDLMKSFMILIITALAIAETLALTPDIVKGSQALGSVFSILHRRTAINPNDPSSKTITEVKGEIKFQNVCFKYPMRPDITIFQNLDLRVPAGKSLAVVGQSGSGKSTVIALVMRFYDPTSGSVLIDDCDIKSLNLRSLRLRIGLVQQEPALFSTTVYENIKYGKEEASEIEVMKAAKAANAHEFISRMPEGYKTEVGERGVQLSGGQKQRVAIARAILKDPSILLLDEATSALDTVSERLVQEALDKLMEGRTTILVAHRLSTVRAADGIAVLQHGRIAEMGSHERLMAKPGSIYKQLVSLQQEKRGQEDH
20.	GgABCB9	MT379681	MLSSRLETDATLLRTIVVDRSTILLQNVGLVVASFIIAFILNWRITLVVLATYPLVISGHISEKLFMKGYGGNLSKAYLKANMLAGEAVSNIRTVAAFCSEEKVMDLYANELVDPSKRSFQRGQIAGIFYGISQFFIFSSYGLALWYGSVLMGKELASFKSVMKSFMVLIVTALAMGETLALAPDLLKGNQMVASVFEVMDRKSGITGDAGEELKTVEGTIELKRIHFSYPSRPDVIIFKDFNLRVPSGKSVALVGQSGSGKSSVISLILRFYDPISGKVLIDGKDITRLNLKSLRKHIGLVQQEPALFATSIYENILYGKEGASDSEVIEAAKLANAHNFISALPEGYSTKVGERGVQLSGGQRQRVAIARAVLKNPEILLLDEATSALDVESERVVQQALDRLMQNRTTIMVAHRLSTIRNADQISVLQDGKIIEQGTHSSLRENKNGAYFKLVNIQQQQHQL
21.	GgABCB10	MT379682	MSSSFQKLFMQGFGGNLSKAYLKANMLAGEAVSNIRTVAAFCAEQKVLDLYANELVDPSKFSFKRGQIAGLFYGISQFFIFSSYGLALWYGSVLMQKELASFKSIMQSFLVLIVTALAMGETLALAPDLLKGNQMAASVFEVMDRKSGIRHDVGEEFKIVEGTIVLKRIHFSYPSRPDVVIFDDFSLIVPSGKSIALVGHSGSGKSSVISLILRFYDPTSGKVMIDGKDIKEIKLKSLRKHIGLVQQEPALFATSIYENILYGKEGASESEVIEAAKLANAHNFISALPEGYSTKAGERGVQLSGGQKQRVAIARAVLRNPKILLLDEATSALDVESERVVQQALDKLMQNRTTVIVAHRLSTIRNADQISVLQDGKIIEQGTHSSLLENTNGAYFKLVNLQQEQRHVQEHYYG
22.	GgABCB11	MT379683	MVGRTTVVVAHRLSTIRNADVIAVVQGGKIVETGNHEQLISNSTSVYASLVQLQEATSLQRLPSIGPSLGRQSSLNYSKELSRTTTSIGGSFRSDKDSIGRVCADEGEKSSKSNHVSAARLYSMVGPDWVYGVFGTFCAFIAGAQMPLFALGISHALVSYYMDWDTTRHEVKKISFLFCGAAVVAVTAYVIEHLSFGIMGERLTLRVRELMFSAILKNEIGWFDDTSNTSSMLSSRLETDATLLKTIVVDRSTILLQNVGLVVTSFVISFILNWRITLVVLATYPLIISGHISEKLFMQGFGGNLSKAYLKANMLAGEAVSNIRTVAAFCAEQKVLDLYANELVDPSKFSFKRGQIAGLFYGISQFFIFSSYGLALWYGSVLMQKELASFKSIMQSFLVLIVTALAMGETLALAPDLLKGNQMAASVFEVMDRKSGIRHDVGEEFKIVEGTIVLKRIHFSYPSRPDVVIFDDFSLIVPSGKSIALVGHSGSGKSSVISLILRFYDPTSGKVMIDGKDIKEIKLKSLRKHIGLVQQEPALFATSIYENILYGKEGASESEVIEAAKLANAHNFISALPEGYSTKAGERGVQLSGGQKQRVAIARAVLRNPKILLLDEATSALDVESERVVQQALDKLMQNRTTVIVAHRLSTIRNADQISVLQDGKIIEQGTHSSLLENTNGAYFKLVNLQQEQRHVQEHYYG
23.	GgABCB12	MT379684	MIGPDWFYGVFGTFGAFITGSLMPLFALGISHALVSYYMDWDTTRHEVKKISFLFCGAAVVAVTAYVIEHLSFGIMGERLTLRVRELMFSAILKNEIGWFDDTSNTSSMLSSRLETDATLLKTIVVDRSTILLQNVGLVVTSFVISFILNWRITLVVLATYPLIISGHISEKLFMQGFGGNLSKAYLKANMLAGEAVSNIRTVAAFCAEQKVLDLYANELVDPSKFSFKRGQIAGLFYGISQFFIFSSYGLALWYGSVLMQKELASFKSIMQSFLVLIVTALAMGETLALAPDLLKGNQMVASVFEVMDRKSGITGDAGEELKTVEGTIELKRIHFSYPSRPDVIIFKDFNLRVPSGKSVALVGQSGSGKSSVISLILRFYDPISGKVLIDGKDITRLNLKSLRKHIGLVQQEPALFATSIYENILYGKEGASDSEVIEAAKLANAHNFISALPEGYSTKVGERGVQLSGGQRQRVAIARAVLKNPEILLLDEATSALDVESERVVQQALDRLMQNRTTIMVAHRLSTIRNADQISVLQDGKIIEQGTHSSLRENKNGAYFKLVNIQQQQHQL
24.	GgABCB13	MT379685	MFYASCGNIVQKLGAGQVMLDGHDIKTLKLRWLRQQIGLVSQEPALFATTIRENILLGRPDANQVEIEEAARVANAHSFIIKLPDGYETQVGERGLQLSGGQKQRIAIARAMLKNPAILLLDEATSALDSESEKLVQEALDRFMIGRTTLVIAHRLSTIRKADLVAVLQQGSVSEIGTHDELFSKGESGAYAKLIKMQEMAHETAMSNARKSSARPSSARNSVSSPIIARNSSYGRSPYSRRLSDFSTSDFSLSLDASHPNYRLEKLAFKEQASSFWRLAKMNSPEWLYALIGSIGSIVCGSLSAFFAYVLSAVLSVYYNPDHRYMIRQIEKYCYLLIGLSSTALLFNTLQHFFWDIVGENLTKRVREKMLTAVLKNEMAWFDQEENESARIAARLTLDANNVRSAIGDRISVIVQNTALMLVACTAGFVLQWRLALVLIAVFPVVVAATVLQKMFMTGFSGDLEAAHVKATQLAGEAIANVRTVAAFNSETKIVGLFTSNLETPLQRCFWKGQISGSGYGIAQFALYASYALGLWYASWLVKHGISDFSKTIRVFMVLMVSANGAAETLTLAPDFIKGGRAMRSVFDLLDRRTEIDPDDQDATPVPDRLRGEVELKHVDFSYPTRPDMPVFRDLNLRVRAGKTLALVGPSGCGKSSVIALIQRFYDPTSGRVMIDGKDIRKYNLKSLRRHISVVPQEPCLFATTIYENIAYGHDSATEAEIIEAATLANAHKFISALPDGYKTFVGERGVQLSGGQKQRIAVARAFVRKAELMLLDEATSALDAESERSVQEALDRASSGKTTIIVAHKLSTIRNANVIAVIDDGKVAEQGSHSQLLKNHSDGIYARMIQLQRFTHSQVIGMASGSSSSTRPKDDEREG
25.	GgABCB14	MT379686	MNSPEWLYALIGSIGSIVCGSLSAFFAYVLSAVLSVYYNPDHRYMIRQIEKYCYLLIGLSSTALLFNTLQHFFWDIVGENLTKRVREKMLTAVLKNEMAWFDQEENESARIAARLTLDANNVRSAIGDRISVIVQNTALMLVACTAGFVLQWRLALVLIAVFPVVVAATVLQKMFMTGFSGDLEAAHVKATQLAGEAIANVRTVAAFNSETKIVGLFTSNLETPLQRCFWKGQISGSGYGIAQFALYASYALGLWYASWLVKHGISDFSKTIRVFMVLMVSANGAAETLTLAPDFIKGGRAMRSVFDLLDRRTEIDPDDQDATPVPDRLRGEVELKHVDFSYPTRPDMPVFRDLNLRVRAGKTLALVGPSGCGKSSVIALIQRFYDPTSGRVMIDGKDIRKYNLKSLRRHISVVPQEPCLFATTIYENIAYGHDSATEAEIIEAATLANAHKFISALPDGYKTFVGERGVQLSGGQKQRIAVARAFVRKAELMLLDEATSALDAESERSVQEALDRASSGKTTIIVAHKLSTIRNANVIAVIDDGKVAEQGSHSQLLKNHSDGIYARMIQLQRFTHSQVIGMASGSSSSTRPKDDEREG
26.	GgABCB15	MT379687	MWTGERQSTKMRIKYLEAALKQDIEFFDTEVRTSDVVFAINTDAVMVQDAISEKLGNFIHYMATFVSGFVVGFTAVWQLALVTLAVVPMIAVIGAIHTTTLAKLSGKSQEALSQAGNIVEQTVAQIRVVLAFVGESRALQAYSSALKVAQKIGYKTGLAKGMGLGATYFVVFCCYALLLWYGGYLVRHHYTNGGLAIATMFAVMIGGLGLGQSAPSMAAFTKARVAAAKIFRIIDHKPGIDKNSESGLELETVTGLVELKNVNFSYPSRPEVRILNDFSLNVPAGKTIALVGSSGSGKSTVVSLIERFYDPTSGQVMLDGHDIKTLKLRWLRQQIGLVSQEPALFATTIRENILLGRPDANQVEIEEAARVANAHSFIIKLPDGYETQVGERGLQLSGGQKQRIAIARAMLKNPAILLLDEATSALDSESEKLVQEALDRFMIGRTTLVIAHRLSTIRKADLVAVLQQGSVSEIGTHDELFSKGESGAYAKLIKMQEMAHETAMSNARKSSARPSSARNSVSSPIITRNSSYGRSPYSRRLSDFSTSDFSLSLDASSYPNYRLEKLPFKEQASSFWRLAKMNSPEWLYALIGSIGSVVCGSLSAFFAYVLSAVLSVYYNPNHRHMIREIEKYCYLLIGLSSAALLFNTLQHFFWDIVGENLTKRVREKMLTAVLKNEMAWFDQEENESARIAARLALDANNVRSAIGDRISVIVQNTALMLVACTAGFVLQWRLALVLVAVFPVVVAATVLQKMFMTGFSGDLEAAHAKATQLAGEAIANVRTVAAFNSEKKIVGLFTSNLEIPLRRCFWKGQISGSGYGIAQFALYASYALGLWYASWLVKHGISDFSKTIRVFMVLMVSANGAAETLTLAPD

			FIKGGRAMKSVFDLLDRRTEIEPDDPDATPVPDRLRGEVELKHVDFSYPTRPDMPVFRDLSLRARAGKTLALV<br>GPSGCGKSSVIALIQRFYDPTSGRVMIDGKDIRKYNLKSLRRHIAVVPQEPCLFASTIYENIAYGHDSATEAEIV<br>EAATLANAHKFISSLPDGYKTFVGERGVQLSGGQKQRIAIARAFVRKAELMLLDEATSALDAESERSVQEALD<br>RACSGKTTIIVAHRLSTIRNANVIAVIDDGKVAEQGSHSHLLKNHPDGIYARMIQLQRFTNSQVVGVAPGSSSS<br>GRPKDDEREG
27.	GgABCB16	MT379688	MSYIQVLSFQSSGIKLGTFKSLNESLTRVAVYISLIALYCLGGSKVKAGELSVGTMASFIGYTFTITFAVQGLVN<br>TFGDLRGTFAAVERINSVLSGVQVDDALAYGLERELKQKAVDDENLELFFSNGSAGTNQKNYLHYMSALKT<br>SSNLLNLAWSGDVCLEDVHFSYPLRPDVEILSGLNLRLKCGTVTALVGPSGAGKSTIVQLLSRFYEPARGCITV<br>GGEDVRTFDKTEWARVVSIVNQEPVLFSVSVGENIAYGLPDDNVSKDDVIKAAKAANAHDFIISLPQGYDTL<br>VGERGGLLSGGQRQRIAIARALLKNAPILILDEATSSLDAVSERLVQDALNHLMKGRTTLVIAHRLSTVQNAY<br>QIALCSDGRIAELGTHFELLAKKGQYASLVATQRLAFE
28.	GgABCB17	MT379689	MPVLEHLNFSVEENQVIAIGGLSGSGKSTLINLLLRLYEPSSGQIYIDGFPLKELDIRWLRQNIGYVAQEPHLFH<br>MDIKSNIKYGCPRNNITQEDIEGAAKQAYAHVFISSLPNGYETLVDDNALSGGQKQRIVIARAILRDPIIMILDE<br>ATSAQDSESEHYIKEVLYALKDEAKSRTIIIIAHRLSTIKAADRILVMDNGRIIEMGSHEELLLKDGLYAKLNKI<br>QADILT
29.	GgABCB18	MT379690	MSLGQASPSMSAFAAGQAAAYKMFQTIERKPEIDAYDPSGKILEDIHGEIDLRGVYFSYPARPEELIFNGFSLHI<br>PSGTTAALVGQSGSGKSTVISLIERFYDPQAGEVLIDGINVKEFQLRWIRGKIGLVSQEPALFASSIKDNIAYGK<br>EGATVEEIRAAAELANAAKFIDKLPQVLVYS
30.	GgABCB19	MT379691	MMVSRGLFGWSPPHIQPLTPVSEVSEPPESPSPYMDFGTETSASQQVEMEEEMEEPDEIEPPPEAVPFSRLFDY<br>ADRLDWFLMVVGSLAAAAHGTALVVYLHYFAKVIRVPQEQDQFHRFKELALTIVYIAAGVFAAGWIEVSCW<br>ILTGERQTAVIRSKYVRVLLNQDMSFFDTYGNNGDIVSQVLSDVLLIQSALSEKVGNYIHNMATFFSGLVIAFI<br>NCWQIALITLATGPFIVAAGGISNIFLHRLAENIQDAYAEAATIAEQAVSYIRTLYSFTNETLAKYSYATSLQAT<br>LRYGILISLVQGLGLGFTYGLAICSCALQLWVGRFLVIHGKAHGGEIITALFAVILSGLGLNQAATNFYSFDQG<br>RIAAYRLFEMISRSSSSSNHDGGAPAFVQGIIEFRNVYFSYLSRPEIPILSEFYLTVPAKKAVALVGRNGSGKSSII<br>PLMERFYDPTLGEVLLDGENIKNLKLEWLRSQIGLVTQEPALLSLSIRDNIAYGRDTTMDQIEEAAKIAHAHTF<br>ISSLDKGYDTQIGRAGLALSEEQKIKLSIARAVLLNPSILLLDEVTGGLDFEAERSVQEALDLLMLGRSTIIIARR<br>LSLIKNADYIGVMEEGQLVEMGTHDELLTLNGLYAELVRCEEATKLPKRSTSACNHASAFAMLYYCLCL
31.	GgABCB20	MT379692	MINTFFEPADELRKDSKFWALIFVALSVVAFSFHPLRSYFFAVAGSKLIKRIRLMCFEKIIHMEVGWFDKAEHS<br>SGALGARLSTNAASIRTLVGDALGLLVQDIATVITALVIAFEANWQLSLVILVLLPLLLVNGHVQIRSMQGFST<br>NAKKLYEEASQVANDAVGNIRTVAAFCAEEKVMELYHKKCVGPVQTGIRQGIVSGTGFGLSLFFLFSVYACS<br>FYAGAQLVKNGKTSISEVFRVFFSLTMAAVAISQSGFMAPGASKARSSAVSVFAILDQKSKIDPSDESGMTLQ<br>DVKGEIEFHHVTFKYPTRPSVPILRDLSLTIHSGQTVALVGESGSGKSTVISLLQRFYDPDSGQITLDGTEIQKLQ<br>LQWFRQQMGLVSQEPVLFNDTIRANIAYGKGGDATEAEIIAAAELANAHKFISSLQQGYDTVVGERGVQLSG<br>GQKQRVAIARAIVKSPKILLLDEATSALDAESEKVVQDALDRVRVDRTTIVVAHRLSTIKNADSIAVVKTGVI<br>AEKGKHDTLLNKGGIYASLVALHTSASSSS
32.	GgABCB21	MT379693	MAAVAISQSGFMAPGASKARSSAVSVFAILDQKSKIDPSDESGMTLQDVKGEIEFHHVTFKYPTRPSVPILRDL<br>SLTIHSGQTVALVGESGSGKSTVISLLQRFYDPDSGQITLDGTEIQKLQLQWFRQQMGLVSQEPVLFNDTIRAN<br>IAYGKGGDATEAEIIAAAELANAHKFISSLQQGYDTVVGERGVQLSGGQKQRVAIARAIVKSPKILLLDEATS<br>ALDAESEKVVQDALDRVRVDRTTIVVAHRLSTIKNADSIAVVKTGVIAEKGKHDTLLNKGGIYASLVALHTS<br>ASSSS
33.	GgABCB22	MT379694	MWELVARDRWVIITAFSTLVIAAVSEISIPHFLTASIFSAQGADITVFHRNVRLLGLLCIISGICSGIRGCCFSIAN<br>MILVKRMRETLYSSLLLQDISFFDSATVGDLTSRLGADCQQVSRVIGNDLNLIMRNILQGAGSLIYLLILSWPL<br>GLCTLTICSTLAAVMLRYGRYQKKAARLIQEVTASANDVAQETFSLIRTVRVYGTEEEELGRYNWWLGKLV<br>DISLRQSAAYGFWNFSFNTLYHSTQVIAVLFGGMSILAGHITAEKLTKFILYSEWLIYSTWWVGDNISNLMQS<br>VGASEKVFHLMDLSPSSQFIERGIKLQRLTGHIEFLNVSFHYPSRPTVSVVRHVNFSVHPSEVVAIVGLSGSGKS<br>TLVNLLLRLYEPTSGQILIDGIPLKDFDITWWRERVGYVGQEPKLFRMDISSNIRYGCTRDITQEDVEWAAKQ<br>AYAHDFISALPNGYETLVDDDLLSGGQKQRIAIARAILRDPKILILDEATSALDAESEHNVKGVLRSVRSDSAS<br>RRSVIVIAHRLSTIQAADRIVVMDGGQVVENGSHRELLLKDGLYARLIRKQADAMA
34.	GgABCB23	MT379695	MILVKRMRETLYSSLLLQDISFFDSATVGDLTSRLGADCQQVSRVIGNDLNLIMRNILQGAGSLIYLLILSWPL<br>GLCTLTICSTLAAVMLRYGRYQKKAARLIQEVTASANDVAQETFSLIRTVRVYGTEEEELGRYNWWLGKLV<br>DISLRQSAAYGFWNFSFNTLYHSTQVIAVLFGGMSILAGHITAEKLTKFILYSEWLIYSTWWVGDNISNLMQS<br>VGASEKVFHLMDLSPSSQFIERGIKLQRLTGHIEFLNVSFHYPSRPTVSVVRHVNFSVHPSEVVAIVGLSGSGKS<br>TLVNLLLRLYEPTSGQILIDGIPLKDFDITWWRERVGYVGQEPKLFRMDISSNIRYGCTRDITQEDVEWAAKQ<br>AYAHDFISALPNGYETLVDDDLLSGGQKQRIAIARAILRDPKILILDEATSALDAESEHNVKGVLRSVRSDSAS<br>RRSVIVIAHRLSTIQAADRIVVMDGGQVVENGSHRELLLKDGLYARLIRKQADAMA
35.	GgABCB24	MT379696	MIMEKGYNGGTVINVIIAVLTASMSLGQASPSMSAFAAGQAAAYKMFQTIARKPEIDAYDPNGKILEDIQGEI<br>KLRDVYFSYPARPEELIFNGFSLHIPSGSTAALVGQSGSGKSTVISLVERFYDPQGGEVLIDGINLKEFQLRWIR<br>GKIGLVSQEPVLFASSIKANIAYGKDGATIEEIRSASELANAAKFIDKLPQGLDTMVGDHGTQLSGGQKQRIAI<br>ARAILKDPRILLLDEATSALDAESERVVQEALDRIMVNRTTVVVAHRLSTVRNADMIAVIHRGKMVEKGTTP<br>LHFSEIFNSCTESRIQIITI
36.	GgABCB25	MT379697	MAKGLGLGCTYGIACMSWALVFWYAGVFIRNGQTDGGKAFTAIFSAIVGGMSLGQSFSNLGAFSKGKAAGY<br>KLMEIIRQKPTIVEDPSDCKCLPEVNGNIEFKDVTFSYPSRPDVIIFRNFSIFFPAGKTVAVVGGSGSGKSTVVSL<br>IERFYDPNEGQVLLDNVDIKTLQLKWLRDQIGLVNQEPALFATTILENILYGKPDATIAEVEAAASAANAHSFI<br>TLLPNGYNTQVGERGVQLSGGQKQRIAIARAMLKNPKILLLDEATSALDAGSESIVQEALDRLMVGRTTVVV<br>AHRLSTIRNVDTIAVIQQGQVVETGTHEELIAKAGAYASLIRFQEMVGNRDFSNPSTRRNRSSRLSHSLSTKSL<br>SLRSGSLRNLSYQYSTGADGRIEMISNAETDKKNPAPEGYFFRLLKLNAPEWPYSIMGAVGSVLSGFIGPTFAI<br>VMSNMIEVFYYRNYASMERKTKEYVFIYIGAGLYAVGAYLIQHYFFSIMGENLTTRVRRMMLAAILRNEVG<br>WFDEEEHNSSLVAARLATDAADVKSAIAERISVILQNMTSLLTSFIVAFIVEWRVSLLILATFPLLVLANFAQV<br>KKNTHTPNLFSISHHIY
37.	GgABCB26	MT379698	MFICFNRSLGQSFSNLGAFSKGKAAGYKLMEIIRQKPTIVEDPSDCKCLPEVNGNIEFKDVTFSYPSRPDVIIFR<br>NFSIFFPAGKTVAVVGGSGSGKSTVVSLIERFYDPNEGQVLLDNVDIKTLQLKWLRDQIGLVNQEPALFATTIL

			ENILYGKPDATIAEVEAAASAANAHSFITLLPNGYNTQVGERGVQLSGGQKQRIAIARAMLKNPKILLLDEAT<br>SALDAGSESIVQEALDRLMVGRTTVVVAHRLSTIRNVDTIAVIQQGQVVETGTHEELIAKAGAYASLIRFQEM<br>VGNRDFSNPSTRRNRSSRLSHSLSTKSLSLRSGSLRNLSYQYSTGADGRIEMISNAETDKKNPAPEGYFFRLLK<br>LNAPEWPYSIMGAVGSVLSGFIGPTFAIVMSNMIEVFYYRNYASMERKTKEYVFIYIGAGLYAVGAYLIQHYF<br>FSIMGENLTTRVRRMMLAAILRNEVGWFDEEEHNSSLVAARLATDAADVKSAIAERISVILQNMTSLLTSFIV<br>AFIVEWRVSLLILATFPLLVLANFAQQLSLKGFAGDTAKAHAKTSMIAGEGVSNIRTVAAFNAQNKMLSIFCH<br>ELRVPQSQSLRRSQMSGLLFGLSQLALYASEALILWYGAHLVSRGASTFSKVIKVFVVLVITANSVAETVSLAP<br>EIIRGGEAVGSVFSILDRATRIDPDDPDAEPVESLRGEIELRHVDFAYPSRPDVMVFKDLSIRIRAGQSQALVGA<br>SGSGKSSVIVLIERFYDPIAGKVMVDGKDIRKLNLKSLRLKIGLVQQEPALFAASIFDNIAYGKEGATEAEVIEA<br>ARAANVHGFVSSLPEGYKTPVGERGVQLSGGQKQRIAIARAVLKDPTILLLDEATSALDAESECVLQEALERL<br>MRGRTTVLVAHRLSTIRGVDCIGVVQDGRIVEQGSHSELISRPEGAYSRLLQLQHHHI
38.	GgABCB27	MT379699	MIAGEGVSNIRTVAAFNAQNKMLSIFCHELRVPQSQSLRRSQMSGLLFGLSQLALYASEALILWYGAHLVSRG<br>ASTFSKVIKVFVVLVITANSVAETVSLAPEIIRGGEAVGSVFSILDRATRIDPDDPDAEPVESLRGEIELRHVDFA<br>YPSRPDVMVFKDLSIRIRAGQSQALVGASGSGKSSVIVLIERFYDPIAGKVMVDGKDIRKLNLKSLRLKIGLVQ<br>QEPALFAASIFDNIAYGKEGATEAEVIEAARAANVHGFVSSLPEGYKTPVGERGVQLSGGQKQRIAIARAVLK<br>DPTILLLDEATSALDAESECVLQEALERLMRGRTTVLVAHRLSTIRGVDCIGVVQDGRIVEQGSHSELISRPEG<br>AYSRLLQLQHHHI
39.	GgABCB28	MT379700	MGSQKEEEKKDHQQLGGSSSSSSIGIILRYADWVDIVLMLMGALGAIGDGMSTNVLLLFASRIMNSLGYSNN<br>KQNGGHTSMAEVEKCSLYFVYLGLAAMVVAFMEGYCWSKTSERQVLRIRYKYLEAVLKQEVGFFDSQEEA<br>TTSEIINSISKDTSLIQEVLSEKVPLFLMHSSSFISGVSFATYFSWRLALVAFPTLLLLIIPGMIYGKYLIYLTRSSL<br>KEYGKANAIVQQALSSIKTVYAFTAEKRIIGRYSEILDTTSRLGIKQGIAKGLAVGSTGLSFAIWAFLAWYGSH<br>LVMYKGESGGRIYAAGISFIMSGLSLGVVLPDLKYFTEASVAASRIFAMIDRTPTIDGTEDTTKGIVLDSISGRL<br>DFEHVKFTYPCRPDMVVLSDFNLQVEAGKTVALVGASGSGKSTAIALVQRFYDADEGVVRVDGVDIKSLNL<br>KWVRGKMGLVSQEHALFGTSIKENIMFGKIDATMDEIVAAATAANAHNFIRQLPEGYETKIGERGAFLSGGQ<br>KQRIAIARAIIKNPVILLLDEATSALDSESELLVQNALDQASMGRTTLVVAHKLSTIRNADLIAVVNGGCIIETG<br>THNELINRPNGHYAKLAKLQTQMSITSDQDQNSEQQGIILLSAARSSAGRSSTAKSSPAIFPKSPLLHDNDSDTI<br>TPSLIVSHIPPPSFPRLLSLNAPEWKQGLIGTLSAIAFGSVQPLYALTIGGMISAFFAESHHEMRNRIRTYSLVFSS<br>LSLASIILNLLQHYNFAYMGAKLTKRIRLCMLEKILTFETAWFDEEKNSSGALCSRLSNEASMVKSLVADRLS<br>LLVQTASAVTIAMIIGIVVAWKLALVMIAVQPLTILCFYTRKVLLSTLSTKFVKAQNQSTQIAVEAVYNHRIVT<br>SFGSITKVLRLFDEAQEEPRKEARKKSWLAGIGMGSAQCLTFMSWALDFWYGGTLVEKGEITAGDVFKTFFV<br>LVSTGKVIADAGSMTSDLAKSSTTVASVFEILDRKSLIPKVGDGTNGIKLEKMGGKIELKNVDFAYPSRARTPI<br>LRKFCLEVKPGKSVGLVGKSGCGKSTVIALIQRFYDVERGSVRVDNVDIRELDIHWYRQHTALVSQEPVIYSG<br>SIRDNILFGKPDATENEVVEAARAANAHEFISSLKDGYETECGERGVQLSGGQKQRIAIARAIIRNPTILLLDEA<br>TSALDVQSEQVVQEALDRTMVGRTTIVVAHRLNTIKELDSIAYVSEGKVLEQGTYSQLKHKRGAFFNLASHQI<br>QT
40.	GgABCB29	MT379701	MTFEIGWFDHEENTSASICARLSSEANLVRSLVGDRMSLLAQAVFGSVFAYTVGLVLTWRLSLVMIAVQPLVI<br>GSFYSRSVLMKTMAEKTRKAQREGSQLASEAVINHRTITAFSSQRRMLSLFKSTMVGPKKESIRQSWISGFGL<br>FSSQFFNTASTALAYWYGGKLLIEGLIEPRHLFQAFLILLFSAYIIAEAGSMTSDISKGSSAVGSVFAILDRKSEV<br>DPEALWGADKKRKIRGRVELRSVFFAYPTRPDQMIFQGLNLKVEAGRTVALVGHSGCGKSTIIALIERFYDPL<br>KGMVCIDEQDIKSYNLRILRSHIALVNQEPTLFSGTIRENIAYGKENATESEIRRAATLANAHEFISGMNDGYET<br>YCGERGVQLSGGQKQRVALARAILKNPAILLLDEATSALDSVSEILVQEALEKIMVGRTCIAVAHRLSTIQKSN<br>SIAVIKNGKVVEQGSHNELISLGRNGAYYSLVKLQSGSSPR
41.	GgABCB30	MT379702	MMVSRGLFGWSPPHIQPLTPVSEVSEPPESPSPYMDFGTETSASQQVEMEEEMEEPDEIEPPPEAVPFSRLFDY<br>ADRLDWFLMVVGSLAAAAHGTALVVYLHYFAKVIRVPQEQDQFHRFKELALTIVYIAAGVFAAGWIEVSCW<br>ILTGERQTAVIRSKYVRVLLNQDMSFFDTYGNNGDIVSQVLSDVLLIQSALSEKVGNYIHNMATFFSGLVIAFI<br>NCWQIALITLATGPFIVAAGGISNIFLHRLAENIQDAYAEAATIAEQAVSYIRTLYSFTNETLAKYSYATSLQAT<br>LRYGILISLVQGLGLGFTYGLAICSCALQLWVGRFLVIHGKAHGGEIITALFAVILSGLGLNQAATNFYSFDQG<br>RIAAYRLFEMISRSSSSSNHDGGAPAFVQGIIEFRNVYFSYLSRPEIPILSEFYLTVPAKKAVALVGRNGSGKSSII<br>PLMERFYDPTLGEVLLDGENIKNLKLEWLRSQIGLVTQEPALLSLSIRDNIAYGRDTTMDQIEEAAKIAHAHTF<br>ISSLDKGYDTQIGRAGLALSEEQKIKLSIARAVLLNPSILLLDEVTGGLDFEAERSVQEALDLLMLGRSTIIIARR<br>LSLIKNADYIGVMEEGQLVEMGTHDELLTLNGLYAELVRCEEATKLPKRMPVRNYKKTAAFQIEKDSSESHS<br>FKEPSSPRMIKSPSLQRISTVFRPSDGFFNSQESPQVRSPPPEKMMENGQSLDSTEKEPSIKRQDSFEMRLPELPK<br>IDVQSVHRQTSKGSDPESPVSPLLISDPKNERSHSQTFSRPDSHSDDFLVKMNETKDARHRDQPSLWRLAELSF<br>AEWLYAVLGSIGAAIFGSFNPLLAYVIGLVVTAYYEIDETHHMRGEIDKWCLIIACMGIVTVFANFLQHFYFGI<br>MGEKMTERVRRMMFSAMLRNETGWYDEEENSADNLSMRLANDATFVRAAFSNRLSIFIQDSAAVIVAFLIG<br>VLLHWRLALVALATLPILCVSAIAQKLWLAGFSRGIQEMHRKASLVLEDAVRNIYTVVAFCAGNKVMELYR<br>LQLNRIFKQSFLHGLAIGFAFGFSQFLLFACNALLLWYTAMCVKESYVNPPTALKAYMVFSFATFALVEPFGL<br>APYILKRRKSLISVFEIIDRVPKIDPDDSSALKPPNVYGSIEFKNVDFCYPTRPEVLVLSNFSLKVSGGQTIAVVG<br>VSGSGRSTIISLIERFYDPVAGQVLLDGRDLKQYNLRWLRSHLGLVQQEPIIFSTTIRENIIYARHNASEAEMKE<br>AARIANAHHFISSLPNGYDTHVGMRGVDLTPGQKQRIAIARVVLKNAPILLLDEASSSIESESSRVVQEALDTLI<br>MGNKTTILIAHRAAMMKHVDSIVVLNGGRIVEEGTHDSLAAKNGLYVRLMQPHFGKALRQHRLV
42.	GgABCB31	MT379703	MGLVSQEPVLFNDTIRANIAYGKGGDATEAEIIAAAELANAHRFISGLQQGYDTVVGERGTQLSGGQKQRVAI<br>ARAIIKSPKILLLDEATSALDAESERVVQDALDKVMVSRTTVVVAHRLSTIKNADVIAVVKNGVIVEKGRHET<br>LINVKDGFYASLVQLHTSASTV
43.	GgABCB32	MT379704	MITCLLIFFRGEGLYFQLFPFKCDHIFISKTGAPVFQVGRAGIALTEEQKIKLSIARAVLLNPSILLLDEVTGGLD<br>FEAERAVQEALDLLMLGRSTIIIARRLSLIRNADYIAVMDEGQLVEMGTHDELLNLDGLYAELLRCEEAAKLP<br>KRMPVRNYKETAAFQIEKDSSASHSFNEPSSPKLIKSPSLQRISNVSRPPDGIFNLQESPKVLSPPPEKMLENGQ<br>ALDAADKEPSIRRQDSFEMRLPELPKIDVQSVHRQKSNDSDPESPVSPLLTSDPKNERSHSQTFSRPHSHSDDA<br>SVTMRGEKDARHRKPPSLQKLAELSFAEWLYAVLGSIGAALFGSFNPALAYVIGLVVTAYYRINDQHHLEREI<br>DKWCLIIGCMGIVTVIANFLQHFYFGIMGEKMTERVRRMMFSAMLRNEVGWFDDEENSADNLSMRLANDAT<br>FVRAAFSNRLSIFIQDSAAVIVGLLIGALLHWRLALVAFATLPVLCVSAVAQKLWLAGFSRGIQEMHRKASLV<br>LEDAVRNIYTVVAFCAGNKVMDLYRLQLKKIFKQSFLHGMAIGFAFGFSQFLLFACNALLLWYTAICIKNGY

			MDPPTALKEYMVFSFATFALVEPFGLAPYILKRRKSLISVFEIIDRVPKIDPDDNTALKPPNVYGSIELKNVDFCYPSRPEVLVLSNFSLKVNGGQTVAIVGVSGSGKSTIISLIERFYDPVAGQVLLDGRDLKLYNLRWLRSHLGLVQQEPIIFSTTIRENIIYARHNASEAEMKEAARIANAHHFISSLPHGYDTHVGMRGVDLTPGQKQRIAIARVVLKNAPILLLDEASSSIESESSRVIQEALDTLIMGNKTTILIAHRAAMMRHVDNIVVLNGGRIVEEGTHDSLAAKNGLYVRLMQPHFGKALRQHRLV
44.	GgABCB33	MT379705	MMISRGLFGWSPPHVQPLTPVSEVSEPPESPSPYLDPGAETSTSQQVEVEEEMEEPEEIEPPPAAVPFSRLFACADRFDWFLMAVGSVAAAAHGTALVVYLHYFAKIIHVLRMDPQHNTSQERFDRFTELALTIVYIAVGVFAAGWIEVSCWILTGERQTAVIRSKYVQVLLNQDMSFFDTYGNNGDIVSQVLSDVLLIQSALSEKVGNYIHNMATFFSGLVVGLINCWQIALITLATGPFIVAAGGISNIFLHRLAENIQDAYAEAASIAEQAVSYVRTLYAFTNETLAKYSYATSLQATLRYGILISLVQGLGLGFTYGLAICSCALQLWVGRFLVIHGKAHGGEIVTALFAVILSGLGLNQAATNFYSFDQGRIAAYRLYEMISRSSSSVNHDGTAPDSVQGNIEFRNVYFSYLSRPEIPILSGFYLTVPSKRTVALVGRNGSGKSSIIPLMERFYDPTLGEVLLDGENIKNLKLEWLRSQIGLVTQEPALLSLSIRDNIAYGRDVTMDQIEEAAKIAHAHTFISSLGKGYDTQVGRAGIALTEEQKIKLSIARAVLLNPSILLLDEVTGGLDFEAERAVQEALDLLMLGRSTIIIARRLSLIRNADYIAVMDEGQLVEMGTHDELLNLDGLYAELLRCEEAAKLPKRMPVRNYKETAAFQIEKDSSASHSFNEPSSPKLIKSPSLQRISNVSRPPDGIFNLQESPKVLSPPPEKMLENGQALDAADKEPSIRRQDSFEMRLPELPKIDVQSVHRQKSNDSDPESPVSPLLTSDPKNERSHSQTFSRPHSHSDDASVTMRGEKDARHRKPPSLQKLAELSFAEWLYAVLGSIGAALFGSFNPALAYVIGLVVTAYYRINDQHHLEREIDKWCLIIGCMGIVTVIANFLQHFYFGIMGEKMTERVRRMMFSAMLRNEVGWFDDEENSADNLSMRLANDATFVRAAFSNRLSIFIQDSAAVIVGLLIGALLHWRLALVAFATLPVLCVSAVAQKLWLAGFSRGIQEMHRKASLVLEDAVRNIYTVVAFCAGNKVMDLYRLQLKKIFKQSFLHGMAIGFAFGFSQFLLFACNALLLWYTAICIKNGYMDPPTALKEYMVFSFATFALVEPFGLAPYILKRRKSLISVFEIIDRVPKIDPDDNTALKPPNVYGSIELKNVDFCYPSRPEVLVLSNFSLKVNGGQTVAIVGVSGSGKSTIISLIERFYDPVAGQVLLDGRDLKLYNLRWLRSHLGLVQQEPIIFSTTIRENIIYARHNASEAEMKEAARIANAHHFISSLPHGYDTHVGMRGVDLTPGQKQRIAIARVVLKNAPILLLDEASSSIESESSRVIQEALDTLIMGNKTTILIAHRAAMMRHVDNIVVLNGGRIVEEGTHDSLAAKNGLYVRLMQPHFGKALRQHRLV
45.	GgABCB34	MT379706	MQAVSYVRTLYAFTNETLAKYSYATSLQATLRYGILISLVQGLGLGFTYGLAICSCALQLWVGRFLVIHGKAHGGEIVTALFAVILSGLGLNQAATNFYSFDQGRIAAYRLYEMISRSSSSVNHDGTAPDSVQGNIEFRNVYFSYLSRPEIPILSGFYLTVPSKRTVALVGRNGSGKSSIIPLMERFYDPTLGEVLLDGENIKNLKLEWLRSQIGLVTQEPALLSLSIRDNIAYGRDVTMDQIEEAAKIAHAHTFISSLGKGYDTQVGRAGIALTEEQKIKLSIARAVLLNPSILLLDEVTGGLDFEAERAVQEALDLLMLGRSTIIIARRLSLIRNADYIAVMDEGQLVEMGTHDELLNLDGLYAELLRCEEAAKLPKRMPVRNYKETAAFQIEKDSSASHSFNEPSSPKLIKSPSLQRISNVSRPPDGIFNLQESPKVLSPPPEKMLENGQALDAADKEPSIRRQDSFEMRLPELPKIDVQSVHRQKSNDSDPESPVSPLLTSDPKNERSHSQTFSRPHSHSDDASVTMRGEKDARHRKPPSLQKLAELSFAEWLYAVLGSIGAALFGSFNPALAYVIGLVVTAYYRINDQHHLEREIDKWCLIIGCMGIVTVIANFLQHFYFGIMGEKMTERVRRMMFSAMLRNEVGWFDDEENSADNLSMRLANDATFVRAAFSNRLSIFIQDSAAVIVGLLIGALLHWRLALVAFATLPVLCVSAVAQKLWLAGFSRGIQEMHRKASLVLEDAVRNIYTVVAFCAGNKVMDLYRLQLKKIFKQSFLHGMAIGFAFGFSQFLLFACNALLLWYTAICIKNGYMDPPTALKEYMVFSFATFALVEPFGLAPYILKRRKSLISVFEIIDRVPKIDPDDNTALKPPNVYGSIELKNVDFCYPSRPEVLVLSNFSLKVNGGQTVAIVGVSGSGKSTIISLIERFYDPVAGQVLLDGRDLKLYNLRWLRSHLGLVQQEPIIFSTTIRENIIYARHNASEAEMKEAARIANAHHFISSLPHGYDTHVGMRGVDLTPGQKQRIAIARVVLKNAPILLLDEASSSIESESSRVIQEALDTLIMGNKTTILIAHRAAMMRHVDNIVVLNGGRIVEEGTHDSLAAKNGLYVRLMQPHFGKALRQHRLV
46.	GgABCB35	MT379707	MFTFANQVLSFQSSGIKLGTFKSLNESLTRVAVYISLIALYCLGGSKVKAGELSVGTMASFIGYTFTITFAVQGLVNTFGDLRGTFAAVERINSVLSGVQVDDALAYGLERELKQKAVDDENLELFFSNGSAGTNQKNYLHYMSALKTSSNLLNLAWSGDVCLEDVHFSYPLRPDVEILSGLNLRLKCGTVTALVGPSGAGKSTIVQLLSRFYEPARGCITVGGEDVRTFDKTEWARVVSIVNQEPVLFSVSVGENIAYGLPDDNVSKDDVIKAAKAANAHDFIISLPQGYDTLVGERGGLLSGGQRQRIAIARALLKNAPILILDEATSSLDAVSERLVQDALNHLMKGRTTLVIAHRLSTVQNAYQIALCSDGRIAELGTHFELLAKKGQYASLVATQRLAFE
47.	GgABCB36	MT379708	MASFIGYTFTITFAVQGLVNTFGDLRGTFAAVERINSVLSGVQVDDALAYGLERELKQKAVDDENLELFFSNGSAGTNQKNYLHYMSALKTSSNLLNLAWSGDVCLEDVHFSYPLRPDVEILSGLNLRLKCGTVTALVGPSGAGKSTIVQLLSRFYEPARGCITVGGEDVRTFDKTEWARVVSIVNQEPVLFSVSVGENIAYGLPDDNVSKDDVIKAAKAANAHDFIISLPQGYDTLVGERGGLLSGGQRQRIAIARALLKNAPILILDEATSSLDAVSERLVQDALNHLMKGRTTLVIAHRLSTVQNAYQIALCSDGRIAELGTHFELLAKKGQYASLVATQRLAFE
48.	GgABCB37	MT379709	MLEKAQEGPIHENMRQSWFAGFGLGCSQFLMSCTWALDYWYGGKLVSDGYITKKAMFESFMVVVSTGRVIADAGSMTSDLAKGADAVESIFSILDRCTTIEPDDPNGHKPETLVGQIELHDVHFAYPARPNVVIFQGFSIKIEAGKSTALVGQSGSGKSTIIGLIERFYDPLKGTVTMDGIDIKSYNLKSLRKHIALVSQEPALFSGTIRENIAYGIIGGDMIDEDEIIEAARVANAHDFISSLRDGYETWCGDKGVQLSGGQKQRIAIARAMLKDPKVLLLDEATSALDNQSERVVQDALTKVMVGRTSVVVAHRLSTIHNCDLIAVLDKGKMVEIGTHSALLAKGPCGAYYSLVRLQTRHDAATPN
49.	GgABCB38	MT379710	MISRSSSSVNHDGTAPDSVQGNIEFRNVYFSYLSRPEIPILSGFYLTVPSKRTVALVGRNGSGKSSIIPLMERFYDPTLGEVLLDGENIKNLKLEWLRSQIGLVTQEPALLSLSIRDNIAYGRDVTMDQIEEAAKIAHAHTFISSLGKGYDTQVGRAGIALTEEQKIKLSIARAVLLNPSILLLDEVTGGLDFEAERAVQEALDLLMLGRSTIIIARRLSLIRNADYIAVMDEGQLVEMGTHDELLNLDGLYAELLRCEEAAKLPKRMPVRNYKETAAFQIEKDSSASHSFNEPSSPKLIKSPSLQRISNVSRPPDGIFNLQESPKVLSPPPEKMLENGQALDAADKEPSIRRQDSFEMRLPELPKIDVQSVHRQKSNDSDPESPVSPLLTSDPKNERSHSQTFSRPHSHSDDASVTMRGEKDARHRKPPSLQKLAELSFAEWLYAVLGSIGAALFGSFNPALAYVIGLVVTAYYRINDQHHLEREIDKWCLIIGCMGIVTVIANFLQHFYFGIMGEKMTERVRRMMFSAMLRNEVGWFDDEENSADNLSMRLANDATFVRAAFSNRLSIFIQDSAAVIVGLLIGALLHWRLALVAFATLPVLCVSAVAQVCTYSSGFPNIITFPCHEPTIPQAYVVG
50.	GgABCB39	MT379711	MVGRTTVVVAHRLSTIRNADVIAVVQGGKIVETGNHEQLISNSTSVYASLVQLQEATSLQRLPSIGPSLGRQSSLNYSKELSRTTTSIGGSFRSDKDSIGRVCADEGEKSSKSNHVSAARLYSMVGPDWVYGVFGTFCAFIAGAQMPLFALGISHALVSYYMDWDTTRHEVKKIAFLFCGAAVLTVTVHAIEHLSFGIMGERLTLRVREMMFSAILKNEIGWFDDTSNTSSMLSSRLETDATLLRTIVVDRSTILLQNVGLVVASFIIAFILNWRITLVVLATYPLVISGHISEKLFMKGYGGNLSKAYLKANMLAGEAVSNIRTVAAFCSEEKVMDLYANELVDPSKRSFQRGQIAGIFYGISQFFI

			FSSYGLALWYGSVLMGKELASFKSVMKSFMVLIVTALAMGETLALAPDLLKGNQMVASVFEVMDRKSGITGDAGEELKTVEGTIELKRIHFSYPSRPDVIIFKDFNLRVPSGKSVALVGQSGSGKSSVISLILRFYDPISGKVLIDGKDITRLNLKSLRKHIGLVQQEPALFATSIYENILYGKEGASDSEVIEAAKLANAHNFISALPEGYSTKVGERGVQLSGGQRQRVAIARAVLKNPEILLLDEATSALDVESERVVQQALDRLMQNRTTIMVAHRLSTIRNADQISVLQDGKIIEQGTHSSLRENKNGAYFKLVNIQQQQHQL
51.	GgABCB40	MT379712	MVGQEPILFATSILDNVMMGKDNATKEEAIAACIAADAHNFISSLPQGYDTQVGDRGTKLSGGQKQRIALARAMIKDPKILLLDEPTSALDAESESTVQRAIDKISAGRTTIVIAHRIATVKNADAIVVLEHGSVTEIGDHRQLMAKAGTYYNLVKLATEAISKPLSKDSMQIIANNLSIYDKSAPDISRSRYLVDTSEPKILKSKQEEEQEEMEDKKNKKPRKYRLSDVWKLQKPELVMLFSGLLLGMFAGAFLSLFPLVLGISLGVYFNDDISKMKRDVGYLCLVLVGLGFGCILSMTGQQGLCGWAGSKLTLRVRNLLFQSILKQEPGWFDFEDNSTGVLVSRLSIDSVSFRSVLGDRFSVLLMGLSSAAVGLGVSFYFNWKLTLVAAAVTPLTLGASYISLIINIGPRVDNNSYARASNIASGAVSNIRTVTTFSAQEQIVKSFDRALSEPRKESLRSSQLQGLIFGIFQGAMYGAYTLALWFGAYLVKYDNANFEDVYKIFLILVLSSFSVGQLAGLAPDTSMAASAIPAVQDIIYRRPLIGNDRRESRKVERSKQFKIEFKMVTFAYPCRPEVTVLRDFCLKVKGGSTVALVGPSGSGKSTVIWMIQRFYDPDQGKVMMSGVDLREVDVKWLRRQIALVGQEPALFAGSIRENIAFGDPSASWAEIEAAAKEAYIHKFISGLPQGYETQVGESGVQLSGGQKQRIAIARAILKKSKVLLLDEASSALDLESEKHIQDALKNVSKDATTIIVAHRLSTIREADKIAVMRDGEVVEYGSHDTLISSLQNGLYASLVRAETEANAFA
52.	GgABCB41	MT379713	MVGQEPILFATSILDNVMMGKDNATKEEAIAACIAADAHNFISSLPQGYDTQVGDRGTKLSGGQKQRIALARAMIKDPKILLLDEPTSALDAESESTVQRAIDKISAGRTTIVIAHRIATVKNADAIVVLEHGSVTEIGDHRQLMAKAGTYYNLVKLATEAISKPLSKDSMQIIANNLSIYDKSAPDISRSRYLVDTSEPKILKSKQEEEQEEMEDKKNKKPRKYRLSDVWKLQKPELVMLFSGLLLGMFAGAFLSLFPLVLGISLGVYFNDDISKMKRDVGYLCLVLVGLGFGCILSMTGQQGLCGWAGSKLTLRVRNLLFQSILKQEPGWFDFEDNSTGVLVSRLSIDSVSFRSVLGDRFSVLLMGLSSAAVGLGVSFYFNWKLTLVAAAVTPLTLGASYISLIINIGPRVDNNSYARASNIASGAVSNIRTVTTFSAQEQIVKSFDRALSEPRKESLRSSQLQGLIFGIFQGAMYGAYTLALWFGAYLVKYDNANFEDVYKIFLILVLSSFSVGQLAGLAPDTSMAASAIPL
53.	GgABCB42	MT379714	MFYASCGNIVQKLGAGQVMLDGHDIKTLKLRWLRQQIGLVSQEPALFATTIRENILLGRPDANQVEIEEAARVANAHSFIIKLPDGYETQVGERGLQLSGGQKQRIAIARAMLKNPAILLLDEATSALDSESEKLVQEALDRFMIGRTTLVIAHRLSTIRKADLVAVLQQGSVSEIGTHDELFSKGESGAYAKLIKMQEMAHETAMSNARKSSARPSSARNSVSSPIITRNSSYGRSPYSRRLSDFSTSDFSLSLDASSYPNYRLEKLPFKEQASSFWRLAKMNSPEWLYALIGSIGSVVCGSLSAFFAYVLSAVLSVYYNPNHRHMIREIEKYCYLLIGLSSAALLFNTLQHFFWDIVGENLTKRVREKMLTAVLKNEMAWFDQEENESARIAARLALDANNVRSAIGDRISVIVQNTALMLVACTAGFVLQWRLALVLVAVFPVVVAATVLQKMFMTGFSGDLEAAHAKATQLAGEAIANVRTVAAFNSEKKIVGLFTSNLEIPLRRCFWKGQISGSGYGIAQFALYASYALGLWYASWLVKHGISDFSKTIRVFMVLMVSANGAAETLTLAPDFIKGGRAMKSVFDLLDRRTEIEPDDPDATPVPDRLRGEVELKHVDFSYPTRPDMPVFRDLSLRARAGKTLALVGPSGCGKSSVIALIQRFYDPTSGRVMIDGKDIRKYNLKSLRRHIAVVPQEPCLFASTIYENIAYGHDSATEAEIVEAATLANAHKFISSLPDGYKTFVGERGVQLSGGQKQRIAIARAFVRKAELMLLDEATSALDAESERSVQEALDRACSGKTTIIVAHRLSTIRNANVIAVIDDGKVAEQGSHSHLLKNHPDGIYARMIQLQRFTNSQVVGVAPGSSSSGRPKDDEREG
54.	GgABCB43	MT379715	MGLVSQEPALFATTIAGNILFGKEDANMDQIIQAAKAANAHSFITGLPEGYHTQVGEGGTQLSGGQKQRIAIARAVLRNPKILLLDEATSALDAESELIVQQALERIMSNRTTIIVAHRLSTIRDVDTIIVLKNGQVVESGTHLELMSKNGEYVNLVSLQSSHNLTSSSTISRSGSSRNSSFREPSDNLNHEEELNTRELQSSDRGLPSNTASIPSILDLLKLNAPEWPYAVLGSVGAVMAGMEAPLFALGITHILTAFYSPHGSQIKQEVDRVALIFVGVAVVTIPIYLLQHYFYSLMGERLTARVRLLMFSAILTNEVAWFDLDENNTGSLTAMLAADATLVRSALADRLSTIVQNVALTVTAFVIAFTLSWKLTLVVAACLPLLIGASITEQLFLKGFGGDYGHAYSRATSLAREAISNIRTVAAFGAEDRISIQFASELNKPNKQAFLRGHISGFGYGVTQLFAFCSYALGLWYASVLMKKKESNFGDLMKSFMILIITALAIAETLALTPDIVKGSQALGSVFSILHRRTAINPNDPSSKTITEVKGEIKFQNVCFKYPMRPDITIFQNLDLRVPAGKSLAVVGQSGSGKSTVIALVMRFYDPTSGSVLIDDCDIKSLNLRSLRLRIGLVQQEPALFSTTVYENIKYGKEEASEIEVMKAAKAANAHEFISRMPEGYKTEVGERGVQLSGGQKQRVAIARAILKDPSILLLDEATSALDTVSERLVQEALDKLMEGRTTILVAHRLSTVRAADGIAVLQHGRIAEMGSHERLMAKPGSIYKQLVSLQQEKRGQEDH
55.	GgABCB44	MT379716	MIMEKGYNGGTVINVIIAVLTASMSLGQASPSMSAFAAGQAAAYKMFQTIARKPEIDAYDPNGKILEDIQGEIKLRDVYFSYPARPEELIFNGFSLHIPSGSTAALVGQSGSGKSTVISLVERFYDPQGGEVLIDGINLKEFQLRWIRGKIGLVSQEPVLFASSIKANIAYGKDGATIEEIRSASELANAAKFIDKLPQGLDTMVGDHGTQLSGGQKQRIAIARAILKDPRILLLDEATSALDAESERVVQEALDRIMVNRTTVVVAHRLSTVRNADMIAVIHRGKMVEKGTHSELLKDPEGAYSQLIRLQEVNKESEETADHHNKGELSAESFRQSSQKRSLQRSISRGSSVGNSSRHSFSVSFGLPTGVNVPDPEHENLQPKEKGQEVPLRRLASLNKPEIPVLLIGCLAAIGNGVILPIFGVLISSVIKTFYEPFDEMKKDSKFWALMFMVLGLASLLVIPARGYFFAVAGCKLIQRIRLICFEKVVNMEVSWFDEPENSSGAIGARLSADAASVRALVGDALGLLVQNLASALAGLIIAFVASWQLSLIILVLIPLIGLNGFVQMKFMKGFSADAKMMYEEASQVANDAVGSIRTVASFCAEDKVMELYRTKCEGPMKTGIRQGLISGSGFGVSFFLLFCVYATSFYAGARLVDAGDATFSDVFRVFFALTMAAIGVSQSSSFAPDSSKAKSATASIFGIIDKKSKIDSSDESGTTLDSVKGEIELRHVSFKYPSRPDIQIFRDLNLAIHSGKVKFEFYFQTDPFHFKKQLKCIYVASCMSQLMVTNKIAVTDGGPCW
56.	GgABCB45	MT379717	MLPQGYDTQVGERGVQMSGGQKQRIAIARAIIKMPRILLLDEATSALDSESERVVQEALDKAAVGRTTIIIAHRLSTIRNADVIAVVQNGRVMETGSHESLIQNDNSLYTSLVHLQQTDKTQNDQNDTSSIITRDMQNINNTSSRRLSLSHVSRSSSSNSMARVVDDFNVNIDDVEDNNNNNKKQLAAPSFRRLLALNIPEWKQACLGCLNAVLFGAIQPVYAFAMGSVISVYFLDDHDEIKRQIRIYSLCFLGLALFSLVVNVIQHYNFAYMGEYLTKRVRERMLSKILTFEVGWFDQDENSTGAICSRLAKEANVVRSLVGDRMALVVQTISAVVIAFTMGLVVAWRLAIVMIAVQPIIIACFYTRRVLLKNMSSKAIKAQDESSKIAAEAVSNLRTITAFSSQDRILKMLEKAQEGPSRESLRQSWFAGIGLACSQSLTFCTWALDFWYGGRLVSHGYIKAKALFETFMILVSTGRVIADAGSMTNDLAKGADAVGSVFAILDRYTKIEPDDEIDGYKPEKITGKIELHDVHFAYPARPDVMIFQGFSIQIDAGKSTALVGQSGSGKSTIIGLIERFYDPHKGTVTIDGRDIRSYHLRSLRKRIALVSQEPTLFGGTIRENIAYGASSKLTDESEIIEAARAANAHDFIASLKDGYDTWCGDRGLQLSGGQKQRIAIARAILKNPEVLLLDEATSALDSQSEKLVQDALERVMVGRTSVVVAHRLSTIQNCDLIAVLDKGKVVEKGTHSSLLGKGTSGAYYSLVSLQRRPTNTINADSAHEIS

57.	GgABCC1	MT379718	MSVMLMMRALPASAIISCGDFSWICLKDFDFTSFCSQRSAINAINLLFVSVFYSSLLISLLFRKSSSNGRHRRSW<br>IFPVVSICCALISIAFFSIGLWNLIAKTGHDSKQQLSWLACIIRGFIWISFTVSLLVQRFKWIKILNSVWWASSCLF<br>VSALNIEILLKNHAIETFDIIEWLVNFLLLFCSFKSLDFSVTTSVPECLSEPLLTQKNVETKQTGLSRATFLSKLIF<br>SWVNSLLRVGYSRPLALEDIPSLVSEDEADKAYQNFIHAWESLVRERSKNNTKSLVLWSVVKTYLKENILIAF<br>YALLRTICVVVSPLLLYAFVNYSNSSTSTEPDLKQGLSIVGFLILTKLVESFSQRHWYFDSRRSGMKMRSALM<br>VAVYKKQLKISSSARRRHSAGEIVNYIAVDAYRMGEFPWWFHTTWCCALQLVLSIAILLGVVGIGALPGLVPL<br>LICGLLNVPFAKILQNCQSQFMVAQDERLRSTSEILNSMKIIKLQSWEEKFKNLVESLRAKEFIWLSKAQILKAS<br>NSFLYWMSPTVVSAVVFLGCALFKSAPLNAQTIFTVLATLRNMGEPVRMIPEALSIMIQVMVSFDRLNNFLLD<br>EELIQDDDGRNVKQGSVNAVEIQSGNFIWDHESLSPTLSDVNLEIKRGQKIAVCGPVGAGKSSLLYAILGEIPKI<br>SGTVSVGGTLAYVSQSSWIQSGTVRDDILFGKPMDKARYDNAIKVCALDKDINDFSHADLTEIGQRGINMSG<br>GQKQRIQLARAVYNDADIYLLDDPFSAVDAHTAAILFNDCVMTALRDKTVILVTHQVEFLSEVDTILVMEGG<br>KVIQSGSYENLLTAGTAFEQLVSAHKDAISEVNEDNNENKGGSENEQTHGFYLTKNQSEGEISISKGQLGVQL<br>TQEEGKEIGEVGWKPFWDYVTFSRGTFMLCLVMLAQSAFVALQTASTFWLAIAIEIPKVTSGTLIGVYSLISFV<br>SAAFVYLRSYLTALLGLKASTAFFSSFTTAIFNAPMLFFDSTPVGRILTRASSDLSILDFDIPYSITFVAAVAIEIL<br>VIICIMASVTWQVLIVAVPTMVASKYVQGYYIASARELVRINGTTKAPVMNFAAETSLGVVTVRAFNMVDRF<br>FKSYLKLVDTDATLFFHSNAAMEWIILRIEALQNLTVVTAALLLILLPQGYVSPGLVGLSLSYAFTLTSAQIFW<br>TRWFCNLSNYIISVERIKQFIHITPEPPAIVEDNRPPSSWPSKGRIDLQALEIRYRPNAPLVLKGITCTFKEGSRVG<br>VVGRTGSGKSTLISALFRLVEPSRGDVIIDGINICSIGKDLRIKLSIIPQEPTLFKGSIRTNLDPLGLYADDEIWKV<br>NEIASDTW
58.	GgABCC2	MT379719	MSVMLMMRALPASAIISCGDFSWICLKDFDFTSFCSQRSAINAINLLFVSVFYSSLLISLLFRKSSSNGRHRRSW<br>IFPVVSICCALISIAFFSIGLWNLIAKTGHDSKQQLSWLACIIRGFIWISFTVSLLVQRFKWIKILNSVWWASSCLF<br>VSALNIEILLKNHAIETFDIIEWLVNFLLLFCSFKSLDFSVTTSVPECLSEPLLTQKNVETKQTGLSRATFLSKLIF<br>SWVNSLLRVGYSRPLALEDIPSLVSEDEADKAYQNFIHAWESLVRERSKNNTKSLVLWSVVKTYLKENILIAF<br>YALLRTICVVVSPLLLYAFVNYSNSSTSTEPDLKQGLSIVGFLILTKLVESFSQRHWYFDSRRSGMKMRSALM<br>VAVYKKQLKISSSARRRHSAGEIVNYIAVDAYRMGEFPWWFHTTWCCALQLVLSIAILLGVVGIGALPGLVPL<br>LICGLLNVPFAKILQNCQSQFMVAQDERLRSTSEILNSMKIIKLQSWEEKFKNLVESLRAKEFIWLSKAQILKAS<br>NSFLYWMSPTVVSAVVFLGCALFKSAPLNAQTIFTVLATLRNMGEPVRMIPEALSIMIQVMVSFDRLNNFLLD<br>EELIQDDDGRNVKQGSVNAVEIQSGNFIWDHESLSPTLSDVNLEIKRGQKIAVCGPVGAGKSSLLYAILGEIPKI<br>SGTVSVGGTLAYVSQSSWIQSGTVRDDILFGKPMDKARYDNAIKVCALDKDINDFSHADLTEIGQRGINMSG<br>GQKQRIQLARAVYNDADIYLLDDPFSAVDAHTAAILFNDCVMTALRDKTVILVTHQVEFLSEVDTILVMEGG<br>KVIQSGSYENLLTAGTAFEQLVSAHKDAISEVNEDNNENKGGSENEQTHGFYLTKNQSEGEISISKGQLGVQL<br>TQEEGKEIGEVGWKPFWDYVTFSRGTFMLCLVMLAQSAFVALQTASTFWLAIAIEIPKVTSGTLIGVYSLISFV<br>SAAFVYLRSYLTALLGLKASTAFFSSFTTAIFNAPMLFFDSTPVGRILTRASSDLSILDFDIPYSITFVAAVAIEIL<br>VIICIMASVTWQVLIVAVPTMVASKYVQGYYIASARELVRINGTTKAPVMNFAAETSLGVVTVRAFNMVDRF<br>FKSYLKLVDTDATLFFHSNAAMEWIILRIEALQNLTVVTAALLLILLPQGYVSPGLVGLSLSYAFTLTSAQIFW<br>TRWFCNLSNYIISVERIKQFIHITPEPPAIVEDNRPPSSWPSKGRIDLQALEIRYRPNAPLVLKGITCTFKEGSRVG<br>VVGRTGSGKSTLISALFRLVEPSRGDVIIDGINICSIGLKDLRIKLSIIPQEPTLFKGSIRTNLDPLGLYADDEIWK<br>ALEKCQLKETISKLPSLLDSSVSDEGGNWSLGQRQLFCLGRVLLKRNRILVLDEATASIDSATDAILQKVIRQE<br>FAECTVITVAHRVPTVMDSDMVMVLSYGKLVEYDEPSKLMDTNSSFSKLVAEYWSSCRKNSFPNIRRQLQ
59.	GgABCC3	MT379720	MSVMLMMRALPASAIISCGDFSWICLKDFDFTSFCSQRSAINAINLLFVSVFYSSLLISLLFRKSSSNGRHRRSW<br>IFPVVSICCALISIAFFSIGLWNLIAKTGHDSKQQLSWLACIIRGFIWISFTVSLLVQRFKWIKILNSVWWASSCLF<br>VSALNIEILLKNHAIETFDIIEWLVNFLLLFCSFKSLDFSVTTSVPECLSEPLLTQKNVETKQTGLSRATFLSKLIF<br>SWVNSLLRVGYSRPLALEDIPSLVSEDEADKAYQNFIHAWESLVRERSKNNTKSLVLWSVVKTYLKENILIAF<br>YALLRTICVVVSPLLLYAFVNYSNSSTSTEPDLKQGLSIVGFLILTKLVESFSQRHWYFDSRRSGMKMRSALM<br>VAVYKKQLKISSSARRRHSAGEIVNYIAVDAYRMGEFPWWFHTTWCCALQLVLSIAILLGVVGIGALPGLVPL<br>LICGLLNVPFAKILQNCQSQFMVAQDERLRSTSEILNSMKIIKLQSWEEKFKNLVESLRAKEFIWLSKAQILKAS<br>NSFLYWMSPTVVSAVVFLGCALFKSAPLNAQTIFTVLATLRNMGEPVRMIPEALSIMIQVMVSFDRLNNFLLD<br>EELIQDDDGRNVKQGSVNAVEIQSGNFIWDHESLSPTLSDVNLEIKRGQKIAVCGPVGAGKSSLLYAILGEIPKI<br>SGTVSVGGTLAYVSQSSWIQSGTVRDDILFGKPMDKARYDNAIKVCALDKDINDFSHADLTEIGQRGINMSG<br>GQKQRIQLARAVYNDADIYLLDDPFSAVDAHTAAILFNDCVMTALRDKTVILVTHQVEFLSEVDTILVMEGG<br>KVIQSGSYENLLTAGTAFEQLVSAHKDAISEVNEDNNENKGGSENEQTHGFYLTKNQSEGEISISKGQLGVQL<br>TQEEGKEIGEVGWKPFWDYVTFSRGTFMLCLVMLAQSAFVALQTASTFWLAIAIEIPKVTSGTLIGVYSLISFV<br>SAAFVYLRSYLTALLGLKASTAFFSSFTTAIFNAPMLFFDSTPVGRILTRASSDLSILDFDIPYSITFVAAVAIEIL<br>VIICIMASVTWQVLIVAVPTMVASKYVQVYYPLFLDFTFSVPCKFIEL
60.	GgABCC4	MT379721	MSSVNVTWMTSLSCISTVKQTSGETFISTLPQWLRFIFLSPCPQRALFSAVNVLLLFIFFTFGLTKLYTKFTTSNG<br>GSPTELNKPLIGYNRSCVKTSVWFNLTLTATAVLAILYSVSCVLVFSSSNIESQWKLVDGTFWVVVQAITHAIVA<br>ILILHEKKHGTVAVSHHHPLSLRVYWVANFIVISLFSASGVIRLVSETENRDSMVFKVDDVASFISLPLSLFLLC<br>VAVKGSTGIVVSAEETQTLGVDNNNEVKDLQYHDDTTVTKSKSEVVTGFASASIVSKAFWIWINPLLSKGYK<br>SALKIDDVPMLSPEHRAEKISSLFESNWPKTNEKHPVRITLIRCFWKELAFTAFLAIVRLCVMFVGPILIQSFVD<br>FTSGKRSSVYEGYYLVLILLVAKFTEVMARSHASFNSQKIGTLIRCSLIPCLYKKGLMLSFSARQDHGVGTIVN<br>YMAVDIQQLSDMMLQLNAVWTMPFQIGIGLFLLYNCLGSSAVAAFLGLVLVFAVISTRKNNGFQFNVMK<br>NRDSRMKAVNEMLSYMRVIKFQAWEEHFNGRIMGFRGSEYSWLSKLMFSICGNFVVMWSSPLLISTLTFGVA<br>VLLGVRLDAATVFTATTIFKILQEPIRTFPQSMISLSQAMISLERLDRFMSSVELSNDSVEREEEGCGGGETAVE<br>VKDGIFSWDDDKQHKDLKNINLEIKKGELTAIVGTVGSGKSSLLASILGEMRKIYGKVRVCGSVAYVAQTSWI<br>QNGTIEENILFGLPMERQKYNEVIKACCLEKDLEMMDYGDQTEIGERGINLSGGQKQRIQLARAIYQDSDIYL<br>LDDIFSAVDAHTGSEIFKECVRGVLKDKTIILVTHQVDFLHNVDLILVMRDGMIVQSGKYNDLIDSGMDFKAL<br>VAAHETSMELVEQGAFIPGENLNKPAKSPETASIYKGTNGESNSPDKPESGNNKSSKLIKEEEKEIGKVSLHIY<br>KLYCTEAFGWWGITGVLILSLLWQGSLMASDYWLAYETSEERAQMFNPSLFVSVYAIITLVSVAFVVMRCYS<br>FTFLGLKTAQNFFTQILHSILHAPMSFFDTTPSGRILSRASTDQTNVDVTLPMFMGIAITMYITVLSILIITCQYS<br>WPTMLLIIPLVWLNIWYRGYFLASSRELTRLDSITKAPIIHHFSESIAGVMTIRAFRKQKRFCEENLKRVNANLR<br>MDFHNYSSNVWLGLRLELLGSFFFCISTMFMIILPSSIIKPENVGLSLSYGLSLNSVLFWCVFQSCIIENKLVSVE<br>RIKQFTNIPSEPAWKIKDHVPPSNWPGQGNVDIKDLQVRYRPNSPLVLKGITLSISGGEKIGVVGRTGSGKSTLI

			QVLFRLVEPSGGKVIVDSIDISVLGLHDLRSRFGIIPQEPVLFEGTVRSNIDPTGQYTDEEIWKSLERCQLKEVV<br>GAKPEKLDSLVVDNGENWSVGQRQLLCLGRVMLKRSRLLFMDEATASVDSQTDGVIQKIIREDFAACTIISIA<br>HRIPTVMDCDRVLVVDAGRVKEFDKPSNLLQRPSLFGALVQEYASRSTGL
61.	GgABCC5	MT379722	MSSVNVTWMTSLSCISTVKQTSGETFISTLPQWLRFIFLSPCPQRALFSAVNVLLLFIFFTFGLTKLYTKFTTSNG<br>GSPTELNKPLIGYNRSCVKTSVWFNLTLTATAVLAILYSVSCVLVFSSSNIESQWKLVDGTFWVVQAITHAIVA<br>ILILHEKKHGTVAVSHHHPLSLRVYWVANFIVISLFSASGVIRLVSETENRDSMVFKVDDVASFISLPLSLFLLC<br>VAVKGSTGIVVSAEETQTLGVDNNNEVKDLQYHDDTTVTKSKSEVVTGFASASIVSKAFWIWINPLLSKGYK<br>SALKIDDVPMLSPEHRAEKISSLFESNWPKTNEKHPVRITLIRCFWKELAFTAFLAIVRLCVMFVGPILIQSFVD<br>FTSGKRSSVYEGYYLVLILLVAKFTEVMARSHASFNSQKIGTLIRCSLIPCLYKKGLMLSFSARQDHGVGTIVN<br>YMAVDIQQLSDMMLQLNAVWTMPFQIGIGLFLLYNCLGSSAVAAFLGLLGVLVFAVISTRKNNGFQFNVMK<br>NRDSRMKAVNEMLSYMRVIKFQAWEEHFNGRIMGFRGSEYSWLSKLMFSICGNFVVMWSSPLLISTLTFGVA<br>VLLGVRLDAATVFTATTIFKILQEPIRTFPQSMISLSQAMISLERLDRFMSSVELSNDSVEREEEGCGGGETAVE<br>VKDGIFSWDDDKQHKDLKNINLEIKKGELTAIVGTVGSGKSSLLASILGEMRKIYGKVRVCGSVAYVAQTSWI<br>QNGTIEENILFGLPMERQKYNEVIKACCLEKDLEMMDYGDQTEIGERGINLSGGQKQRIQLARAIYQDSDIYL<br>LDDIFSAVDAHTGSEIFKECVRGVLKDKTIILVTHQVDFLHNVDLILVMRDGMIVQSGKYNDLIDSGMDFKAL<br>VAAHETSMELVEQGAFIPGENLNKPAKSPETASIYKGTNGESNSPDKPESGNNKSSKLIKEEEKEIGKVSLHIY<br>KLYCTEAFGWWGITGVLILSLLWQGSLMASDYWLAYETSEERAQMFNPSLFVSVYAIITLVSVAFVVMRCYS<br>FTFLGLKTAQNFFTQILHSILHAPMSFFDTTPSGRILSRASTDQTNVDVTLPMFMGIAITMYITVLSILIITCQYS<br>WPTMLLIIPLVWLNIWYRVCI
62.	GgABCC6	MT379723	MSSVNVTWMTSLSCISTVKQTSGETFISTLPQWLRFIFLSPCPQRALFSAVNVLLLFIFFTFGLTKLYTKFTTSNG<br>GSPTELNKPLIGYNRSCVKTSVWFNLTLTATAVLAILYSVSCVLVFSSSNIESQWKLVDGTFWVVQAITHAIVA<br>ILILHEKKHGTVAVSHHHPLSLRVYWVANFIVISLFSASGVIRLVSETENRDSMVFKVDDVASFISLPLSLFLLC<br>VAVKGSTGIVVSAEETQTLGVDNNNEVKDLQYHDDTTVTKSKSEVVTGFASASIVSKAFWIWINPLLSKGYK<br>SALKIDDVPMLSPEHRAEKISSLFESNWPKTNEKHPVRITLIRCFWKELAFTAFLAIVRLCVMFVGPILIQSFVD<br>FTSGKRSSVYEGYYLVLILLVAKFTEVMARSHASFNSQKIGTLIRCSLIPCLYKKGLMLSFSARQDHGVGTIVN<br>YMAVDIQQLSDMMLQLNAVWTMPFQIGIGLFLLYNCLGSSAVAAFLGLLGVLVFAVISTRKNNGFQFNVMK<br>NRDSRMKAVNEMLSYMRVIKFQAWEEHFNGRIMGFRGSEYSWLSKLMFSICGNFVVMWSSPLLISTLTFGVA<br>VLLGVRLDAATVFTATTIFKILQEPIRTFPQSMISLSQAMISLERLDRFMSSVELSNDSVEREEEGCGGGETAVE<br>VKDGIFSWDDDKQHKDLKNINLEIKKGELTAIVGTVGSGKSSLLASILGEMRKIYGKVRVCGSVAYVAQTSWI<br>QNGTIEENILFGLPMERQKYNEVIKACCLEKDLEMMDYGDQTEIGERGINLSGGQKQRIQLARAIYQDSDIYL<br>LDDIFSAVDAHTGSEIFKECVRGVLKDKTIILVTHQVDFLHNVDLILVMRDGMIVQSGKYNDLIDSGMDFKAL<br>VAAHETSMELVEQGAFIPGENLNKPAKSPETASIYKGTNGESNSPDKPESGNNKSSKLIKEEEKEIGKVSLHIY<br>KLYCTEAFGWWGITGVLILSLLWQGSLMASDYWLAYETSEERAQMFNPSLFVSVYAIITLVSVAFVVMRCYS<br>FTFLGLKTAQNFFTQILHSILHAPMSFFDTTPSGRILSRASTDQTNVDVTLPMFMGIAITMYITVLSILIITCQYS<br>WPTMLLIIPLVWLNIWYRGYFLASSRELTRLDSITKAPIIHHFSESIAGVMTIRAFRKQKRFCEENLKRVNANLR<br>MDFHNYSSNVWLGLRLELLGSFFFCISTMFMIILPSSIIKPENVGLSLSYGLSLNSVLFWCVFQSCIIENKLVSVE<br>RIKQFTNIPSEPAWKIKDHVPPSNWPGQGNVDIKDLQVRYRPNSPLVLKGITLSISGGEKIGVVGRTGSGKSTLI<br>QVLFRLVEPSGGKVIVDSIDISVLGLHDLRSRFGIIPQEPVLFEGTVRSNIDPTGQYTDEEIWKSLERCQLKEVV<br>GAKPEKLDSLVVDNGENWSVGQRQLLCLGRVMLKRSRLLFMDEATASVDSQTDGVIQKIIREDFAACTIISIA<br>HRIPTVMDCDRVLVVDAGIFTNG
63.	GgABCC7	MT379724	MSSVNVTWMTSLSCISTVKQTSGETFISTLPQWLRFIFLSPCPQRALFSAVNVLLLFIFFTFGLTKLYTKFTTSNG<br>GSPTELNKPLIGYNRSCVKTSVWFNLTLTATAVLAILYSVSCVLVFSSSNIESQWKLVDGTFWVVQAITHAIVA<br>ILILHEKKHGTVAVSHHHPLSLRVYWVANFIVISLFSASGVIRLVSETENRDSMVFKVDDVASFISLPLSLFLLC<br>VAVKGSTGIVVSAEETQTLGVDNNNEVKDLQYHDDTTVTKSKSEVVTGFASASIVSKAFWIWINPLLSKGYK<br>SALKIDDVPMLSPEHRAEKISSLFESNWPKTNEKHPVRITLIRCFWKELAFTAFLAIVRLCVMFVGPILIQSFVD<br>FTSGKRSSVYEGYYLVLILLVAKFTEVMARSHASFNSQKIGTLIRCSLIPCLYKKGLMLSFSARQDHGVGTIVN<br>YMAVDIQQLSDMMLQLNAVWTMPFQIGIGLFLLYNCLGSSAVAAFLGLLGVLVFAVISTRKNNGFQFNVMK<br>NRDSRMKAVNEMLSYMRVIKFQAWEEHFNGRIMGFRGSEYSWLSKLMFSICGNFVVMWSSPLLISTLTFGVA<br>VLLGVRLDAATVFTATTIFKILQEPIRTFPQSMISLSQAMISLERLDRFMSSVELSNDSVEREEEGCGGGETAVE<br>VKDGIFSWDDDKQHKDLKNINLEIKKGELTAIVGTVGSGKSSLLASILGEMRKIYGKVRVCGSVAYVAQTSWI<br>QNGTIEENILFGLPMERQKYNEVIKACCLEKDLEMMDYGDQTEIGERGINLSGGQKQRIQLARAIYQDSDIYL<br>LDDIFSAVDAHTGSEIFKVVISYSQHSIHFLVDSSW
64.	GgABCC8	MT379725	MAYFWSSLGGLSWICGGEFNLASFCIQRSIIDGVNIFFFCVFCLFMLIGLVRKHPSNDVHRKDRVLVVTSICCFL<br>TCMVYFGYGLYNFIAQNGKLDNLSWVSFTIKGIIWASLAVSLLTQRSKRIICLNSIWWVCLCALLSALNIEILLI<br>VHSIPVFDLLPWLVSFLLLLCALRNHGYFISKHFQYNTMFEPLLCETEKAETNQPGLSQANFFSKLTFSWMNPL<br>LSLGYTKPLVLENIPSLPPEDKANTCYQKFASTWDSLLRGSSSNSTKNLVIWAMSRAFLKENIYIAIFAFARSIC<br>AAASPLIVYAFVNYASHNEENLYEGLSLLGCLVLIKLVETVSERQWNFDSWRSGMRMRSALMVAVYEKLLK<br>LSSLGRKKHSTGEVVNYIAVDAYRMGEFLYWFHTAWSFVLQLFLAIGVLLWVVGLGALPGLVLLLIFGVFNV<br>PYAKKIRTCKSQVLASQDQRLRSTSEILNNIKIIKLQSWEDKFKDMVESLRASEIKWLAEAQFTRALGSLLYAS<br>TTIIGAVVLIGCTLFGTAPLNAGTIFTVLATLRSMAEPVRFIPEAISAIIQVKVSLDRLNIFLLDDELKTSQKRSTY<br>VSKSDKCIEIEAANFTWDEESVTPTLRHINLGIKCGQKVAVCGPVGAGKSSLLHAILGEMPKISGTVNLHGAV<br>AYVSQTSWIQSGTIEDNILFGKPMERNRYENAIKACALDKDINGFSHGDLTEIGQRGVNLSGGQKQRVQLARA<br>VYNDADIYLLDDPFSAVDAHTAAILFHDCVMSALKEKTVILVTHQVEFLTEVDKILVLEGGVITQAGSHEELL<br>TSGTTFEQLMNAHRDAITVMGASHQDQEKCQEVGRVDNEQGHHGCDPTNKNSNEEICETGFAGQQLTPEEH<br>TEMGNAGWELFLDYIIISKGLLLQFLSFIALIGSAAFLAAASYWLAIASEIPSIADLMLVGVYTALSLLSAAFIYI<br>RSLLVAHHGLKASKAFFSSFTSAIFNAPMSFFDSTPVGRILTRVSSDFATLDIDLPFAIVYVAQSGTELLVGILIM<br>SSVTWQVLIVAILIAVAGYYIKGYYQASARELVRINGITKAPVVNCTTETSAGVVTVRAFKMVDRFFITFLHLV<br>DTDAALFLHTNAAMEWLQSRIEILQNLVFFAAASLFVFLPMGSIAPGLVGLSLSYALSLTRSQIYFTKWSCSLS<br>NFIISVERIMQFMQIPQEPPKILEDKRPPSSWPSKGRIEFQALKVRYRPNAPLVLNGITCTFKEGTRVGVVGRTG<br>SGKTTLLSALFRLVEPTSGEVLIDGLNICSIGLKDLRMKLSIIPQEPILFRGSVRTNLDPLDQFSDDEIWKVLEMC<br>QLKEVLCGLPYLLDSSVSNEGENWSVGQRQLFCLGRVLLRRNRILVLDEATASIDSATDAILQRVIRQEFSECT<br>VVTVAHRVPTVIDSDMVMVLSYGKVVEFDEPSKLMEDKSSSFSKLVAEYWSSCMRNSF

65.	GgABCC9	MT379726	MVVQLFLFSAQLMSDGVILQAAPYHHLLTSSQEFQDLVNAHKETAGSDQLVNVTLPPRHSTSRKTMQASLEKQFKAENGNQLIKQEERERGDTGLKPYLQYLNQKNGYVFFFVASLSHLVFVVCQILQNSWMAANVDNPHVSKLKLIVVYFLIGAFSTIFLLTRSMFVVALGLQSSKYLFLQLMNSLFRAPMSFYDSTPLGRILSRVSSDLSIMDLDIPFILTYAVGGTINCYSNLTVLAVVTWQVLIVSLPMIYVAIRLQRYYFASAKEVMRMNGTTKSFVANHIAETVAGAVTIRAFEEEDRFFEKNLDLIDINASAFFHNFASNEWLIQRLETISAVVLVSAALCMVMLPPGTFTSGFIGMALSYGLSLNASLVFSIQSQCTLANYIISVERLNQYMHIQSEAQEVIEGNRPPLNWPVAGKVEIKDLKIRYRPDEPLVLHGITCTFKAGHKIGIVGRTGSGKSTLIGALFRLVEPAGGKIVVDGIDILSIGLHDLRSRFGVIPQDPTLFNGTVRFNLDPLSQHTDQEIWEVLGKCQLREVVQEKEEGLNSSVVEDGSNWSMGQRQLFCLGRALLRRSRILVLDEATASIDNATDLILQKTIRTEFVDCTVITVAHRIPTVMDCTMVLSISDGKLAEYDEPRSLMKREESLFRQLVKEYWSHFQSAESY
66.	GgABCC10	MT379727	MVGFWSVFCGESGCSEAGRKPCSYDFKLLIDPSACINHLLISCFDVLLLIMLLFIMIQKSSLKGPFQGHIRGQRYSNLQLVSAIANGALGLVHLFLGIWILEENLRKTQTALPLDLWLLEFFQGLTWLLVGLTLSLKLKQLPRTWLRLSSILIFLVSGIFCALSLFYAISSGELSLKVALDVLSFSGAILLVLCTYKESRRRETDREIDESLYTPLNGESNKNDSVIQVTRFAKAGFFSRMSFWWLNPLMKRGKEKTLNDKDVPKLREEDRAESCYSLFLDRLSKQKQKDPSSQPSVLRTLILCHRREILISGFFALLKVLALSSGPLLLNSFILVAEGYESFKYEGFVLAISLFFTKIIESLSQRQWYFRSRLIGLKVRSLLTAAIYRKQLRLSNSARLMHSGGEIMNYVTVDAYRIGEFPYWFHQTWTTSFQLCISLVILFRAVGLATIASLVVILITVLCNTPLAKLQHKFQSKLMVAQDERLKATSEALVNMKVLKLYAWETNFKNAIEGLRNVELKWLSAVQLRKAYNTFLFWSSPVLVSAASFGACYFLNVPLHANNVFTFVATLRLVQDPIRTIPDVIGVVIQAKVALTRIVKFLEAPELQNANVRKRFSNDNMRGSISIKSANFSWEDSDVSKPTLRNINLEVRPGQKVAICGEVGSGKSTLLAAILREVPNTQGTIDVYGKFAYVSQTAWIQTGTIRENILFGSAMDAQKYEETLHRSSLLKDLELFPHGDLTEIGERGLNLSGGQKQRIQLARALYQNADIYLLDDPFSAVDAQTATNLFNEYIVEGLAGKTVLLVTHQVDFLPAFDSVLLMSDGEILHDAPYHHLLSTSQEFQDLVNAHKETAGSDRLVDVTSCQRYSSSAREIKKTYGEKEKQFEESKGDQLIKQEEREIGDQGFKPYLQYLNQNKGYVYFSVASLSHLVFVIGQILQNSWMAANVDNPQVSTLRLILVYLLIGVTSTLFLLMRSLLTVALGLQSSKSLFLQLLNTLFRAPMSFYDSTPLGRILSRVSSDLSIVDLDVPFGLLFAVVATTNCYANLTVLAVVTWQVLFVSIPMIYLALRLQKYYFASAKELMRMNGTTKSFVANHLAESVAGAVTIRAFEEEDRFFVKNLDLIDINASAFFHNFASNEWLIQRLETISAVVLVSAALCMVMLPPGTFTSGFIGMALSYGLSLNASLVFSIQSQCTLANYIISVERLNQYMHIQSEAQEVIEGNRPPLNWPVAGKVEIKDLKIRYRPDEPLVLHGITCTFKAGHKIGIVGRTGSGKSTLIGALFRLVEPAGGKIVVDGIDILSIGLHDLRSRFGVIPQDPTLFNGTVRFNLDPLSQHTDQEIWEVLGKCQLREVVQEKEEGLNSSVVEDGSNWSMGQRQLFCLGRALLRRSRILVLDEATASIDNATDLILQKTIRTEFVDCTVITVAHRIPTVMDCTMVLSISDGKLAEYDEPRSLMKREESLFRQLVKEYWSHFQSAESY
67.	GgABCC11	MT379728	MEDIWSMICGEYSGCSETGGKPYDFKFLKDIPSTCISHVFIIFFDVLMLIMLSFIMILKSWSRPFWSLVRYSKLQLVSAITNGSLGLFHLCLGIWVSEEMLRKTHKAFPLNFWLLELFQGFTWLLVSLTVSLQIKQLPRAWLWLFSILMFFVSFILCALSMSYATGSRELSLNAALDVLSFLGASLLLLCTYKACKCAEADREIGESLYAPLNSQFNEADPVSNRTTPFAKAGFFSKMWFWWLNPLMKRGQEKTLQDEDIPKLRNSDRAESCYLSFAEPFNRQKQNEASSHSSVLWTIILCHRREILVTGFFALLKVLTISSGPLLLNAFILVAEGNESFKYEGYVLAISLFFVKIIESLSQRQWYFHSRLVGMKVRSLLTAAIYKKALRLSNSARLVHSGGEIMNYVTVDAYRIGEFPFWFHQTWTTILQLCIALVILFHAVGLATIASLVVIVITVLCNTPLAKLQHKFQSQLMVAQDERLKASSEALVNMKVLKLYAWETHFRNAIENLRNVELKVLSSLQSKKAYNIFLFWTSPMLVSAASFLACYFLEIPLHASNVFTFVATLRLVQDPITAIPDVVGVIIQAKVAFARIVKFLEAPELQNSNFRKRGISDNLRGSIFIKSADFSWEGSGSKPTLRSINLEVRHGQKVAICGEVGSGKSTLLTTILGEVPNTKGTIEVCGTFAYVSQTAWIQTGTIRENILFGADLDDQRYQETLQRSSLMKDIELFPNGDLTEIGERGVNLSGGQKQRIQLARALYQNADLYLLDDPFSAVDAHTAKNLFNEYIMEGLKGKTVLLVTHQVDFLPAFDSVLLMSDGVILQAAPYHHLLTSSQEFQDLVNAHKETAGSDQLVNVTLPQRHSTSRKTMQASLEKQFKAENGNQLIKQEERERGDTGLKPYLQYLNQKNGYVFFFVASLSHLVFVVCQILQNSWMAANVDNPHVSKLKLIVVYFLIGAFSTIFLLTRSMFVVALGLQSSKYLFLQLMNSLFRAPMSFYDSTPLGRILSRVSSDLSIMDLDIPFILTYAVGGTINCYSNLTVLAVVTWQVLIVSLPMIYVAIRLQRYYFASAKEVMRMNGTTKSFVANHIAETVAGAVTIRAFEEEDRFFEKNLDLIDINASAFFHNFASNEWLIQRLETISAVVLVSAALCMVMLPPGTFTSGFIGMALSYGLSLNASLVFSIQSQCTLANYIISVERLNQYMHIQSEAQEVIEGNRPPLNWPVAGKVEIKDLKIRYRPDEPLVLHGITCTFKAGHKIGIVGRTGSGKSTLIGALFRLVEPAGGKIVVDGIDILSIGLHDLRSRFGVIPQDPTLFNGTVRFNLDPLSQHTDQEIWEVLGKCQLREVVQEKEEGLNSSVVEDGSNWSMGQRQLFCLGRALLRRSRILVLDEATASIDNATDLILQKTIRTEFVDCTVITVAHRIPTVMDCTMVLSISDGKLAEYDEPRSLMKREESLFRQLVKEYWSHFQSAESY
68.	GgABCC12	MT379729	MICLFIPCQIRYRPDEPLVLHGITCTFKAGHKIGIVGRTGSGKSTLIGALFRLVEPAGGKIVVDGIDILSIGLHDLRSRFGVIPQDPTLFNGTVRFNLDPLSQHTDQEIWEVLGKCQLREVVQEKEEGLNSSVVEDGSNWSMGQRQLFCLGRALLRRSRILVLDEATASIDNATDLILQKTIRTEFVDCTVITVAHRIPTVMDCTMVLSISDGKLAEYDEPRSLMKREESLFRQLVKEYWSHFQSAESY
69.	GgABCC13	MT379730	MDNNLIKLILLLFELYTMYHHDFDTTSRLTLTELRSLSWTTWQPLQSPCLLENVILPVELGFFVILLVQLLRDCVNRIGSKQNKVSDRAPDMHPTAIKFGFAYKFSLVCTTLLLAVHALMLSLMLKHETQCTSRLEAFTSETIQVLSWAISLIAICKMSKSNTHFPWILRAWWLCSFLLSITSTALHAHFSVSNKGQLGIREYADFLGLLASTCLLVLSTRGKTGIVLIASNGMSEPLLGEKTEKHSDCQRESPYGKATLLQLINFSWLNPLFAVGYKKPLQQNDIPDVDIKDSAEFITCSFDESLRQVKEKDGTTNPSIYKAIYLFARKKAAINALFAIVSASASYVGPYLITDFVNFLGEKGIRGLKTGYLLSLAFLCAKMVETIAQRQWIFGARQLGLRLRAALITHIYKKGLHLSSISRQSHTGGEIMNYMSVDVQRITDFIWYVNVIWMLPIQISLAVLILHTNLGLGSMAALAATLAVMTLNIPLTKIQKRYQTKIMEAKDNRMKATSEVLKNMRTLKLQAWDSQFFQRIEALRQIEYDWLLKSLRQAAFSAFIFWGSPTFISVITFWACMFMGIELTAARVLSAFATFRMLQDPIFSLPDLLNVIAQGKVSVDRIASFLQEEEIQHDVIEYVAKDKTEFDIAIEKGRFSWDPDSTSPTIDDIELKIKRGMKVAICGSVGSGKSSVLSGILGEIYKQSGSVKISGTKAYVPQSAWILTGNIRDNITFGKEYNEDKYEKTIEACALKKDFELFSCGDMTEIGERGINMSGGQKQRIQIARAVYQDADIYLFDDPFSAVDAHTGTHLFKVRFSFILHICKYFMV
70.	GgABCC14	MT379731	MPLENLYNIFDATKLRSLSWTTWQPLQSPCLLENVILPVELGFFVILLVQLLRDCVNRIGSKQNKVSDRAPDMHPTAIKFGFAYKFSLVCTTLLLAVHALMLSLMLKHETQCTSRLEAFTSETIQVLSWAISLIAICKMSKSNTHFPWILRAWWLCSFLLSITSTALHAHFSVSNKGQLGIREYADFLGLLASTCLLVLSTRGKTGIVLIASNGMSEPLLGEKTEKHSDCQRESPYGKATLLQLINFSWLNPLFAVGYKKPLQQNDIPDVDIKDSAEFITCSFDESLRQVKEKDG

			TTNPSIYKAIYLFARKKAAINALFAIVSASASYVGPYLITDFVNFLGEKGIRGLKTGYLLSLAFLCAKMVETIAQ<br>RQWIFGARQLGLRLRAALITHIYKKGLHLSSISRQSHTGGEIMNYMSVDVQRITDFIWYVNVIWMLPIQISLAV<br>LILHTNLGLGSMAALAATLAVMTLNIPLTKIQKRYQTKIMEAKDNRMKATSEVLKNMRTLKLQAWDSQFFQ<br>RIEALRQIEYDWLLKSLRQAAFSAFIFWGSPTFISVITFWACMFMGIELTAARVLSAFATFRMLQDPIFSLPDLL<br>NVIAQGKVSVDRIASFLQEEEIQHDVIEYVAKDKTEFDIAIEKGRFSWDPDSTSPTIDDIELKIKRGMKVAICGS<br>VGSGKSSVLSGILGEIYKQSGSVKISGTKAYVPQSAWILTGNIRDNITFGKEYNEDKYEKTIEACALKKDFELFS<br>CGDMTEIGERGINMSGGQKQRIQIARAVYQDADIYLFDDPFSAVDAHTGTHLFKVRFSFILHICKYFMV
71.	GgABCC15	MT379732	MPLENLYNIFDATKLRSLSWTTWQPLQSPCLLENVILPVELGFFVILLVQLLRDCVNRIGSKQNKVSDRAPDM<br>HPTAIKFGFAYKFSLVCTTLLLAVHALMLSLMLKHETQCTSRLEAFTSETIQVLSWAISLIAICKMSKSNTHFP<br>WILRAWWLCSFLLSITSTALHAHFSVSNKGQLGIREYADFLGLLASTCLLVLSTRGKTGIVLIASNGMSEPLLG<br>EKTEKHSDCQRESPYGKATLLQLINFSWLNPLFAVGYKKPLQQNDIPDVDIKDSAEFITCSFDESLRQVKEKDG<br>TTNPSIYKAIYLFARKKAAINALFAIVSASASYVGPYLITDFVNFLGEKGIRGLKTGYLLSLAFLCAKMVETIAQ<br>RQWIFGARQLGLRLRAALITHIYKKGLHLSSISRQSHTGGEIMNYMSVDVQRITDFIWYVNVIWMLPIQISLAV<br>LILHTNLGLGSMAALAATLAVMTLNIPLTKIQKRYQTKIMEAKDNRMKATSEVLKNMRTLKLQAWDSQFFQ<br>RIEALRQIEYDWLLKSLRQAAFSAFIFWGSPTFISVITFWACMFMGIELTAARVLSAFATFRMLQDPIFSLPDLL<br>NVIAQGKVSVDRIASFLQEEEIQHDVIEYVAKDKTEFDIAIEKGRFSWDPDSTSPTIDDIELKIKRGMKVAICGS<br>VGSGKSSVLSGILGEIYKQSGSVKISGTKAYVPQSAWILTGNIRDNITFGKEYNEDKYEKTIEACALKKDFELFS<br>CGDMTEIGERGINMSGGQKQRIQIARAVYQDADIYLFDDPFSAVDAHTGTHLFKECLMGILKEKTILFVTHQV<br>EFLPAADLILVMQNGRIAQAGTFEELLKQNIGFEVLVGAHSKALESVLMVENSSRTNLNPIAEGESNTNSNSSV<br>NLEHTQHDTVQDNPPDGKESDGKLVQEEERETGSISKEVYWCYLTTVKGGILVPIIILAQSSFQILQIASNYWM<br>AWVCPTSSDAKPIFDMNFILLIYMVLSVTGSLCVLLRAMLVLNAGLWTAQTLFTRMLHNVLRAPMAFFDSTP<br>TGRILNRASTDQSVLDMEMANKIGWCAFSVIQILGTVAVMSQVAWQVFVIFIPVTGVCIWYQRYYTPTAREL<br>ARLAQIQITPILHHFSESLAGAASIRAFDQERRFVNTNLVLVDGFSKPWFHNVSAMEWLSFRLNLLSNFVFAFS<br>LVLLVSLPEGIINPSIAGLAVTYGINLNVLQAAVIWNICNAENKMISVERILQYTNIASEAPLVIEGCRPPSNWPE<br>TGTICFKNLQIRYAEQLPSVLKNITCTFPGRKKIGVVGRTGSGKSTLIQAIFRMVEPREGSIIIDNVDICKIGLHDL<br>RSRLSIIPQDPSLFEGTVRGNLDPLEQYSDTEVWEALDKCQLGHLIRAKEEKLDSQVVENGDNWSVGQRQLFC<br>LGRALLKKSSILVLDEATASVDSATDGVIQDIISHEFKDRTVVTIAHRIHTVIDSDLVLVLSDGRIAEYDEPSKLL<br>EREDSFFFKLIKEYSSRSRSFNSLATQHVQNRE
72.	GgABCC16	MT379733	MEWLSFRLNLLSNFVFAFSLVLLVSLPEGIINPSIAGLAVTYGINLNVLQAAVIWNICNAENKMISVERILQYTN<br>IASEAPLVIEGCRPPSNWPETGTICFKNLQIRYAEQLPSVLKNITCTFPGRKKIGVVGRTGSGKSTLIQAIFRMVE<br>PREGSIIIDNVDICKIGLHDLRSRLSIIPQDPSLFEGTVRGNLDPLEQYSDTEVWEALDKCQLGHLIRAKEEKLDS<br>QVVENGDNWSVGQRQLFCLGRALLKKSSILVLDEATASVDSATDGVIQDIISHEFKDRTVVTIAHRIHTVIDSD<br>LVLVLSDGRIAEYDEPSKLLEREDSFFFKLIKEYSSRSRSFNSLATQHVQNRE
73.	GgABCC17	MT379734	MEWLSFRLNLLSNFVFAFSLVLLVSLPEGIINPSIAGLAVTYGINLNVLQAAVIWNICNAENKMISVERILQYTN<br>IASEAPLVIEGCRPPSNWPETGTICFKNLQIRYAEQLPSVLKNITCTFPGRKKIGVVGRTGSGKSTLIQAIFRMVE<br>PREGSIIIDNVDICKIGLHDLRSRLSIIPQDPSLFEGTVRGNLDPLEQYSDTEVWEALDKCQLGHLIRAKEEKLDS<br>QVVENGDNWSVGQRQLFCLGRALLKKSSILVLDEATASVDSATDGVIQDIISHEFKDRTVVTIAHRIHTVIDSD<br>LVLVLSDGKCFRAQSELILVHLI
74.	GgABCC18	MT379735	MSTWITSLSCSSPGDRTSTTTLPQWLRFVFLSPCPQRALLSAVDVLLLLTLFVFALIKLYSRFTSNRSTPNSELN<br>KPLIGNTRASVRTTLWFKLTLTATVVLTIMYTVACILVFTSSTQVSWKQVDGLFWLLQAITQLVLAILIIHEKR<br>FEAVSHPISLRIYWVASFVIVSLFTASGVIRFVSVDIREGKYFSFLVDDTVSFLTLPKSLFLLCVAIKGSTGIIKSS<br>EETQPLVVEGETKLYDPITLTKSNVTGFASASAVSKAFWIWLNPLLSKGYKSPLNIDEVPLLSPQHRAERMSVI<br>FESKWPKSDEKSKHPVRTTLLRCFWKEIAFTAFLAVVRLSVMFVGPVLIQDFVDFTSGKGSSIYEGYYLVLILL<br>VAKFVEVLTTHHFNFNSQKLGMLIRCTLITSLYKKGLRLSCSARQDHGVGPIVNYMAVDTQQLSDMMLQLH<br>AVWMMPFQVGIGLFLLYNCLGVSVVTALLGLLGVLAFIVSATRKNKRYQFSAMMNRDSRMKAVNEMLNY<br>MRVIKFQAWEDHFNDRILAFRASEFGWLSKFLYSICGNIIVLWSTPLLISTLTFGTAIVLGVPLDAGTVFTTTTV<br>FKILQEPLRTFPQSMISLSQALVSLGRLDRYMSSKELSNDSVEREEGCDGHIAVEVKDGSFSWDDDGQEQDLK<br>NINLEINKGELTAIVGTVGSGKSSLLASILGEMHRISGKVQVCGTTAYVAQTSWIQSGTIEENILFGLPMNRQK<br>YNEVVRVCCLEKDLEMMEYGDLTEIGERGINLSGGQKQRIQLARAVYQDSDVYLLDDVFSAVDAHTGTEIFK<br>ECVRGALKGKTIILVTHQVDFLHNVDLIVVMRDGMIAQSGKYNDLLDSGMGFSALVAAHETSMELVEQGAA<br>MPGGEHLNRSIKSPREAPNNREANGESNSLDQPKSGEENSKLVKEEERETGKVSLHIYKLYCTEAFGWWGITG<br>VILLSLLWQASMMASDYWLAYETSEERAQLFNPSVFISIYAIIAAVSIVLIVLRSYAVTILGLKTAQIFFSQILSSI<br>LHAPMSFFDTTPSGRILSRASTDQTNVDIFIPLFLNFVVAMYITVISIFIITCQNSWPTAFLLIPLLWLNIWYRVW<br>TWFLC
75.	GgABCC19	MT379736	MSTWITSLSCSSPGDRTSTTTLPQWLRFVFLSPCPQRALLSAVDVLLLLTLFVFALIKLYSRFTSNRSTPNSELN<br>KPLIGNTRASVRTTLWFKLTLTATVVLTIMYTVACILVFTSSTQVSWKQVDGLFWLLQAITQLVLAILIIHEKR<br>FEAVSHPISLRIYWVASFVIVSLFTASGVIRFVSVDIREGKYFSFLVDDTVSFLTLPKSLFLLCVAIKGSTGIIKSS<br>EETQPLVVEGETKLYDPITLTKSNVTGFASASAVSKAFWIWLNPLLSKGYKSPLNIDEVPLLSPQHRAERMSVI<br>FESKWPKSDEKSKHPVRTTLLRCFWKEIAFTAFLAVVRLSVMFVGPVLIQDFVDFTSGKGSSIYEGYYLVLILL<br>VAKFVEVLTTHHFNFNSQKLGMLIRCTLITSLYKKGLRLSCSARQDHGVGPIVNYMAVDTQQLSDMMLQLH<br>AVWMMPFQVGIGLFLLYNCLGVSVVTALLGLLGVLAFIVSATRKNKRYQFSAMMNRDSRMKAVNEMLNY<br>MRVIKFQAWEDHFNDRILAFRASEFGWLSKFLYSICGNIIVLWSTPLLISTLTFGTAIVLGVPLDAGTVFTTTTV<br>FKILQEPLRTFPQSMISLSQALVSLGRLDRYMSSKELSNDSVEREEGCDGHIAVEVKDGSFSWDDDGQEQDLK<br>NINLEINKGELTAIVGTVGSGKSSLLASILGEMHRISGKVQVCGTTAYVAQTSWIQSGTIEENILFGLPMNRQK<br>YNEVVRVCCLEKDLEMMEYGDLTEIGERGINLSGGQKQRIQLARAVYQDSDVYLLDDVFSAVDAHTGTEIFK<br>ECVRGALKGKTIILVTHQVDFLHNVDLIVVMRDGMIAQSGKYNDLLDSGMGFSALVAAHETSMELVEQGAA<br>MPGGEHLNRSIKSPREAPNNREANGESNSLDQPKSGEENSKLVKEEERETGKVSLHIYKLYCTEAFGWWGITG<br>VILLSLLWQASMMASDYWLAYETSEERAQLFNPSVFISIYAIIAAVSIVLIVLRSYAVTILGLKTAQIFFSQILSSI<br>LHAPMSFFDTTPSGRILSRASTDQTNVDIFIPLFLNFVVAMYITVISIFIITCQNSWPTAFLLIPLLWLNIWYRGYF<br>LSSSRELTRLDSITKAPVIHHFSESISGVMTIRAFRKQREFCVENIKRVNSNLRMDFHNYSSNVWLGLRLELLGS<br>FFFCISTMFMIILPSSIIKPENVGLSLSYGLSLNSVLFWCVFQSCIIENKLVSVERIKQFTNIPSEPAWKIKDHVPPS<br>NWPGQGNVDIKDLQVRYRPNSPLVLKGITLSISGGEKIGVVGRTGSGKSTLIQVLFRLVEPSGGKVIVDSIDISV

			LGLHDLRSRFGIIPQEPVLFEGTVRSNIDPTGQYTDEEIWKSLERCQLKEVVGAKPEKLDSLVVDNGENWSVGQRQLLCLGRVMLKRSRLLFMDEATASVDSQTDGVIQKIIREDFAACTIISIAHRIPTVMDCDRVLVVDAGRVKEFDKPSNLLQRPSLFGALVQEYASRSTGL
76.	GgABCC20	MT379737	MSTWITSLSCSSPGDRTSTTTLPQWLRFVFLSPCPQRALLSAVDVLLLLTLFVFALIKLYSRFTSNRSTPNSELNKPLIGNTRASVRTTLWFKLTLTATVVLTIMYTVACILVFTSSTQVSWKQVDGLFWLLQAITQLVLAILIIHEKRFEAVSHPISLRIYWVASFVIVSLFTASGVIRFVSVDIREGKYFSFLVDDTVSFLTLPKSLFLLCVAIKGSTGIIKSSEETQPLVVEGETKLYDPITLTKSNVTGFASASAVSKAFWIWLNPLLSKGYKSPLNIDEVPLLSPQHRAERMSVIFESKWPKSDEKSKHPVRTTLLRCFWKEIAFTAFLAVVRLSVMFVGPVLIQDFVDFTSGKGSSIYEGYYLVLILLVAKFVEVLTTHHFNFNSQKLGMLIRCTLITSLYKKGLRLSCSARQDHGVGPIVNYMAVDTQQLSDMMLQLHAVWMMPFQVGIGLFLLYNCLGVSVVTALLGLLGVLAFIVSATRKNKRYQFSAMMNRDSRMKAVNEMLNYMRVIKFQAWEDHFNDRILAFRASEFGWLSKFLYSICGNIIVLWSTPLLISTLTFGTAIVLGVPLDAGTVFTTTTVFKILQEPLRTFPQSMISLSQALVSLGRLDRYMSSKELSNDSVEREEGCDGHIAVEVKDGSFSWDDDGQEQDLKNINLEINKGELTAIVGTVGSGKSSLLASILGEMHRISGKVQVCGTTAYVAQTSWIQSGTIEENILFGLPMNRQKYNEVVRVCCLEKDLEMMEYGDLTEIGERGINLSGGQKQRIQLARAVYQDSDVYLLDDVFSAVDAHTGTEIFKVVISYSQHSIHFLVDSSW
77.	GgABCC21	MT379738	MSLSYGLALSGLLSFTITMTCSVENKMVSVERIKQFTNLPSEAPWKIADKCPPQSWPSHGNIELNNLQVRYRPNTPLVLKGISLTIEGGEKVGVVGRTGSGKSTLIQVLFRLIEPSAGKIIIDGVNICTVGLHDVRSRFGIIPQEPVLFQGTVRSNIDPLGLYSEEEVWKSLERCQLKDVVAAKPEKLEASVVDGGDNWSVGQRQLLCLGRIMLKRSKILFMDEATASVDSQTDAVIQKIIREDFADRTIVTIAHRIPTVMDCDKVLVIDAGFAKEFDKPARLIERPSLFGALVKEYSNRSA
78.	GgABCC22	MT379739	MEDIWSMICGEYSGCSETGGKPYDFKFLKDIPSTCISHVFIIFFDVLMLIMLSFIMILKSWSRPFWSLVRYSKLQLVSAITNGSLGLFHLCLGIWVSEEMLRKTHKAFPLNFWLLELFQGFTWLLVSLTVSLQIKQLPRAWLWLFSILMFFVSFILCALSMSYATGSRELSLNAALDVLSFLGASLLLLCTYKACKCAEADREIGESLYAPLNSQFNEADPVSNRTTPFAKAGFFSKMWFWWLNPLMKRGQEKTLQDEDIPKLRNSDRAESCYLSFAEPFNRQKQNEASSHSSVLWTIILCHRREILVTGFFALLKVLTISSGPLLLNAFILVAEGNESFKYEGYVLAISLFFVKIIESLSQRQWYFHSRLVGMKVRSLLTAAIYKKALRLSNSARLVHSGGEIMNYVTVDAYRIGEFPFWFHQTWTTILQLCIALVILFHAVGLATIASLVVIVITVLCNTPLAKLQHKFQSQLMVAQDERLKASSEALVNMKVLKLYAWETHFRNAIENLRNVELKVLSSLQSKKAYNIFLFWTSPMLVSAASFLACYFLEIPLHASNVFTFVATLRLVQDPITAIPDVVGVIIQAKVAFARIVKFLEAPELQNSNFRKRGISDNLRGSIFIKSADFSWEGSGSKPTLRSINLEVRHGQKVAICGEVGSGKSTLLTTILGEVPNTKGTIEVCGTFAYVSQTAWIQTGTIRENILFGADLDDQRYQETLQRSSLMKDIELFPNGDLTEIGERGVNLSGGQKQRIQLARALYQNADLYLLDDPFSAVDAHTAKNLFNVMTYSFSSHSTCTCLVLFFTERLTALKLGIGIHHGRT
79.	GgABCC23	MT379740	MPFIQECVRGVLKDKTIILVTHQVDFLHNVDLILVMRDGMIVQSGKYNDLIDSGMDFKALVAAHETSMELVEQGAFIPGENLNKPAKSPETASIYKGTNGESNSPDKPESGNNKSSKLIKEEEKEIGKVSLHIYKLYCTEAFGWWGITGVLILSLLWQGSLMASDYWLAYETSEERAQMFNPSLFVSVYAIITLVSVAFVVMRCYSFTFLGLKTAQNFFTQILHSILHAPMSFFDTTPSGRILSRASTDQTNVDVTLPMFMGIAITMYITVLSILIITCQYSWPTMLLIIPLVWLNIWYRGYFLASSRELTRLDSITKAPIIHHFSESIAGVMTIRAFRKQKRFCEENLKRVNANLRMDFHNYSSNVWLGLRLELLGSFFFCISTMFMIILPSSIIKPENVGLSLSYGLSLNSVLFWCVFQSCIIENKLVSVERIKQFTNIPSEPAWKIKDHVPPSNWPGQGNVDIKDLQVRYRPNSPLVLKGITLSISGGEKIGVVGRTGSGKSTLIQVLFRLVEPSGGKVIVDSIDISVLGLHDLRSRFGIIPQEPVLFEGTVRSNIDPTGQYTDEEIWKSLERCQLKEVVGAKPEKLDSLVVDNGENWSVGQRQLLCLGRVMLKRSRLLFMDEATASVDSQTDGVIQKIIREDFAACTIISIAHRIPTVMDCDRVLVVDAGRVKEFDKPSNLLQRPSLFGALVQEYASRSTGL
80.	GgABCC24	MT379741	MFFLQFYCIWLFIMRLHFHSLTSTLSLFKVRYKENLPLVLHGVSCTFPGGKKIGIVGRTGSGKSTLIQALFRLIEPADGSILIDNINISEIGLHDLRSHLSIIPQDPTLFEGTIRGNLDPLEEHSDKEIWEALEKSQLGEIIHQKGQKLDTAVLENGDNWSVGQRQLVSLGRALLKQSKILVLDEATASVDTATDNLIQKIIRTEFKDCTVCTIAHRIPTVIDSDQVLVLSDGRVAEFDTPHRLLEDKSSMFLKLVTEYSSRSSGIPEF
81.	GgABCC25	MT379742	MELQEYILTALADKTIIFVTHQVEFLPATDLILVLKEGCIIQAGKYDDLLQAGTDFKTLVSAHHEAIEAMDIPAHSSEDSDENLSLEASVMTSKKSMCSANDIDSLAKEMQDGSSASDQKSIKEKKKTKRSRKKQLVQEEERIRGRVSMKVYLSYMAAAYKGLLIPLIIIAQALFQFLQIASNWWMAWANPQTEGDQPKVTPTMLLLVYMALAFGSSWFIFVRAVLVATFGLAAAQKLFLNMLRCVFHAPMSFFDSTPAGRILNRVSVDQSVVDLDIPFRLGGFAATTIQLIGIVGVMTEVTWQVLLLVIPMAVACLWMQKYYMASSRELVRIVSIQKSPIINLFGESIAGASTIRGFGQEKRFMKRNLYLLDCFARPFFCSLAAIEWLCLRMELLSTFVFAFCMVLLVSFPRGSIDPSMAGLAVTYGLNLNARLSRWILSFCKLENKIISIERIYQYSQIPSEAPAIIEDSRPPSSWPESGTIEIIDLKVRYKENLPLVLHGVSCTFPGGKKIGIVGRTGSGKSTLIQALFRLIEPADGSILIDNINISEIGLHDLRSHLSIIPQDPTLFEGTIRGNLDPLEEHSDKEIWEALEKSQLGEIIHQKGQKLDTAVLENGDNWSVGQRQLVSLGRALLKQSKILVLDEATASVDTATDNLIQKIIRTEFKDCTVCTIAHRIPTVIDSDQVLVLSDGRVAEFDTPHRLLEDKSSMFLKLVTEYSSRSSGIPEF
82.	GgABCC26	MT379743	MDKAKYKNVIHACSLKKDLELFSHGDQTIIGDRGINLSGGQKQRVQLARALYQDSDIYLLDDPFSAVDAHTGSELFREYILTALADKTIIFVTHQVEFLPATDLILVLKEGCIIQAGKYDDLLQAGTDFKTLVSAHHEAIEAMDIPAHSSEDSDENLSLEASVMTSKKSMCSANDIDSLAKEMQDGSSASDQKSIKEKKKTKRSRKKQLVQEEERIRGRVSMKVYLSYMAAAYKGLLIPLIIIAQALFQFLQIASNWWMAWANPQTEGDQPKVTPTMLLLVYMALAFGSSWFIFVRAVLVATFGLAAAQKLFLNMLRCVFHAPMSFFDSTPAGRILNRVSVDQSVVDLDIPFRLGGFAATTIQLIGIVGVMTEVTWQVLLLVIPMAVACLWMQKYYMASSRELVRIVSIQKSPIINLFGESIAGASTIRGFGQEKRFMKRNLYLLDCFARPFFCSLAAIEWLCLRMELLSTFVFAFCMVLLVSFPRGSIDPSMAGLAVTYGLNLNARLSRWILSFCKLENKIISIERIYQYSQIPSEAPAIIEDSRPPSSWPESGTIEIIDLKVRYKENLPLVLHGVSCTFPGGKKIGIVGRTGSGKSTLIQALFRLIEPADGSILIDNINISEIGLHDLRSHLSIIPQDPTLFEGTIRGNLDPLEEHSDKEIWEALEKSQLGEIIHQKGQKLDTAVLENGDNWSVGQRQLVSLGRALLKQSKILVLDEATASVDTATDNLIQKIIRTEFKDCTVCTIAHRIPTVIDSDQVLVLSDGRVAEFDTPHRLLEDKSSMFLKLVTEYSSRSSGIPEF
83.	GgABCC27	MT379744	MSSPSWLTSPSCTLLAIDSSSSSSTPQLIVQWLRFIFLSPCPQRVLLSTLDLLFLLSLLAFAAHRLYSRFTSNTNSNSSITKPLLQEKDSDYRVTLWFKLPLLVTALLAIAYTVLGILVFTQTNLASSWRQIEALFRLSQAIANIVVVALMVHEKKFKASKHPLSLRIYWIANFVLACFFAASAIVRFVTVGEEWLELSLRIDDIFSLVNLPLSVFFFVIAIKGSSGIHVIRISDVVTTTYQLLSTDRTLSPYARASIISKTVWLWMNPLINKGYKTPLKLEDVPSLPLEFRAEKMSELFQS

			KWPKPEENSKHPVGLTLFRCFWKRIAFTGFLAIIRLSVMYVGPMLIQSFVDYTSRTDSTPNEGVVLVLILFAAK<br>SIEVLTVHQFNFNSQKLGMLIRSSIITSVYKKGLRLSSSSRQAHGTGQIVNHMAVDAQQLSDLMMQFHPIWM<br>MPLQVAAALALMYAYVGLSVLSALIGTSLVFLFTLFRTKRSNNFQFRIMMSRDLRMKAINELLNNMRVIKFQ<br>AWEEYFGNKIRQFREAEHGWIGKFLYYFAVNMGVLSTAPLTITVLTFGTATFMGIPLNAGTVFTITSVIKILQE<br>PVRTFPQALIQISQAVISLGRLDEFMMSKEMDEDAVERKDNCDSDIAVEIKDGKFSWDDQDGNEALKVEELEI<br>RKGDRAAVVGTVGSGKSSLLASVLGEMFKISGKVRVCGTIAYVAQTSWIQNATIQENILFGLPMNREKYREAI<br>RVCCLEKDLEMMDDGDKTEIGERGINLSGGQKQRIQLARAVYQDCDIYLLDDVFSAVDAQTGSFIFKECIMG<br>ALKDKTLLLVTHQVDFLHNVDSIMVMRDGTIVQTGKYDELLKAGLDFGALVAAHESSMEIAETSDKGGDDS<br>GQSPKLARIPSKEKESTGEKQPQDQSKSDDKAAAKLIEEEERETGRVNLKVYKHYFTEAFGWWGIALMVAM<br>SLAWMLSFLAGDYWLAFATSEDSGIASFTFIMVYAIIAVVACVVVMARGFLFTYWGLKTSQSFFVGMIQSILH<br>APMSFFDTTPSGRILSRVSSNLPF
84.	GgABCC28	MT379745	MVGFWSVFCGESGCSEAGRKPCSYDFKLLIDPSACINHLLISCFDVLLLIMLLFIMIQKSSLKGPFQGHIRGQRY<br>SNLQLVSAIANGALGLVHLFLGIWILEENLRKTQTALPLDLWLLEFFQGLTWLLVGLTLSLKLKQLPRTWLRL<br>SSILIFLVSGIFCALSLFYAISSGELSLKVALDVLSFSGAILLVLCTYKESRRRETDREIDESLYTPLNGESNKNDS<br>VIQVTRFAKAGFFSRMSFWWLNPLMKRGKEKTLNDKDVPKLREEDRAESCYSLFLDRLSKQKQKDPSSQPSV<br>LRTLILCHRREILISGFFALLKVLALSSGPLLLNSFILVAEGYESFKYEGFVLAISLFFTKIIESLSQRQWYFRSRLI<br>GLKVRSLLTAAIYRKQLRLSNSARLMHSGGEIMNYVTVDAYRIGEFPYWFHQTWTTSFQLCISLVILFRAVGL<br>ATIASLVVILITVLCNTPLAKLQHKFQSKLMVAQDERLKATSEALVNMKVLKLYAWETNFKNAIEGLRNVEL<br>KWLSAVQLRKAYNTFLFWSSPVLVSAASFGACYFLNVPLHANNVFTFVATLRLVQDPIRTIPDVIGVVIQAKV<br>ALTRIVKFLEAPELQNANVRKRFSNDNMRGSISIKSANFSWEDSDVSKPTLRNINLEVRPGQKVAICGEVGSG<br>KSTLLAAILREVPNTQGTIDVYGKFAYVSQTAWIQTGTIRENILFGSAMDAQKYEETLHRSSLLKDLELFPHGD<br>LTEIGERGLNLSGGQKQRIQLARALYQNADIYLLDDPFSAVDAQTATNLFNEYIVEGLAGKTVLLVTHQVDFL<br>PAFDSVLLMSDGEILHDAPYHHLLSTSQEFQDLVNAHKETAGSDRLVDVTSCQRYSSSAREIKKTYGEKEKQF<br>EESKGDQLIKQEEREIGDQGFKPYLQYLNQNKGYVYFSVASLSHLVFVIGQILQNSWMAANVDNPQVSTLRLI<br>LVYLLIGVTSTLFLLMRSLLTVALGLQSSKSLFLQLLNTLFRAPMSFYDSTPLGRILSRVSSDLSIVDLDVPFGLL<br>FAVVATTNCYANLTVLAVVTWQVLFVSIPMIYLALRLQKYYFASAKELMRMNGTTKSFVANHLAESVAGAV<br>TIRAFEEEDRFFVKNLDLIDINASAFFHNFASNEWLIQRLETISAVVLVSAALCMVMLPPGTFTSGFIGMALSYG<br>LSLNASLVFSIQSQCTLANYIISVERLNQYMHIQSEAQEVIEGNRPPLNWPVAGKVEIKDLKIRYRPDEPLVLHG<br>ITCTFKAGHKIGIVGRTGSGKSTLIGALFRLVEPAGGKIVVDGIDILSIGLHDLRSRFGVIPQDPTLFNGTVRFNL<br>DPLSQHTDQEIWEVKT
85.	GgABCC29	MT379746	MSSPSWLTSPSCTLLAIDSSSSSSTPQLIVQWLRFIFLSPCPQRVLLSTLDLLFLLSLLAFAAHRLYSRFTSNTNSN<br>SSITKPLLQEKDSDYRVTLWFKLPLLVTALLAIAYTVLGILVFTQTNLASSWRQIEALFRLSQAIANIVVVALM<br>VHEKKFKASKHPLSLRIYWIANFVLACFFAASAIVRFVTVGEEWLELSLRIDDIFSLVNLPLSVFFFVIAIKGSSG<br>IHVIRISDVVTTTYQLLSTDRTLSPYARASIISKTVWLWMNPLINKGYKTPLKLEDVPSLPLEFRAEKMSELFQS<br>KWPKPEENSKHPVGLTLFRCFWKRIAFTGFLAIIRLSVMYVGPMLIQSFVDYTSRTDSTPNEGVVLVLILFAAK<br>SIEVLTVHQFNFNSQKLGMLIRSSIITSVYKKGLRLSSSSRQAHGTGQIVNHMAVDAQQLSDLMMQFHPIWM<br>MPLQVAAALALMYAYVGLSVLSALIGTSLVFLFTLFRTKRSNNFQFRIMMSRDLRMKAINELLNNMRVIKFQ<br>AWEEYFGNKIRQFREAEHGWIGKFLYYFAVNMGVLSTAPLTITVLTFGTATFMGIPLNAGTVFTITSVIKILQE<br>PVRTFPQALIQISQAVISLGRLDEFMMSKEMDEDAVERKDNCDSDIAVEIKDGKFSWDDQDGNEALKVEELEI<br>RKGDRAAVVGTVGSGKSSLLASVLGEMFKISGKVRVCGTIAYVAQTSWIQNATIQENILFGLPMNREKYREAI<br>RVCCLEKDLEMMDDGDKTEIGERGINLSGGQKQRIQLARAVYQDCDIYLLDDVFSAVDAQTGSFIFKVSAKV<br>KLQALLNSSSS
86.	GgABCC30	MT379747	MHPAATDFLLKPIFLHGLSSLLHLLLLVAVLVSWVWNKITTCSVNDSNKEKLNNPLFTVTKFCSLGVSAFNLV<br>LSLYNYFYWYRSGWSEEKLVTLLDLALKTVAWGVVYVCLHKGFFSRRRRRFPFFFTAWCAFYLFASCYIFVV<br>DIVVLYEKHIALTAQRIVSDVVSACVGSFFCYVGYCVKNEGEDSNILQEPLLNGDSAHVSKETRGSDTVTPFS<br>NAGILSILTFSWVGPLVAFGNKKTLDLEDVPLLDSGDSVVGAFPTFRDKVEADCGAINRVTTLKLVKSLIISAW<br>KEILFTAFLALVNTLASYVGPYLIDAFVQYLGGQRLYENQGYVLVSAFFFAKLVECLSQRHWFFRLQQIGIRIR<br>ALLVTMIYNKALTLSCQSKQGQTSGEIINFMTVDAERVGVFSWYLHDLWLVVLQVSLALLILYKNLGLASIAT<br>FVATVIVMLANVPLGSLLEKFQNKLMKSKDTRMKSTSEILRNMRILKLQGWEMKFLSKITELRNTEQGWLRK<br>FVYTSAITTFVFWGAPTFVSVVTFGTCMLIGIPLESGKILSALATFRILQEPIYSLPDTISMIAQTKVSLDRIASFL<br>RLDDLQSDVVERLPRDSSDTAIEVVDGNFSWDISSRNPTLQNINLRVFHGMRVAVCGTVGSGKSSLLSCIIGEV<br>PKLSGTLKVCGTKAYVAQSPWIQSGKIEENILFGREMDREKYEKVLEACALIKDLEVLPFGDQTIIGEKGINLS<br>GGQKQRVQIARALYQDADIYLLDDPFSAVDAHTGSHIFKECLLGLLKAKTVIYITHQVEFLPDADLILVMKEG<br>RITQSGKYNDILRSGTDFMELVGAHRAALSSVKSLERRHTFKSSSITMKDTDSLSDFEHEQEVENIDDQIGKSE<br>DTIEPKGQLVQEEEREKGRVGFKVFWKYITTAYGGALVPFILLSQALTVVLQIGSNYWMALATPVSATAEPDI<br>GSFTLMVVYVSLAIGSSFTTLARAVLAVIAGYKTATMLFNKMHLCFFRAPMSFFDATPSGRILNRASTDQSAI<br>DMSMPNLAWGFTYNLIQLFGNIAVMSQAAWQVFIVLIPVMAACIWYQRYYAASARELARLVGICQAPVIQHF<br>SETISGSTTIRSFEQESRFNEINMKLIEKYSQPKLYSASAMEWLSFRLDILSSTMFAFCLVFLISFPSSIADPGE
87.	GgABCC31	MT379748	MHPAATDFLLKPIFLHGLSSLLHLLLLVAVLVSWVWNKITTCSVNDSNKEKLNNPLFTVTKFCSLGVSAFNLV<br>LSLYNYFYWYRSGWSEEKLVTLLDLALKTVAWGVVYVCLHKGFFSRRRRRFPFFFTAWCAFYLFASCYIFVV<br>DIVVLYEKHIALTAQRIVSDVVSACVGSFFCYVGYCVKNEGEDSNILQEPLLNGDSAHVSKETRGSDTVTPFS<br>NAGILSILTFSWVGPLVAFGNKKTLDLEDVPLLDSGDSVVGAFPTFRDKVEADCGAINRVTTLKLVKSLIISAW<br>KEILFTAFLALVNTLASYVGPYLIDAFVQYLGGQRLYENQGYVLVSAFFFAKLVECLSQRHWFFRLQQIGIRIR<br>ALLVTMIYNKALTLSCQSKQGQTSGEIINFMTVDAERVGVFSWYLHDLWLVVLQVSLALLILYKNLGLASIAT<br>FVATVIVMLANVPLGSLLEKFQNKLMKSKDTRMKSTSEILRNMRILKLQGWEMKFLSKITELRNTEQGWLRK<br>FVYTSAITTFVFWGAPTFVSVVTFGTCMLIGIPLESGKILSALATFRILQEPIYSLPDTISMIAQTKVSLDRIASFL<br>RLDDLQSDVVERLPRDSSDTAIEVVDGNFSWDISSRNPTLQNINLRVFHGMRVAVCGTVGSGKSSLLSCIIGEV<br>PKLSGTLKVCGTKAYVAQSPWIQSGKIEENILFGREMDREKYEKVLEACALIKDLEVLPFGDQTIIGEKGINLS<br>GGQKQRVQIARALYQDADIYLLDDPFSAVDAHTGSHIFKECLLGLLKAKTVIYITHQVEFLPDADLILVMKEG<br>RITQSGKYNDILRSGTDFMELVGAHRAALSSVKSLERRHTFKSSSITMKDTDSLSDFEHEQEVENIDDQIGKSE<br>DTIEPKGQLVQEEEREKGRVGFKVFWKYITTAYGGALVPFILLSQALTVVLQIGSNYWMALATPVSATAEPDI<br>GSFTLMVVYVSLAIGSSFTTLARAVLAVIAGYKTATMLFNKMHLCFFRAPMSFFDATPSGRILNRVCHNMLII

			LHWFILKRQLIYFDHGLVHYF
88.	GgABCC32	MT379749	MSQAILCLFCIMNALFTLVRAFSFAFGGLQAATKVHNRLLSQLINAPVQFFDQTPGGRILNRLSSDLYTIDDSLP<br>FIMNILLANFVGLLGIAIILSYVQVFFLVLLLPFWYIYSRLQFFYRSTSRELRRLDSVSRSPIYMSFTETLDGSSTI<br>RAFKSEDFFFAKFTEHITLYQKTSYTEIVASLWLSLRLQLLAAFIISFIALMAVVGSHGNLPINFGTPGLVGLALS<br>YAAPIVSLLGSFLTSFTETEKEMVSVERVLQYMDIPREEQAGCLYLNPDWPDQGVIEFQHVTLKYMPSLPAAL<br>CNLSFRIEGGTQVGIIGRTGAGKSSVLNALFRLTPICAGSITVDGMDIQNIPVRELRTHLAIVPQSPFLFEGSLRD<br>NLDPLKMNDDFKIWNALEKCHVKEEVEVAGGLDITVKESGMSFSVGQRQLLCLARALLKSSKVRTIT
89.	GgABCC33	MT379750	MSFTETLDGSSTIRAFKSEDFFFAKFTEHITLYQKTSYTEIVASLWLSLRLQLLAAFIISFIALMAVVGSHGNLPI<br>NFGTPGLVGLALSYAAPIVSLLGSFLTSFTETEKEMVSVERVLQYMDIPREEQAGCLYLNPDWPDQGVIEFQH<br>VTLKYMPSLPAALCNLSFRIEGGTQVGIIGRTGAGKSSVLNALFRLTPICAGSITVDGMDIQNIPVRELRTHLAI<br>VPQSPFLFEGSLRDNLDPLKMNDDFKIWNALEKCHVKEEVEVAGGLDITVKESGMSFSVGQRQLLCLARALL<br>KSSKVRTIT
90.	GgABCC34	MT379751	MNLTDGYSRPKFNSAAAMEWLCIRLDMLSSITFAFSLIFLISIPQGIIDPGIAGLAVTYGLNLNMIQAWVIWNLC<br>NLENKIISVERILQYTSIPSEPPLAVEENRPDPSWPSHGEVDIRDLQVRYAPHLPLVLRGLTCTFRGGLKTGIVG<br>RTGSGKSTLIQTLFRIVEPTAGQVIIDGIDISSIGLHDLRSRLSIIPQEPTMFEGTVRNNLDPLEEYTDEQIWEALD<br>KCQLGDEVRKKEGKLDSTVSENGENWSMGQRQLVCLGRVLLKKSKVLVLDEATASVDTATDNLIQQTLRQ<br>HFSDSTVITIAHRITSVLDSDMVLLLSQGLIEEYDSPTTLLEDKSSSFAQLVAEYTMRSNSSFEKSVDL
91.	GgABCC35	MT379752	MKDGKITQCGKYADLLNSGTDFMELVGAHRKALSALDSLDGGTVSNDEISTLDQDVDVFGTDGVKEKEANK<br>DEQNGKADDKGEPKGQLVQEEEREKGKVGFSVYWNYITTAYGGALVPFILLAQILFQALQIGSNYWMAWAT<br>PISSDVDPPVGGSTLIEVYVGLAIGSAFCILARAILLATVGYKTATILFNKMHLCIFRAPMSFFDSTPSGRILNRA<br>STDQSAVDTDIPYQIGSFAFSMIQLLGIIAVMSQVAWQVFIVFIPVIGVSIWYQQYYLPSARELSRLVGVCKAPII<br>QHFAETISGTSTIRSFDQQSRFQETNMNLTDGYSRPKFNSAAAMEWLCIRLDMLSSITFAFSLIFLISIPQGIIDPG<br>IAGLAVTYGLNLNMIQAWVIWNLCNLENKIISVERILQYTSIPSEPPLAVEENRPDPSWPSHGEVDIRDLQVRY<br>APHLPLVLRGLTCTFRGGLKTGIVGRTGSGKSTLIQTLFRIVEPTAGQVIIDGIDISSIGLHDLRSRLSIIPQEPTMF<br>EGTVRNNLDPLEEYTDEQIWEALDKCQLGDEVRKKEGKLDSTVSENGENWSMGQRQLVCLGRVLLKKSKV<br>LVLDEATASVDTATDNLIQQTLRQHFSDSTVITIAHRITSVLDSDMVLLLSQGLIEEYDSPTTLLEDKSSSFAQL<br>VAEYTMRSNSSFEKSVDL
92.	GgABCC36	MT379753	MKDGKITQCGKYADLLNSGTDFMELVGAHRKALSALDSLDGGTVSNDEISTLDQDVDVFGTDGVKEKEANK<br>DEQNGKADDKGEPKGQLVQEEEREKGKVGFSVYWNYITTAYGGALVPFILLAQILFQALQIGSNYWMAWAT<br>PISSDVDPPVGGSTLIEVYVGLAIGSAFCILARAILLATVGYKTATILFNKMHLCIFRAPMSFFDSTPSGRILNRA<br>STDQSAVDTDIPYQIGSFAFSMIQLLGIIAVMSQVAWQVFIVFIPVIGVSIWYQQYYLPSARELSRLVGVCKAPII<br>QHFAETISGTSTIRSFDQQSRFQETNMNLTDGYSRPKFNSAAAMEWLCIRLDMLSSITFAFSLIFLISIPQGIIDPG<br>IAGLAVTYGLNLNMIQAWVIWNLCNLENKIISVERILQYTSIPSEPPLAVEENRPDPSWPSHGEVDIRDLQVRY<br>APHLPLVLRGLTCTFRGGLKTGIVGRTGSGKSTLIQTLFRIVEPTAGQVIIDGIDISSIGLHDLRSRLSIIPQEPTMF<br>EGTVRNNLDPLEEYTDEQIWEALDKCQLGDEVRKKEGKLDSTVSENGENWSMGQRQLVCLGRVLLKKSKV<br>LVLDEATASVDTATDNLIQQTLRQHFSDSTVITIAHRITSVLDSDMVLLLSQGQ
93.	GgABCC37	MT379754	MFGGTVRSNMDPLEEYTDEQIWEALDMCQLGDEVRKKEGKLDSIVTENGENWSMGQRQLVCLGRVLLKKS<br>KILVLDEATASVDTATDNIIQQTVKQHFSECTVITIAHRITSILDSDMVLFLNEGLVEEYDSPNKLLKNKSSSLA<br>QLVAEYTRRSNSGFGS
94.	GgABCC38	MT379755	MFGGTVRSNMDPLEEYTDEQIWEALDMCQLGDEVRKKEGKLDSIVTENGENWSMGQRQLVCLGRVLLKKS<br>KILVLDEATASVDTATDNIIQQTVKQHFSECTVITIAHRITSILDSDMVLFLNEGNNLFLFLLNLSPLFCFFTHKC<br>EHDFFSYRSRRAC
95.	GgABCC39	MT379756	MLGELPPIADSTVVMRGTVAYVPQVSWIFNATVRDNVLFGSVFDPIRYERAIDVTELQHDLELLPGGDLTEIG<br>ERGVNISGGQKQRVSMARSVYSNSDVYIFDDPLSALDAHVARQVFDKCIKGELRGKTRVLVTNQLHFLSQVD<br>RIILVHDGMVKEEGTFEELSNQGPLFQKLMENAGKMEEYEGEEEKVDIEATDQKSSSKPVANGAVNDYTKSE<br>SKPKEGKSVLIKQEERETGVVSWNVLARYKNALGGSWVVLILFGCYVSTEVLRVSSSTWLSHWTDESALEGY<br>NPSFYNLIYAALSFGQVLVTLTNSYWLIISSLYAARRLHEAMLHSILRAPMVFFQTNPLGRVINRFAKDLGDID<br>RNVAPFVNMFLSQVSQLLSTFILIGIVSTMSLWVIMPLLVLFYAAYLYYQSTAREVKRLDSISRSPVYAQFGEA<br>LNGLSTIRAYKAYDRMADINGRSMDNNIRFTLVNIGGNRWLAIRLETLGGLMIWFAATFAVMQNGRAENQQ<br>EYASSMGLLLSYALNITNLLTGVLRLASLAENSLNSVERVGTYINLPSEAPYVIEDNRPPPGWPSSGSIKFEEVV<br>LRYRPELPPVLHGISFTIFPSDKVGIVGRTGAGKSSMLNALFRIVELEKGRILIDGCDIAKFGLADLRKVLGIIPQ<br>SPVLFSGTVRFNLDPFNEHNDADLWEALERAHLKDVIRRNSLGLDAEVSEAGENFSVGQRQLLSLSRALLRRS<br>KILVLDEATAAVDVRTDALIQKTIREEFKSCTMLIIAHRLNTIIDCDRILLLDGGKVCAQYSKFRPQTIELPNS
96.	GgABCC40	MT379757	MLQYHCHIILMMNIYKKVEYGADFIPFLLAFLYVYICLIIVLSWGPFRYKNALGGSWVVLILFGCYVSTEVLRV<br>SSSTWLSHWTDESALEGYNPSFYNLIYAALSFGQVLVTLTNSYWLIISSLYAARRLHEAMLHSILRAPMVFFQT<br>NPLGRVINRFAKDLGDIDRNVAPFVNMFLSQVSQLLSTFILIGIVSTMSLWVIMPLLVLFYAAYLYYQSTAREV<br>KRLDSISRSPVYAQFGEALNGLSTIRAYKAYDRMADINGRSMDNNIRFTLVNIGGNRWLAIRLETLGGLMIWF<br>AATFAVMQNGRAENQQEYASSMGLLLSYALNITNLLTGVLRLASLAENSLNSVERVGTYINLPSEAPYVIEDN<br>RPPPGWPSSGSIKFEEVVLRYRPELPPVLHGISFTIFPSDKVGIVGRTGAGKSSMLNALFRIVELEKGRILIDGCDI<br>AKFGLADLRKVLGIIPQSPVLFSGTVRFNLDPFNEHNDADLWEALERAHLKDVIRRNSLGLDAEVSEAGENFS<br>VGQRQLLSLSRALLRRSKILVLDEATAAVDVRTDALIQKTIREEFKSCTMLIIAHRLNTIIDCDRILLLDGGKVC<br>AQYSKFRPQTIELPNS
97.	GgABCC41	MT379758	MFSVSLKLVKINLESLNLWFFLSEENNIVDAVSLPLLLLLCFNALPNVCEREQSEMEQRLLQKEMESSVEEDE<br>DEEAFTKASRWSKLTFRWLNPIFSRGRIQKLEHAHIPSVPRSEAAESASSMLEESLRKQKLEGGSLVKAITHSI<br>WKSLALNAVLAGVNTIAAYIGPLLISSFVNFLLSNDNNNSSIQHGLILAFVFFLSKTVESLSQRQWYFGAQRIGI<br>RVRAALMALVYSKSLMIKCAGPTHGKIINLINVDVDRIGDFCWYIHGVWLLPVQVILALVILYINLGCAPSIAA<br>LTVTILVMVFNTPLANMQENLHSKIMEAKDSRIKMTSETMKNIRILKLHSWESTFLQKLLQLRDTERRWLKK<br>YLYTCSAVATLFWASPTLVSVATFGACILVKTELTAATVLSALATFRILQEPIYNLPELISMITQTKVSIDRIQEF<br>IKEEDQNQFMNRHAPHTSAIAIEIKPGEYAWDANDQTHKKPTIRITEKIMIKKGQKVAVCGSVGSGKSSFLCT<br>MLGEIPLVSGAVTKVYGTRSYVPQSPWIQSGTVRENILFGKQMQKDFYENVLDGCALHQDINTWSDGDLTLV<br>EERGINLSGGQKQRIQLARAVYNDSDIYFLDDPFSAVDAHTGSHLFKKCLMKLLCDKTVVYATHQLEFLEAA

			DLILVMKDGKIVESGRYADLIACPSSELVQQMAAHKETITQIPCHKDDFISCRPCQKNPTEIAEENIQEIIMDWK<br>RTREEEAMTGRVKWSVYSTFVTLAYRGVLVPVILLCQILFQVMQMGSNYWISWATEQKGRVDNRELMGTF<br>VLLSGGSSIFIMGRTVLMATVAVETAQRLFHGMITSVFRAPVSFFDTTPSSRILSRSSTDQSTVDTDIPYRLAGL<br>VFALIQLLSIIMLMSQVAWQVILLFFVVLAISIWYQAYYITTARELARMVGIRKAPILHHFSESIAGAATIRCFN<br>QEQIFLKKVMALIDDYSRVAFHNYATMEWLSVRINFLFNLVFYVVLVILVTLPRSAIDPSLAGLVATYGLNLN<br>VLQAWVIWNLCNVENKMISVERILQFSSIPSEAPLIIQDCRPDPEWPRDGKIELHNLHIQYDPAAPMVLKGVTC<br>VFPGQKKIGVVGRTGSGKSTLVQALFRVVEPSEGHILIDGIHICNIGLQDLRSKLGIIPQDPTLFQGTVRTNLDPL<br>EEHSDQELWEVLRKCHLAEIVRQDARLLDTPVAENGENWSVGQRQLVCLARLLLKKRRILVLDEATASIDTT<br>TDNLIQKTIREETNGCTVITVAHRIPTVIDNDLVLVLDQGKIVEYDRPAQLLQNNSSSFSKLVSEFLSRSSQSTC
98.	GgABCC42	MT379759	MKLLCDKTVVYATHQLEFLEAADLILVMKDGKIVESGRYADLIACPSSELVQQMAAHKETITQIPCHKDDFIS<br>CRPCQKNPTEIAEENIQEIIMDWKRTREEEAMTGRVKWSVYSTFVTLAYRGVLVPVILLCQILFQVMQMGSN<br>YWISWATEQKGRVDNRELMGTFVLLSGGSSIFIMGRTVLMATVAVETAQRLFHGMITSVFRAPVSFFDTTPSS<br>RILSRSSTDQSTVDTDIPYRLAGLVFALIQLLSIIMLMSQVAWQVILLFFVVLAISIWYQAYYITTARELARMVG<br>IRKAPILHHFSESIAGAATIRCFNQEQIFLKKVMALIDDYSRVAFHNYATMEWLSVRINFLFNLVFYVVLVILVT<br>LPRSAIDPSLAGLVATYGLNLNVLQAWVIWNLCNVENKMISVERILQFSSIPSEAPLIIQDCRPDPEWPRDGKIE<br>LHNLHIQYDPAAPMVLKGVTCVFPGQKKIGVVGRTGSGKSTLVQALFRVVEPSEGHILIDGIHICNIGLQDLRS<br>KLGIIPQDPTLFQGTVRTNLDPLEEHSDQELWEVLRKCHLAEIVRQDARLLDTPVAENGENWSVGQRQLVCL<br>ARLLLKKRRILVLDEATASIDTTTDNLIQKTIREETNGCTVITVAHRIPTVIDNDLVLVLDQGKIVEYDRPAQLL<br>QNNSSSFSKLVSEFLSRSSQSTC
99.	GgABCC43	MT379760	MMKVLQVLELSTKIIPFCQVMKDGKIVESGRYADLIACPSSELVQQMAAHKETITQIPCHKDDFISCRPCQKNP<br>TEIAEENIQEIIMDWKRTREEEAMTGRVKWSVYSTFVTLAYRGVLVPVILLCQILFQVMQMGSNYWISWATE<br>QKGRVDNRELMGTFVLLSGGSSIFIMGRTVLMATVAVETAQRLFHGMITSVFRAPVSFFDTTPSSRILSRSSTD<br>QSTVDTDIPYRLAGLVFALIQLLSIIMLMSQVAWQVILLFFVVLAISIWYQAYYITTARELARMVGIRKAPILHH<br>FSESIAGAATIRCFNQEQIFLKKVMALIDDYSRVAFHNYATMEWLSVRINFLFNLVFYVVLVILVTLPRSAIDPS<br>LAGLVATYGLNLNVLQAWVIWNLCNVENKMISVERILQFSSIPSEAPLIIQDCRPDPEWPRDGKIELHNLHIQY<br>DPAAPMVLKGVTCVFPGQKKIGVVGRTGSGKSTLVQALFRVVEPSEGHILIDGIHICNIGLQDLRSKLGIIPQDP<br>TLFQGTVRTNLDPLEEHSDQELWEVLRKCHLAEIVRQDARLLDTPVAENGENWSVGQRQLVCLARLLLKKR<br>RILVLDEATASIDTTTDNLIQKTIREETNGCTVITVAHRIPTVIDNDLVLVLDQGKIVEYDRPAQLLQNNSSSFS<br>KLVSEFLSRSSQSTC
100.	GgABCC44	MT379761	MLHSILRAPMVFFQTNPLGRVINRFAKDLGDIDRNVAPFVNMFLSQVSQLLSTFILIGIVSTMSLWVIMPLLVLF<br>YAAYLYYQSTAREVKRLDSISRSPVYAQFGEALNGLSTIRAYKAYDRMADINGRSMDNNIRFTLVNIGGNRW<br>LAIRLETLGGLMIWFAATFAVMQNGRAENQQEYASSMGLLLSYALNITNLLTGVLRLASLAENSLNSVERVG<br>TYINLPSEAPYVIEDNRPPPGWPSSGSIKFEEVVLRYRPELPPVLHGISFTIFPSDKVGIVGRTGAGKSSMLNALF<br>RIVELEKGRILIDGCDIAKFGLADLRKVLGIIPQSPVLFSGI
101.	GgABCC45	MT379762	MVKEEGTFEELSNQGPLFQKLMENAGKMEEYEGEEEKVDIEATDQKSSSKPVANGAVNDYTKSESKPKEGK<br>SVLIKQEERETGVVSWNVLARYKNALGGSWVVLILFGCYVSTEVLRVSSSTWLSHWTDESALEGYNPSFYNL<br>IYAALSFGQVLVTLTNSYWLIISSLYAARRLHEAMLHSILRAPMVFFQTNPLGRVINRFAKDLGDIDRNVAPFV<br>NMFLSQVSQLLSTFILIGIVSTMSLWVIMPLLVLFYAAYLYYQSTAREVKRLDSISRSPVYAQFGEALNGLSTIR<br>AYKAYDRMADINGRSMDNNIRFTLVNIGGNRWLAIRLETLGGLMIWFAATFAVMQNGRAENQQEYASSMG<br>LLLSYALNITNLLTGVLRLASLAENSLNSVERVGTYINLPSEAPYVIEDNRPPPGWPSSGSIKFEEVVLRYRPEL<br>PPVLHGISFTIFPSDKVGIVGRTGAGKSSMLNALFRIVELEKGRILIDGCDIAKFGLADLRKVLGIIPQSPVLFSGI
102.	GgABCC46	MT379763	MSLSYGLALSGLLSFTITMTCSVENKMVSVERIKQFTNLPSEAPWKIADKCPPQSWPSHGNIELNNLQVRYRP<br>NTPLVLKGISLTIEGGEKVGVVGRTGSGKSTLIQVLFRLIEPSAGKIIIDGVNICTVGLHDVRSRFGIIPQEPVLFQ<br>GTVRSNIDPLGLYSEEEVWKVYILHYLVCFFCF
103.	GgABCC47	MT379764	MHLVTYIIILLILKWCEQVRYRPNTPLVLKGISLTIEGGEKVGVVGRTGSGKSTLIQVLFRLIEPSAGKIIIDGVNI<br>CTVGLHDVRSRFGIIPQEPVLFQGTVRSNIDPLGLYSEEEVWKVYILHYLVCFFCF
104.	GgABCC48	MT379765	MEDIWSMICGEYSGCSETGGKPYDFKFLKDIPSTCISHVFIIFFDVLMLIMLSFIMILKSWSRPFWSLVRYSKLQ<br>LVSAITNGSLGLFHLCLGIWVSEEMLRKTHKAFPLNFWLLELFQGFTWLLVSLTVSLQIKQLPRAWLWLFSIL<br>MFFVSFILCALSMSYATGSRELSLNAALDVLSFLGASLLLLCTYKACKCAEADREIGESLYAPLNSQFNEADPV<br>SNRTTPFAKAGFFSKMWFWWLNPLMKRGQEKTLQDEDIPKLRNSDRAESCYLSFAEPFNRQKQNEASSHSSV<br>LWTIILCHRREILVTGFFALLKVLTISSGPLLLNAFILVAEGNESFKYEGYVLAISLFFVKIIESLSQRQWYFHSRL<br>VGMKVRSLLTAAIYKKALRLSNSARLVHSGGEIMNYVTVDAYRIGEFPFWFHQTWTTILQLCIALVILFHAVG<br>LATIASLVVIVITVLCNTPLAKLQHKFQSQLMVAQDERLKASSEALVNMKVLKLYAWETHFRNAIENLRNVE<br>LKVLSSLQSKKAYNIFLFWTSPMLVSAASFLACYFLEIPLHASNVFTFVATLRLVQDPITAIPDVVGVIIQAKVA<br>FARIVKFLEAPELQNSNFRKRGISDNLRGSIFIKSADFSWEGSGSKPTLRSINLEVRHGQKVAICGEVGSGKSTL<br>LTTILGEVPNTKGTVSFCSFYAHTIYTLLNTLIGNNMV
105.	GgABCC49	MT379766	MAISDSLLGTANVVFFYGILIWLLVDSLRLSQRNHVAFNLKKRGPTVFATVTVLSSAVISVLNVAFAFYTYSTR<br>RIIGYNSVSLAVTWVLATTVSLYSVKNNTLGENSRFPLVLILWWVFACIVDMFSVSLKLVKINLESLNLWFFLS<br>EENNIVDAVSLPLLLLLCFNALPNVCEREQSEMEQRLLQKEMESSVEEDEDEEAFTKASRWSKLTFRWLNPIF<br>SRGRIQKLEHAHIPSVPRSEAAESASSMLEESLRKQKLEGGSLVKAITHSIWKSLALNAVLAGVNTIAAYIGPL<br>LISSFVNFLLSNDNNNSSIQHGLILAFVFFLSKTVESLSQRQWYFGAQRIGIRVRAALMALVYSKSLMIKCAGP<br>THGKIINLINVDVDRIGDFCWYIHGVWLLPVQVILALVILYINLGCAPSIAALTVTILVMVFNTPLANMQENLH<br>SKIMEAKDSRIKMTSETMKNIRILKLHSWESTFLQKLLQLRDTERRWLKKYLYTCSAVATLFWASPTLVSVAT<br>FGACILVKTELTAATVLSALATFRILQEPIYNLPELISMITQTKVSIDRIQEFIKEEDQNQFMNRHAPHTSAIAIEI<br>KPGEYAWDANDQTHKKPTIRITEKIMIKKGQKVAVCGSVGSGKSSFLCTMLGEIPLVSGAVTKVYGTRSYVP<br>QSPWIQSGTVRENILFGKQMQKDFYENVLDGCALHQDINTWSDGDLTLVEERGINLSGGQKQRIQLARAVYN<br>DSDIYFLDDPFSAVDAHTGSHLFKKCLMKLLCDKTVVYATHQLEFLEAADLILVMKDGKIVESGRYADLIAC<br>PSSELVQQMAAHKETITQIPCHKDDFISCRPCQKNPTEIAEENIQEIIMDWKRTREEEAMTGRVKWSVYSTFVT<br>LAYRGVLVPVILLCQILFQVMQMGSNYWISWATEQKGRVDNRELMGTFVLLSGGSSIFIMGRTVLMATVAVE<br>TAQRLFHGMITSVFRAPVSFFDTTPSSRILSRVSLAIILFLIRLLLSVLLFKSQPFSL
106.	GgABCC50	MT379767	MAISDSLLGTANVVFFYGILIWLLVDSLRLSQRNHVAFNLKKRGPTVFATVTVLSSAVISVLNVAFAFYTYSTR

			RIIGYNSVSLAVTWVLATTVSLYSVKNNTLGENSRFPLVLILWWVFACIVDMFSVSLKLVKINLESLNLWFFLS<br>EENNIVDAVSLPLLLLLCFNALPNVCEREQSEMEQRLLQKEMESSVEEDEDEEAFTKASRWSKLTFRWLNPIF<br>SRGRIQKLEHAHIPSVPRSEAAESASSMLEESLRKQKLEGGSLVKAITHSIWKSLALNAVLAGVNTIAAYIGPL<br>LISSFVNFLLSNDNNNSSIQHGLILAFVFFLSKTVESLSQRQWYFGAQRIGIRVRAALMALVYSKSLMIKCAGP<br>THGKIINLINVDVDRIGDFCWYIHGVWLLPVQVILALVILYINLGCAPSIAALTVTILVMVFNTPLANMQENLH<br>SKIMEAKDSRIKMTSETMKNIRILKLHSWESTFLQKLLQLRDTERRWLKKYLYTCSAVATLFWASPTLVSVAT<br>FGACILVKTELTAATVLSALATFRILQEPIYNLPELISMITQTKVSIDRIQEFIKEEDQNQFMNRHAPHTSAIAIEI<br>KPGEYAWDANDQTHKKPTIRITEKIMIKKGQKVAVCGSVGSGKSSFLCTMLGEIPLVSGAVTKVYGTRSYVP<br>QSPWIQSGTVRENILFGKQMQKDFYENVLDGCALHQDINTWSDGDLTLVEERGINLSGGQKQRIQLARAVYN<br>DSDIYFLDDPFSAVDAHTGSHLFKKCLMKLLCDKTVVYATHQLEFLEAADLILVMKDGKIVESGRYADLIAC<br>PSSELVQQMAAHKETITQIPCHKDDFISCRPCQKNPTEIAEENIQEIIMDWKRTREEEAMTGRVKWSVYSTFVT<br>LAYRGVLVPVILLCQILFQVMQMGSNYWISWATEQKGRVDNRELMGTFVLLSGGSSIFIMGRTVLMATVAVE<br>TAQRLFHGMITSVFRAPVSFFDTTPSSRILSRSSTDQSTVDTDIPYRLAGLVFALIQLLSIIMLMSQVAWQVILLF<br>FVVLAISIWYQAYYITTARELARMVGIRKAPILHHFSESIAGAATIRCFNQEQIFLKKVMALIDDYSRVAFHNY<br>ATMEWLSVRINFLFNLVFYVVLVILVTLPRSAIDPSLAGLVATYGLNLNVLQAWVIWNLCNVENKMISVERIL<br>QFSSIPSEAPLIIQDCRPDPEWPRDGKIELHNLHIQYDPAAPMVLKGVTCVFPGQKKIGIVGRTGSGKSTLVQAL<br>FRVVEPVEGWILIDGVHISKIGLQDLRSKLGIIPQDPTLFLGTVRTNLDPLEQHKDQELWEVLSKCHLAEIVKQ<br>DPRLLDAPVAENGENWSVGQRQLVCLARLLLKKRRILVLDEATASIDTVTDNLIQKTIREETSGCTVITVAHRI<br>PTVIDNDLVLVLDEGTRRLGMQYAHQFILS
107.	GgABCC51	MT379768	MAISDSLLGTANVVFFYGILIWLLVDSLRLSQRNHVAFNLKKRGPTVFATVTVLSSAVISVLNVAFAFYTYSTR<br>RIIGYNSVSLAVTWVLATTVSLYSVKNNTLGENSRFPLVLILWWVFACIVDMFSVSLKLVKINLESLNLWFFLS<br>EENNIVDAVSLPLLLLLCFNALPNVCEREQSEMEQRLLQKEMESSVEEDEDEEAFTKASRWSKLTFRWLNPIF<br>SRGRIQKLEHAHIPSVPRSEAAESASSMLEESLRKQKLEGGSLVKAITHSIWKSLALNAVLAGVNTIAAYIGPL<br>LISSFVNFLLSNDNNNSSIQHGLILAFVFFLSKTVESLSQRQWYFGAQRIGIRVRAALMALVYSKSLMIKCAGP<br>THGKIINLINVDVDRIGDFCWYIHGVWLLPVQVILALVILYINLGCAPSIAALTVTILVMVFNTPLANMQENLH<br>SKIMEAKDSRIKMTSETMKNIRILKLHSWESTFLQKLLQLRDTERRWLKKYLYTCSAVATLFWASPTLVSVAT<br>FGACILVKTELTAATVLSALATFRILQEPIYNLPELISMITQTKVSIDRIQEFIKEEDQNQFMNRHAPHTSAIAIEI<br>KPGEYAWDANDQTHKKPTIRITEKIMIKKGQKVAVCGSVGSGKSSFLCTMLGEIPLVSGAVTKVYGTRSYVP<br>QSPWIQSGTVRENILFGKQMQKDFYENVLDGCALHQDINTWSDGDLTLVEERGINLSGGQKQRIQLARAVYN<br>DSDIYFLDDPFSAVDAHTGSHLFKKCLMKLLCDKTVVYATHQLEFLEAADLILVMKDGKIVESGRYADLIAC<br>PSSELVQQMAAHKETITQIPCHKDDFISCRPCQKNPTEIAEENIQEIIMDWKRTREEEAMTGRVKWSVYSTFVT<br>LAYRGVLVPVILLCQILFQVMQMGSNYWISWATEQKGRVDNRELMGTFVLLSGGSSIFIMGRTVLMATVAVE<br>TAQRLFHGMITSVFRAPVSFFDTTPSSRILSRSSTDQSTVDTDIPYRLAGLVFALIQLLSIIMLMSQVAWQVILLF<br>FVVLAISIWYQVRTISIFDDVH
108.	GgABCC52	MT379769	MFSVSLKLVKINLESLNLWFFLSEENNIVDAVSLPLLLLLCFNALPNVCEREQSEMEQRLLQKEMESSVEEDE<br>DEEAFTKASRWSKLTFRWLNPIFSRGRIQKLEHAHIPSVPRSEAAESASSMLEESLRKQKLEGGSLVKAITHSI<br>WKSLALNAVLAGVNTIAAYIGPLLISSFVNFLLSNDNNNSSIQHGLILAFVFFLSKTVESLSQRQWYFGAQRIGI<br>RVRAALMALVYSKSLMIKCAGPTHGKIINLINVDVDRIGDFCWYIHGVWLLPVQVILALVILYINLGCAPSIAA<br>LTVTILVMVFNTPLANMQENLHSKIMEAKDSRIKMTSETMKNIRILKLHSWESTFLQKLLQLRDTERRWLKK<br>YLYTCSAVATLFWASPTLVSVATFGACILVKTELTAATVLSALATFRILQEPIYNLPELISMITQTKVSIDRIQEF<br>IKEEDQNQFMNRHAPHTSAIAIEIKPGEYAWDANDQTHKKPTIRITEKIMIKKGQKVAVCGSVGSGKSSFLCT<br>MLGEIPLVSGAVTKVYGTRSYVPQSPWIQSGTVRENILFGKQMQKDFYENVLDGCALHQDINTWSDGDLTLV<br>EERGINLSGGQKQRIQLARAVYNDSDIYFLDDPFSAVDAHTGSHLFKKCLMKLLCDKTVVYATHQLEFLEAA<br>DLILVMKDGKIVESGRYADLIACPSSELVQQMAAHKETITQIPCHKDDFISCRPCQKNPTEIAEENIQEIIMDWK<br>RTREEEAMTGRVKWSVYSTFVTLAYRGVLVPVILLCQILFQVMQMGSNYWISWATEQKGRVDNRELMGTF<br>VLLSGGSSIFIMGRTVLMATVAVETAQRLFHGMITSVFRAPVSFFDTTPSSRILSRSSTDQSTVDTDIPYRLAGL<br>VFALIQLLSIIMLMSQVAWQVILLFFVVLAISIWYQAYYITTARELARMVGIRKAPILHHFSESIAGAATIRCFN<br>QEQIFLKKVMALIDDYSRVAFHNYATMEWLSVRINFLFNLVFYVVLVILVTLPRSAIDPSLAGLVATYGLNLN<br>VLQAWVIWNLCNVENKMISVERILQFSSIPSEAPLIIQDCRPDPEWPRDGKIELHNLHIQYDPAAPMVLKGVTC<br>VFPGQKKIGIVGRTGSGKSTLVQALFRVVEPVEGWILIDGVHISKIGLQDLRSKLGIIPQDPTLFLGTVRTNLDP<br>LEQHKDQELWEVLSKCHLAEIVKQDPRLLDAPGIN
109.	GgABCC53	MT379770	MFSVSLKLVKINLESLNLWFFLSEENNIVDAVSLPLLLLLCFNALPNVCEREQSEMEQRLLQKEMESSVEEDE<br>DEEAFTKASRWSKLTFRWLNPIFSRGRIQKLEHAHIPSVPRSEAAESASSMLEESLRKQKLEGGSLVKAITHSI<br>WKSLALNAVLAGVNTIAAYIGPLLISSFVNFLLSNDNNNSSIQHGLILAFVFFLSKTVESLSQRQWYFGAQRIGI<br>RVRAALMALVYSKSLMIKCAGPTHGKIINLINVDVDRIGDFCWYIHGVWLLPVQVILALVILYINLGCAPSIAA<br>LTVTILVMVFNTPLANMQENLHSKIMEAKDSRIKMTSETMKNIRILKLHSWESTFLQKLLQLRDTERRWLKK<br>YLYTCSAVATLFWASPTLVSVATFGACILVKTELTAATVLSALATFRILQEPIYNLPELISMITQTKVSIDRIQEF<br>IKEEDQNQFMNRHAPHTSAIAIEIKPGEYAWDANDQTHKKPTIRITEKIMIKKGQKVAVCGSVGSGKSSFLCT<br>MLGEIPLVSGAVTKVYGTRSYVPQSPWIQSGTVRENILFGKQMQKDFYENVLDGCALHQDINTWSDGDLTLV<br>EERGINLSGGQKQRIQLARAVYNDSDIYFLDDPFSAVDAHTGSHLFKKCLMKLLCDKTVVYATHQLEFLEAA<br>DLILVMKDGKIVESGRYADLIACPSSELVQQMAAHKETITQIPCHKDDFISCRPCQKNPTEIAEENIQEIIMDWK<br>RTREEEAMTGRVKWSVYSTFVTLAYRGVLVPVILLCQILFQVMQMGSNYWISWATEQKGRVDNRELMGTF<br>VLLSGGSSIFIMGRTVLMATVAVETAQRLFHGMITSVFRAPVSFFDTTPSSRILSRSSTDQSTVDTDIPYRLAGL<br>VFALIQLLSIIMLMSQVAWQVILLFFVVLAISIWYQAYYITTARELARMVGIRKAPILHHFSESIAGAATIRCFN<br>QEQIFLKKVMALIDDYSRVAFHNYATMEWLSVRINFLFNLVFYVVLVILVTLPRSAIDPSLAGLVATYGLNLN<br>VLQAWVIWNLCNVENKMISVERILQFSSIPSEAPLIIQDCRPDPEWPRDGKIELHNLHIQYDPAAPMVLKGVTC<br>VFPGQKKIGIVGRTGSGKSTLVQALFRVVEPVEGWILIDGVHISKIGLQDLRSKLGIIPQDPTLFLGTVRTNLDP<br>LEQHKDQELWEVSI
110.	GgABCC54	MT379771	MIKEQGTFEELSKRGHLFQKLMENAGKMEQEADSDKDRDCKDKANTLPLNNEEIVELPNDASYEKKGKLRK<br>SVLVKKEDRETGVVSWKVIMRYKSALGGLWVVSVLFACYTSTEALRISSSTWLSVWTAQDSTTASEAGYFLF<br>IYALFSFGQVSVTLANSYWLIISSLRAAKRLHDAMLDRILRAPMVFFQTNPVGRIINRFAKDTGDIDTNVFSLV<br>NMFLGQVWQLLSTFVLIGTVSTISLWAIMPLLIFFYAAYIYYQSTAREVKRLDSITRSPVYAHFGEALNGLSSIR

			AYKAYDRMEHINGKFMDNNIRFGLVNISSNRWLTIRLETLGGLMIWLIATFAVLQNSRAENQALIASTMGLLLSYTLNITNLLSGVLRQASRAENSLNSVERVDTYINLETEAPGVIETNRPPPGWPTSGSIEFENVVLTYRPELPPVLHGLSFTVPPTEKIGVVGRTGAGKSSMLNALFRIVELQRGRIIIDGCDISTFGLADLRSVLTIIPQSPVLFSGMHHNQKSCSMSYLDNQA
111.	GgABCF1	MT379772	MYCFSLFLFDISTMFWLFFITSIRMFNIWSEYLQIHRFFQKKFADLEACVWLEENLKKFDRILVVISHSQDFLNGVCTNIIHMQNKKLKPYTGNYDQYVQTRSELEENQMKQYKWEQEQIASMKEYIARFGHGSAKLARQAQSKEKTLAKMERGGLTEKVARDKVLVFRFVDVGKLPPPVLQFVEVSFGYTPDNLIYKNIDFGVDLDSRIALVGPNGAGKSTLLKLMTGELSPLDGMVRRHNHLRIAQFHQHLAEKLDMELSALQYMIKEYPGNEEEKMRAAIGKFGLSGKAQVMPMKNLSDGQRSRVIFAWLAFRQPQLLLLDEPTNHLDIETIDSLAEALNEWDGGLVLVSHDFRLINQVAHEIWVCADQTVTRWEGDIMMFKQHLRAKAGLSD
112.	GgABCF2	MT379773	MDIHHLSREIEASDISALDAVISCDEERLKLEKEAEALAAQDDGGGEALERIYERLDAMDASTAEKRAAEILFGLGFNKQMQAKKTRDFSGGWRMRIALARALFMNPTILLLDEPTNHLDLEACVWLEENLKKFDRILVVISHSQDFLNGVCTNIIHMQNKKLKPYTGNYDQYVQTRSELEENQMKQYKWEQEQIASMKEYIARFGHGSAKLARQAQSKEKTLAKMERGGLTEKVARDKVLVFRFVDVGKLPPPVLQFVEVSFGYTPDNLIYKNIDFGVDLDSRIALVGPNGAGKSTLLKLMTGELSPLDGMVRRHNHLRIAQFHQHLAEKLDMELSALQYMIKEYPGNEEEKMRAAIGKFGLSGKAQVMPMKNLSDGQRSRVIFAWLAFRQPQLLLLDEPTNHLDIETIDSLAEALNEWDGGLVLVSHDFRLINQVAHEIWVCADQTVTRWEGDIMMFKQHLRAKAGLSD
113.	GgABCF3	MT379774	MCSLHVFYYSCQMYLQVVTDIIHLQNQKLTTYRGNYDTFERTREEQIKNKQKAIEAHDRARSHMQSFIDKFRYNAKRASLVQSRIKALDRMGHVDEIVNDPDYKFEFPTPDDRPGPPIISFSDASFGYPGGPILFKNLNFGIDLDSRIAMVGPNGIGKSTILKLIAGELQPSSGTVFRSAKVRIAVFSQHHVDGLDLSSNPLLYMMRCYPGVPEQKLRAHLGSFGVTGNLALQPMYTLSGGQKSRVAFAKITFKKPHIILLDEPSNHLDLDAVEALIQGLVLFQGGILMVSHDEHLISGSVEELWAVSEGRVTPFHGTFQDYKKILQS
114.	GgABCF4	MT379775	MQKGHHLFNLESRYLLLSVYLLINQMPKIWVHICMLYHAFFVLSIYVLFPGKIFQALDRMGHVDEIVNDPDYKFEFPTPDDRPGPPIISFSDASFGYPGGPILFKNLNFGIDLDSRIAMVGPNGIGKSTILKLIAGELQPSSGTVFRSAKVRIAVFSQHHVDGLDLSSNPLLYMMRCYPGVPEQKLRAHLGSFGVTGNLALQPMYTLSGGQKSRVAFAKITFKKPHIILLDEPSNHLDLDAVEALIQGLVLFQGGILMVSHDEHLISGSVEELWAVSEGRVTPFHGTFQDYKKILQS
115.	GgABCF5	MT379776	MIVQDLICSPSLTSSAIMQKGHHLFNLESRYLLLSVYLLINQMPKIWVHICMLYHAFFVLSIYVLFPGKIFQALDRMGHVDEIVNDPDYKFEFPTPDDRPGPPIISFSDASFGYPGGPILFKNLNFGIDLDSRIAMVGPNGIGKSTILKLIAGELQPSSGTVFRSAKVRIAVFSQHHVDGLDLSSNPLLYMMRCYPGVPEQKLRAHLGSFGVTGNLALQPMYTLSGGQKSRVAFAKITFKKPHIILLDEPSNHLDLDAVEALIQGLVLFQGGILMVSHDEHLISGSVEELWAVSEGRVTPFHGTFQDYKKILQS
116.	GgABCF6	MT379777	MVGPNGIGKSTILKLIAGELQPSSGTVFRSAKVRIAVFSQHHVDGLDLSSNPLLYMMRCYPGVPEQKLRAHLGSFGVTGNLALQPMYTLSGGQKSRVAFAKITFKKPHIILLDEPSNHLDLDAVEALIQGLVLFQGGILMVSHDEHLISGSVEELWAVSEGRVTPFHGTFQDYKKILQS
117.	GgABCF7	MT379778	MGRKKTEDAGTSAKAKASSKDPPKKEKFSVSAMLAGMDEKPDKPKKPSSSSSKPKPKSAPKASTYTDGIDLPPSDDEDEYILEEEQEQNVSKRSSQQQKPLEVSIADKELKKREKKDLLAAHAVEQAKKEALRDDHDAFTVVIGSRTSVLDGEDDVDANVKDITIDNFSVSARGKELLKNASVRISHGKRYGLVGPNGKGKSTLLKLLAWRKIPVPKNIDVLLVEQEVVGDDKTALEAVVSANDELVKIRQEVADLQNAASIEEGADKDDDNNQGEDAGEKLAELYEQLQLMGSDAAEAQASKILAGLGFTKDMQGRPTKSFSGGWRMRISLARALFVQPTLLLLDEPTNHLDLRAVLWLEEYLCRWKKTLVVVSHDRDFLNTVCTEIIHLHDLKLHFYRGNFDDFESGYEQRRREMNKKYEIYDKQLKAAKRTGNRAQQEKVKDRAKFAAAKEASKSKGKGRVDEDETQPEVPQKWRDYSVEFHFPEPTELTPPLLQLIEVSFSYPNREDFRLSNVDVGIDMGTRVAIVGPNGAGKSTLLNLLAGDLVPTEGEVRRSQKLRIGRYSQHFVDLLTMDETPVQYLLRLHPDQEGLSKQEAVRAKLGKFGLPSHNHLTPIAKLSGGQKSRVVFTSISMSKPHILLLDEPTNHLDMQSIDALADALDEFTGGVVLVSHDSRLISRVCDDEERSQIWVVEDGTVRTFPGTFEDYKDDLLREIKAEVDD
118.	GgABCF8	MT379779	MDFTAKLHHLDLTGAVILHARKPSTQQPRPYLLPRYAARPNNINNTHLIYPNRLFSGPKRPNYSPPRLSAVAVDDDTSLAQDDIESLFSSDPSVDERQLVANKPSNTGASSVSSGVKLENISKTYKGVTVLNDVSWEVKKGEKVGLVGVNGAGKTTQMRIIAGQEEPDSGNVIKARPNMRIAFLSQEFEVSLSRTVREEFMSAFKEEMEVAAKLEKVQKALEGAVNDLELMGRLLDEFDLLQRRAQNVNLDVVDAKISKLMPELGFAPEDSDRLVASFSGGWQMRMCLGKILLQEPDLLLLDEPTNHLDLDTIEWLEDYLNKQDVPMVIISHDRAFLDQLCTKIVETDMGVARTYEGNYSQFVVSKATWIEAQYAAWEKQQKEIEQTKDLINRLGAGANSGRASSAEKKLERLQGEELVEKPYERKQMKIRFPERGRSGRSVVTIKNLEFGFEDTKLFDKASLTIERGEKIAIIGPNGCGKSTLLKLIMGLEKPIGGEVMLGEHNVLPNYFEQNQAEALDLDKTVLQTVEEAAEDWRIDDIKGLLGRCNFKSDMLDRKVSLLSGGEKARLAFCKFMVKPSTLLVLDEPTNHLDIPSKEMLEEAISEYEGTVITVSHDRYFIKQIVNRVIEVKDGTLQDYTGDYNYYLEKNLEARERELEREAELEDKAPKVKAKSKMSKAEKEARKKQKMQAFQAAKQKSKGAKNAKRWN
119.	GgABCF9	MT379780	MELAAKLHHLTATGTIILDARKPALRSLSNRHRFFSPKTPNFDAATCRLSAVDAAETTTTQDKNDNDIASLFSDTSTVEHDRKRGNKQSNTHNNKTGASSVSSGVKLENISKTYKGVTVLKDVSWEVKKGEKVGLVGVNGAGKTTQMRIIAGLEDPDSGNVIKAKPNMKVAFLSQEFEVSQSRTVREEFLSAFKEEMEVSGKLERVQKALEGSVDDLELMGRLLDEFDLLQRKGQALELDQVDAKVDKLMPELGFAPEDSDRLVASFSGGWQMRMSLGKILLQDPDLLLLDEPTNHLDLDTIEWLEYYLNEQDVPMVIISHDRAFLDQLCTKIVETDMGVSRTFEGNYSQYVVSKAAWVEAQNAAWEKQQKEIEQTKGLINRLGAGANSGRASSAEKKLERLQEEELVEKPFQRKQMRIRFPERGRSGRSVVTIKNLEYGFEDTVLFKKANLTIERGEKIAIVGPNGCGKSTLLKLIMGLEKPIGGEVMLGEHNVLPNYFEQNQAEALDLDKTVLQTVEEVAEDWRIDDIKGLLGRCNFKADMLDRKVSLLSGGEKARLAFCKFMVKPSTLLVLDEPTNHLDIPSKEMLEEAITEYQGTVITVSHDRYFIKQIVNRVIEIKDGTVQNYAGDYNYYLEKNLDARSRELEREADLEEKAPKVKAKSKMSKAEKEARKKQKMQAFQAAKQKSKSSKNAKRWN
120.	GgABCF10	MT379781	MELAAKLHHLTATGTIILDARKPALRSLSNRHRFFSPKTPNFDAATCRLSAVDAAETTTTQDKNDNDIASLFSDTSTVEHDRKRGNKQSNTHNNKTGASSVSSGVKLENISKTYKGVTVLKDVSWEVKKGEKVGLVGVNGAGKTTQMRIIAGLEDPDSGNVIKAKPNMKVAFLSQEFEVSQSRTVREEFLSAFKEEMEVSGKLERVQKALEGSVDDLELMGRLLDEFDLLQRKGQALELDQVDAKVDKLMPELGFAPEDSDRLVASFSGGWQMRMSLGKILLQDPDLLLLDEPTNHLDLDTIEWLEYYLNEQDVPMVIISHDRAFLDQLCTKIVETDMGVSRTFEGNYSQYVVSKAAWV

			EAQNAAWEKQQKEIEQTKGLINRLGAGANSGRASSAEKKLERLQEEELVEKPFQRKQMRIRFPERGRSGRSVVTIKNLEYGFEDTVLFKKANLTIERGEKIAIVGPNGCGKSTLLKLIMGLEKPIGGEVMLGEHNVLPNYFEQNQAEALDLDKTVLQTVEEVAEDWRIDDIKGLLGRCNFKADMLDRKVSLLSGGEKARLAFCKFMVKPSTLLVLDEPTNHLDIPSKEMLEEAITEYQGTVITVSHDRYFIKQIVNRVIEIKDGTVQNYAGDYNVSGIQPCLCPLSMHLRFDGMH
121.	GgABCF11	MT379782	MELAAKLHHLTATGTIILDARKPALRSLSNRHRFFSPKTPNFDAATCRLSAVDAAETTTTQDKNDNDIASLFSDTSTVEHDRKRGNKQSNTHNNKTGASSVSSGVKLENISKTYKGVTVLKDVSWEVKKGEKVGLVGVNGAGKTTQMRIIAGLEDPDSGNVIKAKPNMKVAFLSQEFEVSQSRTVREEFLSAFKEEMEVSGKLERVQKALEGSVDDLELMGRLLDEFDLLQRKGQALELDQVDAKVDKLMPELGFAPEDSDRLVASFSGGWQMRMSLGKILLQDPDLLLLDEPTNHLDLDTIEWLEYYLNEQDVPMVIISHDRAFLDQLCTKIVETDMGVSRTFEGNYSQYVVSKAAWVEAQNAAWEKQQKEIEQTKGLINRLGAGANSGRASSAEKKLERLQEEELVEKPFQRKQMRIRFPERGRSGRSVVTIKNLEYGFEDTVLFKKANLTIERGEKIAIVGPNGCGKSTLLKLIMGLEKPIGGEVMLGEHNVLPNYFEQNQAEALDLDKTVLQTVEEVAEDWRIDDIKGLLGRCNFKADMLDRKVSLLSGGEKVNL
122.	GgABCF12	MT379783	MDFTAKLHHLDLTGAVILHARKPSTQQPRPYLLPRYAARPNNINNTHLIYPNRLFSGPKRPNYSPPRLSAVAVDDDTSLAQDDIESLFSSDPSVDERQLVANKPSNTGASSVSSGVKLENISKTYKGVTVLNDVSWEVKKGEKVGLVGVNGAGKTTQMRIIAGQEEPDSGNVIKARPNMRIAFLSQEFEVSLSRTVREEFMSAFKEEMEVAAKLEKVQKALEGAVNDLELMGRLLDEFDLLQRRAQNVNLDVVDAKISKLMPELGFAPEDSDRLVASFSGGWQMRMCLGKILLQEPDLLLLDEPTNHLDLDTIEWLEDYLNKQDVPMVIISHDRAFLDQLCTKIVETDMGVARTYEGNYSQFVVSKATWIEAQYAAWEKQQKEIEQTKDLINRLGAGANSGRASSAEKVYVVN
123.	GgABCF13	MT379784	MKIRFPERGRSGRSVVTIKNLEFGFEDTKLFDKASLTIERGEKIAIIGPNGCGKSTLLKLIMGLEKPIGGEVMLGEHNVLPNYFEQNQAEALDLDKTVLQTVEEAAEDWRIDDIKGLLGRCNFKSDMLDRKVSLLSGGEKARLAFCKFMVKPSTLLVLDEPTNHLDIPSKEMLEEAISEYEGTVITVSHDRYFIKQIVNRVIEVKDGTLQDYTGDYNYYLEKNLEARERELEREAELEDKAPKVKAKSKMSKAEKEARKKQKMQAFQAAKQKSKGAKNAKRWN
124.	GgABCG1	MT379785	MSNRIVAENVLDDETTTNYDTIELGDVSSRRAAGEMPTLGQLLKHVGDVQKEASGDGSETPVHHALDIAGIEPRSLPFVLSFSNLTYSVKARRKFSLTSIFPCRRRLRGTAVVEAPAVGESVSTRTKTLLNDISGEARDGEIMAVLGASGSGKSTLIDALANRIAKGSLKGKVALNGEALESRLLKVISAYVMQDDLLFPMLTVEETLTYAAEFRLPRTLSSSKKKARVQALIDQLGLRNAATTVIGDEGHRGVSGGERRRVSIGIDIIHDPILLFLDEPTSGLDSTSAFMVVKVLQRIAQSGSIVIMSIHQPSYRILSLLDRMIFLSRGQTVYSGSPMQLPSFFAEFGHPIPETENRTEFALDLIRELEGSPGGTRSLVEFNKSWQSMAKKHENDAVSEQNGMSLKEAISASISRGKLVSGAAAATTNGTSQTTSSSMVPSFANSFWRETATLSKRSMRNSRRMPELFGIRLGTVMVTGIILATVFWNLDNSPKGVQERLGFFAFAMSTTYYTTADALPVFLQERYIFMRETAYNAYRKSSYLVSHALVALPALVFLSLAFAATTFWAVGLDGGFSGFLFYFLIILASFWAGNSFVSFLSGVVPHVMLGYTIVVAILAYFLLFSGFFINRDRIPGYWIWFHYLSLVKYPYEGVLQNEFEDSSKCFVKGVQMFDNTPLVDAPYELKLKLLDSISGTLGVNITASTCVTTGADVLSQNGVTQLSKWNCLWITVAWGFFFRFLFYVSLLVGSKNKRR
125.	GgABCG2	MT379786	MTHAMQNQKPNNPSRIRMSRLAPTPPNPNPVAEDRLSFFNQSMELIQLPNAPCRTRSHLSPTLGQLLKRVEDAQNNTTTEPPHHVLDLSSSSSTTTTLHSHPFFLSFTNLTYSVKLRRKMALSPCFSSSTHSSHPENEDAGAGAKPNRTKILLNGISGEARDGEIMAVLGASGSGKSTLIDALADRISKESLKGTVTLNGDVLESRLQKVISAYVMQDDLLFPMLTVEETLMFSAEFRLPRSLSKSKKKSRVQALIDQLGLRTAAKTVIGDEGHRGVSGGERRRVSIGIDIIHDPIILFLDEPTSGLDSTSAFMVVKVLQRIAQSGSIVIMSVHQPSYRILGLLDRLIFLSHGQTVFSGSPTSLPDFFSEFGHPIPENENRTEFALDLIRELEEAPGGTKSLVEFNKSWQLKNNNNNNTACEKTLNGGPKVCLKDAISASISRGKLVSGATSSNSTASSVPTFANPFWIEMAVIAKRSVTNSRRMPELFGIRLGAVLVTGVILATIFWRLDNSPKGVQERLGFFAFAMSTTFYTCAEAIPVFLQERYIFMRETAYNAYRRSSYVLAHSLISLPSLIFLSFAFAVTTFWSVGLAGGTSGFLFYFFTIFVSFWAGSSFVTFLSGVVSHVMLGFTVVVAILAYFLLFSGFFISRDRIPPYWLWFHYLSLVKYPYEGVLQNEFGVMPPTCFVRGIQMFDNTPLGNVPEFLKVELLKSMSGTLGMNITASTCVVTGADILKQQGITQLTKWNCLLVTIAWGFFFRFLFYLTLLCGSKNKRS
126.	GgABCG3	MT379787	MMERGNSTTTSSATAVLGLGRTKSEQLVESSMMMVKSSPASSEHSAEGSSTTVSRKSSRRQGNKNTHIRKSRSAQMKIEVDELCNTGAALSRASSASLGLSFSFTGFTMPPETSDSKPFSDDDFILEDIEAGTRTKFQTEPTLPIYLKFTDVTYKVVIKGMTTSEEKDILNGITGSVNPGEVLALMGPSGSGKTTLLNLLGGRLSQPTIGGSITYNDQSYSKFLKSRIGFVTQDDVLFPHLTVRETLTYAARLRLPQTFTKEQKEKRALDVIYELGLERCQDTMIGGSFVRGVSGGERKRVCIGNEIIINPSILFLDEPTSGLDSASSLLLLRALRREALEGVNICMVVHQPSYALFKMFDDLVLLGKGGLTVYHGSAKKVEEYFSGLGINVPERINPPDYYIDILEGIAVPGGSAGLSYKELPVRWMLHNGYLIPLDMRQNAAQFDMSQSVNSANEIDSNGYGHVGKTFAGELWQDVRNNVQLQGEKIRFNFLKSRDLSDRKTPGVFKQYKYFLIRAAKQRLREARIQAIDYLILLLAGACLGSITKASDQTFGAASYTYTVIAVSLLCKIAALRSFSLDKLHYWRESDSGMSSLAYFLSKDTIDHFNTVIKPVVYLSMFYFFTNPRSTFADNYVVLLCLVYCVTGIAYALSIFFEPGAAQLWSVLLPVVLTLIATQPKDSKVLKVLANLCYSKWALQALVIANAERYQGVWLISRCGSLLKSSYNLHDWSLCVSILILIGVIGRAIAFFCMVTFQKK
127.	GgABCG4	MT379788	MCVAVEDIEAGTRTKFQTEPTLPIYLKFTDVTYKVVIKGMTTSEEKDILNGITGSVNPGEVLALMGPSGSGKTTLLNLLGGRLSQPTIGGSITYNDQSYSKFLKSRIGFVTQDDVLFPHLTVRETLTYAARLRLPQTFTKEQKEKRALDVIYELGLERCQDTMIGGSFVRGVSGGERKRVCIGNEIIINPSILFLDEPTSGLDSASSLLLLRALRREALEGVNICMVVHQPSYALFKMFDDLVLLGKGGLTVYHGSAKKVEEYFSGLGINVPERINPPDYYIDILEGIAVPGGSAGLSYKELPVRWMLHNGYLIPLDMRQNAAQFDMSQSVNSANEIDSNGYGHVGKTFAGELWQDVRNNVQLQGEKIRFNFLKSRDLSDRKTPGVFKQYKYFLIRAAKQRLREARIQAIDYLILLLAGACLGSITKASDQTFGAASYTYTVIAVSLLCKIAALRSFSLDKLHYWRESDSGMSSLAYFLSKDTIDHFNTVIKPVVYLSMFYFFTNPRSTFADNYVVLLCLVYCVTGIAYALSIFFEPGAAQLWSVLLPVVLTLIATQPKDSKVLKVLANLCYSKWALQALVIANAERYQGVWLISRCGSLLKSSYNLHDWSLCVSILILIGVIGRAIAFFCMVTFQKK
128.	GgABCG5	MT379789	MLAGPTKTLFMDEISTGLDSATTFQICKFMRQMVHIMDETMVISLLQPAPETFELFDDIILLSEGQIVYHGPRENVLEFFENMGFKCPARKGVADFLQEVTSKKDQQQYWSRRDEPYRYVSVPEFAQSFHSFHIGKQLATELRVPYDKSQTHPAALAKDNYGLSNWELLKACFSREWLLMKRDSFVYIFRTTQLAILSVFAFTMFLRIEMPVGTVQDGQKYFGALFFSLMNMMFNGSSEQATIINRLPVFYKQRDFMFYPAWAFGLSMWVLRIPISLVEPTIWIAFTYYTIGFAPAATRFFRQYLALVSVHNMALGLFRLIGAIGRTRVVANILSGLTYQVVYVLGGFIVAKNDIKPWMVWGNYISPLTYGQNAIVINEFLDERWSEPNTDPRIDAPTVGKVLLKARGFYTEDYWFWICIGALFGFSLLFNLLFILALTY

			LNPFGDSKAFLRDEDNKKNEKSTSTRHILEERRTGMVLPFQPLSLAFSCVSYYVDMPAEMKSQGVNEDRLQLLHDVSGAFRPGILTALMGVSGAGKTTLMDVLAGRKTGGYIEGSISISGYPKNQATFARVSGYCEQNDIHSPHVTVYESLLFSAWLRLPSDIKTQKRKMFVEEVMELIELKPIKDALVGLPGVNGLSTEQRKRLTIAVELVANPSIIFMDEPTSGLDARAAAIVMRTVRNTVDTGRTIVCTIHQPSIDIFEAFDELLLMKRGGQVIYAGPLGRHSHKLVEYFEAIPGVPKIKDGYNPATWMLEISSPSIETQLDVDFAEIYASSTLYQRNQELIKELSTPAPDSKDLYFPTKYSQSFFVQCKANFWKQHLSYWRHPQYNAVRFFMTIVIGVMFGLIFWNQAQEARKQQDLMNLLGAMYSAVLFLGATNATSVQPVVSIERSVFYRERAAGMYSALPYAFGQVAIEAIYNAIQTAIYTLILYSMMGFEWKATNFLWFYYYILMSFMYFTLYGMMLVALTPGHQVAAICMSFFFSFWNLFSGFIIPRMQIPVWWRWYYWASPVAWTLYGLITSQLGDKNVELEIPGAGSMGLKEFLKQNWGYDYDLLPAVAAAHVGWVLLFVFVFAYGIKFLNFQKR
129.	GgABCG6	MT379790	MLAGPTKTLFMDEISTGLDSATTFQICKFMRQMVHIMDETMVISLLQPAPETFELFDDIILLSEGQIVYHGPRENVLEFFENMGFKCPARKGVADFLQEVTSKKDQQQYWSRRDEPYRYVSVPEFAQSFHSFHIGKQLATELRVPYDKSQTHPAALAKDNYGLSNWELLKACFSREWLLMKRDSFVYIFRTTQLAILSVFAFTMFLRIEMPVGTVQDGQKYFGALFFSLMNMMFNGSSEQATIINRLPVFYKQRDFMFYPAWAFGLSMWVLRIPISLVEPTIWIAFTYYTIGFAPAATRFFRQYLALVSVHNMALGLFRLIGAIGRTRVVANILSGLTYQVVYVLGGFIVAKNDIKPWMVWGNYISPMTYGQNAIVINEFLDERWSEPNTDPRIDAPTVGKVLLKARGFYTEDYWFWICIGALFGFSLLFNLLFILALTYLNPFGDSKAFLRDEDNKKNEKSTSTRHILEERRTGMVLPFQPLSLAFSCVSYYVDMPAEMKSQGVNEDRLQLLHDVSGAFRPGILTALMGVSGAGKTTLMDVLAGRKTGGYIEGSISISGYPKNQATFARVSGYCEQNDIHSPHVTVYESLLFSAWLRLPSDIKTQKRKMFVEEVMELIELKPIKDALVGLPGVNGLSTEQRKRLTIAVELVANPSIIFMDEPTSGLDARAAAIVMRTVRNTVDTGRTIVCTIHQPSIDIFEAFDELLLMKRGGQIIYAGPLGYHSHKLIEYFEAIVGVPKIEAGYNPATWMLEISTPEIEAQLGIDFAEIYANSTLYRRNQELIKDLSAPTPSSKDLSFPTKYSQSFFVQWKACFWKQYWSYWRNPPYNIVRLLFTFSIGIMFGLIFWDKGKKFQKQQDLFNLLGAMYVAVTFLGTLNAMGVQPVVDMERIVLYRERAAGMYSVLAYAFGQVAVEVIYNIIQTAIYTLIIFSMMGFEWKDGKFFSLYYYMLMCLIYYTLFGMMIISLTPSYHIACIFGPFFMNTWNLFAGFVIPRMVSFMPF
130.	GgABCG7	MT379791	MKSQGVNEDRLQLLHDVSGAFRPGILTALMGVSGAGKTTLMDVLAGRKTGGYIEGSISISGYPKNQATFARVSGYCEQNDIHSPHVTVYESLLFSAWLRLPSDIKTQKRKMFVEEVMELIELKPIKDALVGLPGVNGLSTEQRKRLTIAVELVANPSIIFMDEPTSGLDARAAAIVMRTVRNTVDTGRTIVCTIHQPSIDIFEAFDELLLMKRGGQVIYAGPLGRHSHKLVEYFEAIPGVPKIKDGYNPATWMLEISSPSIETQLDVDFAEIYASSTLYQRNQELIKELSTPAPDSKDLYFPTKYSQSFFVQCKANFWKQHLSYWRHPQYNAVRFFMTIVIGVMFGLIFWNQAQEARKQQDLMNLLGAMYSAVLFLGATNATSVQPVVSIERSVFYRERAAGMYSALPYAFGQVAIEAIYNAIQTAIYTLILYSMMGFEWKATNFLWFYYYILMSFMYFTLYGMMLVALTPGHQVAAICMSFFFSFWNLFSGFIIPRMQIPVWWRWYYWASPVAWTLYGLITSQLGDKNVELEIPGAGSMGLKEFLKQNWGYDYDLLPAVAAAHVGWVLLFVFVFAYGIKFLNFQKR
131.	GgABCG8	MT379792	MCYLIYTTLLPHPIICVEKLSSTCAIQLFHNICRVGIRLPTVEVRFQNLNVEADSYVASRALPTLPNVALNISESALGIFGISTAKKTQLTILKNVSGIIKPSRMALLLGPPSSGKTTLLLALAGKLDPDLRVNGEITYNGHKLNEFVPKKTSAYISQNDAHLGEMTVKETLDFSARCQGIGTRYDLLAELARREKEAGIFPEAELDLFMKATAMEGTESSLITDYTLKILGLDICKDTIVGDEMQRGVSGGQKKRVTTGEMIVGPTKTLFMDEISTGLDSSTTYQIVKCFQQIVHLTEATIFMSLLQPAPETFDLFDDIILISEGQIVYQGPREHVIEFFESCGFRCPERKGTADFLQEVTSRKDQEQYWANRNIPYRYITVSEFANRFKQFHVGMQLQQELSVPFDKSTGHRAALVFKKYTVPTMGLFKACWDKEWLLIKRNSFVYIFKTVQICILAVISSTVFIRTKMHRRNEEDAAVYIGALLFSMIMNMFNGFAELPLTIQRLPVFYKHRDHLFHPPWTYTLPNFLLRIPISIFEAIVWVLITYYSIGFAPEASRFFKHLLLVFLVQQMAAGMFRVISGVCRTMIIANTGGALMLLLVFLLGGFILPKRDIPNWWVWAYWVSPLTYAFNAFSVNEMFAPRWSKPSSSGLTSLGEAALNNFDVFTEKQWYWIGAAGLLGFTILYNVLFTLALIYLNPVGKKQAIISEEEASEMEAGGDSKEEPRLVRPEPNRELPLRSLSSADGNNTREIAMQRMSSRGNPSGLRNVDPTLEPATGVAPKRGMVLPFQPLAMSFDSVNYYVDMPAEMREQGVTDDRLQLLREVTGAFRPGVLTALMGVSGAGKTTLMDVLAGRKTGGYIEGDVRISGFPKNQETFARISGYCEQTDIHSPQVTVLESVIYSAFLRLPREVNNEEKMKFVDEVMDLVELDNLKDAIVGLPGVTGLSTEQRKRLTIAVELVANPSIIFMDEPTSGLDARAAAIVMRTVRNTVDTGRTVVCTIHQPSIDIFEAFDELLLMKRGGQVIYSGPLGRNSHKIIEYFEAIQGVPEIKDKYNPATWMLEVSSIAAEVRLGMDFAEYYNSSSLYQRNKALVQELSTPPPEAKDLYFPTQFSQSTWKQFKSCIWKQWLTYWRSPDYNLVRFFFTLVAALLVGSVFWRVGKKRDSSADLTTIIGALYGSVFFVGVNNCQTVQPVVAIERTVFYRERAAGMYSALPYAIAQVLCEIPYVFFQTISYAFIVYAMVSFEWKVEKVLWFFFVSFFSFLYFTYYGMMTVSITPNHQVASIFGAAFYGVFNLFSGFFIARPKIPKWWVWYYWICPMAWTVYGLIVSQYRDITEGISVSGELNKIPIKQYIEDHYGFKPDFMGPVAAVLVAFTAFFAFVFAFSIKALNFQTR
132.	GgABCG9	MT379793	MPQNCSIAPKPEYCNSLSVEGPPEMTQPHDDSSVLPYPIQTNEQSLPKLIMYPITLKFEDLVYKVKIEEQKGVCWGSKWTCREKTILNGITGVVCPGEILAMLGPSGSGKTTLLTALGGRLSGKLSGKITYNNQPFSGSIKRRTGFVAQDDVLYPHLTVTETLVFTALLRLPKSLSRDEKVEHVERVITELGLTRCRSSMIGGPLLRGISGGEKRRVSIGQEMLINPSLLLLDEPTSGLDSTTALRILNTIKRLAAGGRTVVTTIHQPSSRLYYMFDKVVLLSEGCPIYYGPASTALDYFSSVGFSTCMTVNPADLLLDLANGIAPDSKHVTEQSEVLEQERKMVRESLISAYEKNIAPRLKAEVCSLEVNNYNNNTTKDACTRNHIKPEQWSNSWWHQFTVLLQRGVRERRHEAFNRLRIFQVISVALLGGLLWWHTPESHIQDRVALLFFFSVFWGFYPLYNAVFTFPQERRMLIKERSSGMYRLSSYFLARTIGDLPLELALPTAFVFIIYWMGGLKPDPATFILSLLVVLYSVVVSQSLGLALGAILMEIKQATTLASVTTLVFLIAGGYYIQQIPPFIVWLKYLSYSYYCYRLLLGVQYNENDYYECSKGQFCKVIEFPPIKSMGLNRMWVDVCIMALMLVGYRLVAYFALHRVR
133.	GgABCG10	MT379794	MQFEDLVYKVKIEEQKGVCWGSKWTCREKTILNGITGVVCPGEILAMLGPSGSGKTTLLTALGGRLSGKLSGKITYNNQPFSGSIKRRTGFVAQDDVLYPHLTVTETLVFTALLRLPKSLSRDEKVEHVERVITELGLTRCRSSMIGGPLLRGISGGEKRRVSIGQEMLINPSLLLLDEPTSGLDSTTALRILNTIKRLAAGGRTVVTTIHQPSSRLYYMFDKVVLLSEGCPIYYGPASTALDYFSSVGFSTCMTVNPADLLLDLANGIAPDSKHVTEQSEVLEQERKMVRESLISAYEKNIAPRLKAEVCSLEVNNYNNNTTKDACTRNHIKPEQWSNSWWHQFTVLLQRGVRERRHEAFNRLRIFQVISVALLGGLLWWHTPESHIQDRVALLFFFSVFWGFYPLYNAVFTFPQERRMLIKERSSGMYRLSSYFLARTIGDLPLELALPTAFVFIIYWMGGLKPDPATFILSLLVVLYSVVVSQSLGLALGAILMEIKQATTLASVTTLVFLIAGGYYIQQIPPFIVWLKYLSYSYYCYRLLLGVQYNENDYYECSKGQFCKVIEFPPIKSMGLNRMWVDVCIMALMLVGYRLVAYFALHRVR
134.	GgABCG11	MT379795	MTTKWGGLLKKNTKAEEKIILNGVTGMVQPGEILAILGPSGSGKTTLLTAFGGRLGGKLYGSITYNGKALSNAMKRNIGFVTQDDVLHPHLTVTETLVFSALLRLPSCFTKEEKIEHAKDVIAQIGLTKCKDSIIGGPMLRGVSGGE

			RKRISIGQEMLINPSLLFLDEPTSGLDSTTAQRIVSTL
135.	GgABCG12	MT379796	MPTPTATAAFGGVETPNGDSSNTHAPPKHGPRESRDHPPLLSSSYPITLKFMDVGYRLKIENKKGKGGCIKELFATHESSPSDQRSTVGERTILNGVTGIAYPGEILAILGPSGSGKSTLLNALAGRLHGNGLTGTVLANSSKLNKPILRRTGFVAQDDILYPHLTVRETLVFCSMLRLPRTLPRAAKVAAAESAIVELGLRKCEDTIIGNSFIRGVSGGERKRVSIAHEMLVDPALLILDEPTSGLDSTAAHRLVATLGSLAKKGKTVVTSVHQPSSRVYQMFDRVLVLSEGYCLYYGKGTDAMRYFESVGFAPSFPVNPADFLLDLANGTSTSFFLLLPSQTNYLFIFLTKQNRLIISLVTIV
136.	GgABCG13	MT379797	MDVGYRLKIENKKGKGGCIKELFATHESSPSDQRSTVGERTILNGVTGIAYPGEILAILGPSGSGKSTLLNALAGRLHGNGLTGTVLANSSKLNKPILRRTGFVAQDDILYPHLTVRETLVFCSMLRLPRTLPRAAKVAAAESAIVELGLRKCEDTIIGNSFIRGVSGGERKRVSIAHEMLVDPALLILDEPTSGLDSTAAHRLVATLGSLAKKGKTVVTSVHQPSSRVYQMFDRVLVLSEGYCLYYGKGTDAMRYFESVGFAPSFPVNPADFLLDLANGVCHVDGVSESRPNMKQNLVHSYNTVLGPKAKAACMDTANVPTKDTHPLRNSPSKERRYRDRVSFFDWFYQFSILLQRSLKERKYESFNTLRVFQVVAAALLAGLMWWHSDYRNIQDRLGLLFFISIFWGVFPSFNSVFAFPQERAIFMKERASGMYTLSSYFMARIVGDLPMELILPTVFLIITYWMGGLKPDLWAFLLTWLVVLGYVLVSQGLGLALGAAIMDAKQASTVAAVTMLAFVLTGGYYVHKVPACMAWIKYISTTFYCYRLLTRIQYGDGKKIAYLLGCYHGDMNGANCRFIEEDVVGQIGTVGSIGVLFFMFVFYRVLAYLALRRIKS
137.	GgABCG14	MT379798	MKASSVGGRKHSVNTDYSLKVLGLDICSDTIVGNDMLRGVSGGQRKRVTTGEMIVGPRKTLFMDEISTGLDSSTTYQIVKCIKNFVHQMDATVLMALLQPAPETFELFDDLVLLSEGHVVYEGPREDVLEFFQSIGFQLPPRKGIADFLQEVTSKKDQAKYWADHSKPYGFVSVREIAEAFRNSRFGSHMESLQAQPYDKSKCHTSALARNKFAVSKWEMTKACFQREILLIKRHSFLYIFRTFQVAFVGFVTCTIFLRTRLHPTDEAYGNLYLSALFFGLVHMMFNGFSELPLMITRLPVFYKQRDNLFYPAWAWSFTSWILRVPYSIIEAVIWTVVVYYTVGFAPAAGRFFRYMFLLFVVHQMAIGLFRMMAAIARDIVLANTFGAAALLIIFLLGGFIVPKAMIKPWWIWGYWLSPLTYGQRAITVNEFTATRWMKKSAIGYSTVGYNVLQLHSLPSDDYWYWAGVGILIVYAIFFNNMVTLALAYLNPLHKARTVIPLDDDSEKNSSRDGFAVSNLGHEMSTRTRSTRDNSTARGMILPFQPLTMTFHNVNYFVDMPKEIRKQGLPETRLQLLTDVSGVFSPGVLTALVGSSGAGKTTLMDVLAGRKTGGYIEGDIKISGYPKVQRTFARISGYVEQNDIHSPQVTIEESLWFSASHRLPKEISVDKRREFVEQVMKLVELDTLRDALVGMPGSSGLSTEQRKRLTIAVELVANPSIIFMDEPTSGLDARAAAIVMRTVRNTVDTGRTVVCTIHQPSIDIFEAFDELLLMKRGGRVIYGGKLGVQSQIMIDYFQRISGIPPIPSGYNPATWVLEVTTPAVEERIDADFADIYKKSDQYRGVEASIMQFQLPPAGSEPLKFDTMYSQNLLSQFFLCLWKQNLVYWRSPPYNAMRLFFTTVSALIFGTVFWDIGSKRASTQELFVVMGALYSASLFLGVNNASSVQPIVSIERTVFYREKAAGMYSPIAYAAAQGLVEIPYIAVQTIVFGVITYFMINFERTAGKFFLYLLFMFLTFTYFTFYGMMAVGLTSSQQLAAVISSAFYSLWNLLSGFLIPKSNIPGWWIWFYYICPVQWTLRGIITSQLGDVETKIVGPGFEGTVKEYLSASLGYENKINGFSSVGLSVIVLFGFVLLFFGSFAVSVKVLNFQKR
138.	GgABCG15	MT379799	MVTLALAYLNPLHKARTVIPLDDDSEKNSSRDGFAVSNLGHEMSTRTRSTRDNSTARGMILPFQPLTMTFHNVNYFVDMPKEIRKQGLPETRLQLLTDVSGVFSPGVLTALVGSSGAGKTTLMDVLAGRKTGGYIEGDIKISGYPKVQRTFARISGYVEQNDIHSPQVTIEESLWFSASHRLPKEISVDKRREFVEQVMKLVELDTLRDALVGMPGSSGLSTEQRKRLTIAVELVANPSIIFMDEPTSGLDARAAAIVMRTVRNTVDTGRTVVCTIHQPSIDIFEAFDELLLMKRGGRVIYGGKLGVQSQIMIDYFQRISGIPPIPSGYNPATWVLEVTTPAVEERIDADFADIYKKSDQYRGVEASIMQFQLPPAGSEPLKFDTMYSQNLLSQFFLCLWKQNLVYWRSPPYNAMRLFFTTVSALIFGTVFWDIGSKRASTQELFVVMGALYSASLFLGVNNASSVQPIVSIERTVFYREKAAGMYSPIAYAAAQGLVEIPYIAVQTIVFGVITYFMINFERTAGKFFLYLLFMFLTFTYFTFYGMMAVGLTSSQQLAAVISSAFYSLWNLLSGFLIPKSNIPGWWIWFYYICPVQWTLRGIITSQLGDVETKIVGPGFEGTVKEYLSASLGYENKINGFSSVGLSVIVLFGFVLLFFGSFAVSVKVLNFQKR
139.	GgABCG16	MT379800	MIVGPRKTLFMDEISTGLDSSTTYQIVKCIKNFVHQMDATVLMALLQPAPETFELFDDLVLLSEGHVVYEGPREDVLEFFQSIGFQLPPRKGIADFLQEVTSKKDQAKYWADHSKPYGFVSVREIAEAFRNSRFGSHMESLQAQPYDKSKCHTSALARNKFAVSKWEMTKACFQREILLIKRHSFLYIFRTFQVAFVGFVTCTIFLRTRLHPTDEAYGNLYLSALFFGLVHMMFNGFSELPLMITRLPVFYKQRDNLFYPAWAWSFTSWILRVPYSIIEAVIWTVVVYYTVGFAPAAGRFFRYMFLLFVVHQMAIGLFRMMAAIARDIVLANTFGAAALLIIFLLGGFIVPKAMIKPWWIWGYWLSPLTYGQRAITVNEFTATRWMKKSAIGYSTVGYNVLQLHSLPSDDYWYWAGVGILIVYAIFFNNMVTLALAYLNPLHKARTVIPLDDDSEKNSSRDGFAVSNLGHEMSTRTRSTRDNSTARGMILPFQPLTMTFHNVNYFVDMPKEIRKQGLPETRLQLLTDVSGVFSPGVLTALVGSSGAGKTTLMDVLAGRKTGGYIEGDIKISGYPKVQRTFARISGYVEQNDIHSPQVTIEESLWFSASHRLPKEISVDKRREFVEQVMKLVELDTLRDALVGMPGSSGLSTEQRKRLTIAVELVANPSIIFMDEPTSGLDARAAAIVMRTVRNTVDTGRTVVCTIHQPSIDIFEAFDELLLMKRGGRVIYGGKLGVQSQIMIDYFQRISGIPPIPSGYNPATWVLEVTTPAVEERIDADFADIYKKSDQYRGVEASIMQFQLPPAGSEPLKFDTMYSQNLLSQFFLCLWKQNLVYWRSPPYNAMRLFFTTVSALIFGTVFWDIGSKR
140.	GgABCG17	MT379801	MMLPEQETTIAPPAITNRPDQNSSEHAEGGSASASASATNKVKPSFDDNAIPSQQQHHPPTPSSSFPNILHQSLRPVTLKFEDLSYSVTLTNQKRNGCGGLGRETKVTRKVLSGVTGIASPGELTAMLGPSGSGKTTLLTALAGRLAGKLSGSITYNGNPDFRCMKRRIGFVSQDDVVYPHLTVLETLTYAALLRLPNTLTREEKVGHAERVISELGLTRCQNSPVGGRMGRFRGISGGERKRVSIGHEMLVNPSLLLLDEPTSGLDSTTAQMIVSVLRGLARSGRTVLTTIHQPSSRLYRMFDKVVVLSDGHPIYSGQSGRAMDYLESVGYVPAFNFVNPADFLLDLANGIVADVKHDDQIEHHEDQASVKQSLVSSYKKNLYPALKEEIQQSNSEPVVFTSRTPRRSDNQWNTSWWVQFKVLLKRGLQERRHESFSGLRIFQVLSVSVLSGLLWWHSDPSHIQDQVGLLFFFSIFWGFFPLFNAIFAFPLERPMITKERSSGMYHLSSYYVARMVGDLPMELVLPTIFVTITYWMGGLKPSLVTFVLTLLIMLFNVLVSQGIGLALGAILMDVKQATTLASVTMLVFLLAGGYYIQQMPSFIAWLKYISFSHYCYKLLVGVQYSVNEVYECGPGLHCRVRDFPAIKCLGLDNMWGDVALTMMLIGYRVVAYLALRMGQLR
141.	GgABCG18	MT379802	MMPPQQETTITPNIPATPNRPEKCSVHAEPAAAASSTNDVIKPTLDNDIIPSHHQQQQQAPSARLSILHQSLRPVTLKFEDVCYSITMGRPEHGCCVSVSPSKEPKPTRAVLNGVTGLVGPGEVMAMLGPSGSGKTTLLTALAGRLAGKLSGTITYNGNPFSGSTRRSTGFVSQDDVLYPHLTVHETLSYAALLRLPRSLTREQKMEHVDTVIAELGLSRCRNSPIGGGSALFRGISGGERKRVSIGQEMLVNPSLLLLDEPTSGLDATTAQRIVAVLRSLARGGRTVVTTIHQPSSRLYRMFDKVVVLLDGCPIYSGQAGRVMEYLEAVGFVPAFNFVNPADFLLDLANGIVADVKPNDMIDQHQDRQTSIKQSLISAYKKNLYPLLKEEIQRNNRDPTFFATGTRRSSENQWTTSWWEQFKVLLKRGLKERRHESYSGLRIFQVLSVSILSGLLWWHSDPSHIQDQVGLLFFFSIFWGFFPLFNAIFAFPLDRPMLMKERSSGMYHLSSYYVARMVGDLPMELVLPTIFVTITYWMGGLKPSLVTFVVTLLIMLFNVLVSQGIGLALGAILMDVKQATTLASV

			TMLVFLLAGGYYIQKIPSFIAWLKYISFSHYCYKLLVGVQYSVKDVYECGPGLYCRIRDFPAIKCLELDNMWGDVVVLTVMLIGYRIVAYLALMRMGLHH
142.	GgABCG19	MT379803	MEIEAAASGSNKGGAIESYSDEVVTYGGFDRGSFLAWEDLTVVLPNFGKGPTKRLLNGLNGYAEPGRIMAIMGPSGSGKSTLLDSLAGRLSKNVVMTGNVLLNGKKKSPGYGFVAYVTQEDVLLGTLTVKETISYSAHLRLPTSMPKEEVSSIIDGTIIEMGLQDCADRLIGNWHLRGISGGEKKRLSIALEILTRPRLLFLDEPTSGLDSASAFFVVQTLRNVARDGRTVISSIHQPSSEVFALFDDLFLLSGGETVYFGEAKMAIEVNFNYVNNIAFFLH
143.	GgABCG20	MT379804	MGQPDRKRGMVLPFEPYSITFDEIVYAVDMPQEMKGQGVTENKLVLLKGISGAFRPGVLTALMGVSGAGKTTLMDVLAGRKTGGYIDGTIKISGYPKRQETFARISGYCEQNDIHSPHVTVYESLIYSAWLRLSAEVDVNTRMVFTEEVMELVELNPLRNSLVGMPGESGLSTEQRKRLTIAVELVANPSIIFMDEPTSGLDARAAAIVMRTVRNTVDTGRTVVCTIHQPSIDVFEAFDELLLLKRGGQEVYVGPLGRHSSQMIKYFESIEGVAKIKDGYNPATWMLEATTPAQEHALGVDFYEIYKNSDLYR
144.	GgABCG21	MT379805	MGSTSEKRCFKLSSCNSNTVTQNMHAYGIMLIVALSTLLLIIYGCSDQVLATKERKLAKSREAAARSVRKTTNARQRWKAAKDSAKKASSGLQAQLSRTFSRTKEDMADPEEVKILSQSTTETDIELFSYSHRIITSSMAASSSAVPKQKGKEASDVMQMIHEIENDPDISDNFPVEKETIDKNVTAKRSNGGKQLHTHSQIFKYAYAQLEKEKAQQQENKNLTFSGVISMANKSEQRKRPLIEISFKDLTLTLKAQNKHILRCVTGKLKPGRIAAVMGPSGAGKTTFLSAIAGKAFGCKVTGSILINGKNESIHSYKKIIGFVPQDDIVHGNLTVEENLRFSAQCRLSADLSKPDQILVIERVIEFLGLQSVRNSLVGTVEKRGISGGQRKRVNVGLEMVMEPSLLILDEPTSGLDSTTALKIVQMLQDIAEAGKTVVTTIHQPSSRLFHKFDKLILLGKGSLLYFGKASEAMNYFQSIGCSPLISMNPAEFLLDLANGNINDVSLPSELEDRVQMGNAVAETHNGKPSPAVVHEYLVEAYETRVAETEKKKIMVSIPLDAASKVCCPKRQWGASWDEQYSILFWRGIKERRHDYFSWLRITQVLSTAIILGLLWWQSDAKNPKDLQDQAGLLFFIAVFWGFFPVFTAIFTFPQERAMLTKERAADMYRLSAYFLARTTSDLPLDLILPVLFLLVVYFMAGLRLSVAPFFFTILTVFLCIVAAQGLGLAIGATLMDLKRATTLASVTVMTFMLAGGFFVQKVPIFISWIRYMSFNYHTYKLLLKVQYEHITPSNSINGVKIDSGLTEVAALIAMVFGYRFLAYLSLRRMKLQSG
145.	GgABCG22	MT379806	MEIEASKGTPGNGVTLPGLSPLSETLWREKATNNTEFIGDVSARLTWRDLTVMVTLSNGETQNVLEGLTGYAEPGTFTALMGPSGSGKSTLLDALSSRLAANAFLSGTILLNGRKEKLSFGTAAYVTQDDNLIGTLTVRETIWYSARLRLPDKMSRSDKRALVESTIVAMGLQDCADTVIGNWHLRGISGGEKRRVSIALEILMRPRLLFLDEPTSGLDSASAFFVTQTLRALARDGRTVIASIHQPSSEVFELFDQLYLLSGGKTVYFGQASDAYEFFEQAGFPCPALRNPSDHFLRCINSDFDKVKATLKGSMKLRFEASEDPLDKITTAEAIRTLIDFYRTSQHSYAARQKVDEISKVKGTVLEAGGSEASFLMQSYTLTKRSFINMSRDFGYYWLRLVIYIVVTVCIGTIYLNVGTGYNSILARGSCASFVFGFVTFMSIGGFPSFVEDMKVFQRERLNGHYGVTAFVISNTLSATPFLILITFLSGTICYFMVRLHPGFWHYLFFVLCLYASVTVVESLMMAIASIVPNFLMGIIIGAGIQGIFMLVSGYFRLPHDIPKPVWRYPMSYISFHFWALQGQYQNDLKGLVFDNQTPDLPKIPGEYILENVFQIDVSRSKWIDLSVILSMIVIYRIIFFIVIKINEDVTPWVRGYLARRRMQQKSGAQNTTIAPDVLTQSPSLRTYISNQTRNVATSRTRN
146.	GgABCG23	MT379807	MQMKVQGCEVEAIGINYKIHTHKKEHPFKIFSKPPQLDDHGQEEAEEEEAKADEKCCSSGVRHVLKNVSCIAKPWEILAIVGPSGAGKSSLLEILAGKHSPQKGSVLVNHKPVDKAQFRKLSGYVTQRDTLFPLLTVEETMLFSAKLRLKLPQEQLCSRVKSLIQELGLDHVAGARIGDDRVRGISGGERRRVSIGVEVIHDPRVLILDEPTSGLDSTSALQIIDMLKVMAETRGRTIILSIHQPGFRIVKLFNSLLLLANGSVLHHGTADLLSVKLRLMGLELPLHVNVVEFAIDSIDTIQQQQRCQLFQQLETPRQLQGTMQQKKGGGGGDDEQQPVGEGKSGKFTLQQLFQQSKVIDEEIIKGGVDFSCDFANSRLKETMILTHRFSKNIFRTKELFACRTIQMLVSGLVLGSIFCNLKDDLNGAEERVGLFAFILTFLLSSSIEALPIFLQEREILMKETSCGSYRVSSYAIANGLVYLPFLLILAILFTVPLYWLVGLNKNFTAFLHFLLLIWLILYTANSVVVCFSALVPNFIVGNSVIAGVIGSFFLFSGYFISKHEIPKYWIFMHYISLFKYPFEGFLINEFSNSGKCLEYMFGACVMRGEDVLKEEGYGGESSRWKNVGVTVCFILFYRFISYVILRYRCSQRGFRSSVVMVQS
147.	GgABCG24	MT379808	MASFLPHVPSDRYEAKPSSSSIFPQSVLDIELATRSLEMEASDAPERMNRAGSNHQTKKMGICLTWKDLWVTASTGKNESISILQGLTGYAKPGQLLAIMGPSGCGKSTLLDALAGRLGSNTRQSGEILINGNKQTLAYGTSAYVTQDDTLLTTLTVREAVYYSAQLQLPDSMSKEEKKERAEFTIREMGLQDAVNTRIGGWGCKGISGGQKRRVSICIEILTHPRLLFLDEPTSGLDSAASYYVMRSIATLDQKDGIQRTIITSIHQPSSEIFQLFDNLCLLSSGRTVYFGPASAASEFFASNGFPCPALQNPSDHLLKTINKDFDQDIEVLTGRTLPTEEAIRILVRSYKSSERNQQVQNEVAILSTKNTSSMDKEEGHASFLNQCFVLTKRSSVNMFRDLGYYWLRLAIYIALAISLATLFCGLDKNYDSIQDRASFLMFVSTFLTFMTIGGFPSFVEDMKVFERERLNGHYGVTAFVIGNTFSAIPYLLLVSLIPGAIAYYPPGLQKGFDHFVYFICVLFTCLLLVESLMMIVASIVPNFLMGIITGAGIQGIMMLGGGYFRFSNDLPKPFWRYPWYYYVTFHRYAYQGLYKNEFEGLRFARNEGGSNDSLNYISGEEILRNKYHVDSLSKWVDLGILLGMVVLYRVLFLVIIKATEKLKPIVLSLMSASPRRATQIMENPNATPLREEVI
148.	GgABCG25	MT379809	MASFLPHVPSDRYEAKPSSSSIFPQSVLDIELATRSLEMEASDAPERMNRAGSNHQTKKMGICLTWKDLWVTASTGKNESISILQGLTGYAKPGQLLAIMGPSGCGKSTLLDALAGRLGSNTRQSGEILINGNKQTLAYGTSAYVTQDDTLLTTLTVREAVYYSAQLQLPDSMSKEEKKERAEFTIREMGLQDAVNTRIGGWGCKGISGGQKRRVSICIEILTHPRLLFLDEPTSGLDSAASYYVMRSIATLDQKDGIQRTIVMSIHQPSTEVFQLFHNLCLLSSGRTVYFGPASAASEFFASNGFPCPATQNPSDHLLKTINKDFDKDIVVGTRTIPTEEVIRILVSSYKSSERNQQVQNEVVVLSTMVSFMA
149.	GgABCG26	MT379810	MASFLPHVPSDRYEAKPSSSSIFPQSVLDIELATRSLEMEASDAPERMNRAGSNHQTKKMGICLTWKDLWVTASTGKNESISILQGLTGYAKPGQLLAIMGPSGCGKSTLLDALAGRLGSNTRQSGEILINGNKQTLAYGTSAYVTQDDTLLTTLTVREAVYYSAQLQLPDSMSKEEKKERAEFTIREMGLQDAVNTRIGGWGCKGISGGQKRRVSICIEILTHPRLLFLDEPTSGLDSAASYYVMRSIATLDQKDGIQRTIITSIHQPSSEIFQLFDNLCLLSSGRTVYFGPASAASEFFASNGFPCPALQNPSDHLLKTINKDFDQVTKNQ
150.	GgABCG27	MT379811	MASFLPHVPSDRYEAKPSSSSIFPQSVLDIELATRSLEMEASDAPERMNRAGSNHQTKKMGICLTWKDLWVTASTGKNESISILQGLTGYAKPGQLLAIMGPSGCGKSTLLDALAGRLGSNTRQSGEILINGNKQTLAYGTSAYVTQDDTLLTTLTVREAVYYSAQLQLPDSMSKEEKKERAEFTIREMGLQDAVNTRIGGWGCKGISGGQKRRVSICIEILTHPRLLFLDEPTSGLDSAASYYVMRSIATLDQKDGIQRTIVMSIHQPSTEVFQLFHNLCLLSSGRTVYFGPASAASEVRN
151.	GgABCG28	MT379812	MHTNKHTMGSPFKHISCTRKAPYRLETKNLSYKLCSQLDEYTSLCFGSNPSKGAKFILKDVNCEARPAELTAIAGPSGAGKTTLLEILAGRIPPCKVSGQVLVNHRPMEMNRFRRTSGYVTQEDALFPSLTVRETLMYSALLRLPGGQTVAAMRVAELMKELGLDHIADSRIGSGSESDHGMISGGERRRVSIGVDLVHDPAVILIDEPTSGLDSASAL

			NVVSLLRLMAFNQGKTVVLTIHQPGFRILELFDGLILLSDGFVMHNGSLNLLEARLNLAGHCIPEHVNVLEFALDVMESLVIHTSESGNSQLLLNDKEHQGNNKMRMQCSKVIKEKALLYTNSPIEEILILGQRFCSNIFRTKQLFVTRVIQALVAGFVLGSIFFNVGSKQSQVALQTRTGFFAFSLTFLLSSTTEGLPIFLEERRTFMRETSGGAYRVSSYVLANTLVFLPFLLMVGLLYTTPVYWLVGLRKDIDGFLYFSLVVWLVLLMSNSLVACFSSLVPNFILGSSVIAGLMGSFFLFSGYFISKEKIPSYWIFMHYLSLFKYPFECLMINEYGGKQGKRKCIEISNSGDCILHGVEFLRQQGLKVSQKWTNLAVMLGFIIGYRVLNFLILWSRCYKSRK
152.	GgABCG29	MT379813	MDEISTGLDSSTTYQIIRYLKHSTRAFDATTIISLLQPAPETYELFDDVILLTEGQIVYQGPREAALDFFKLMGFSCPERKNVADFLQEVTSKKDQEQYWSVLDRPYRYIPVGKFAQAFSLYREGRLLSEELNIPFDRRYNHPAALATCTYGAKRLELLKINFQWQRLLMKRNSFIYIFKFVQLLLVALITMSVFFRTTMHHNTIDDGGLYLGALYFSMVIILFNGFTEVSMLVAKLPVIYKHRDLHFYPSWAYTLPSWFLSIPTSLMEAGCWVVVSYYASGYDPALTRFFRQFLLFFFLHQMSIGLFRLIGSLGRHMIVANTFGSFAMLVVMALGGYIISKDRIPSWWIWGFWISPLMYAQNSASVNEFLGHSWDKKVGNQSTHPLGKAVLKERSLYPESYWYWIGLGALVGYTILFNILFTIFLAYLNPVGRRQAVVSKDELQEREKRRKGESVVIELREYLQHSASSGKHFKQRGMVLPFQPLSMAFSNINYYVDVPLELKQQGISEDRLQLLVNVTGAFRPGVLTALVGVSGAGKTTLMDVLAGRKTGGVIEGSIYISGYPKRQGTFARISGYCEQTDVHSPCLTVWESLLFSAWLRLSSDVDLETQKAFVEEIMELVELTPLSGALVGLPGVDGLSTEQRKRLTIAVELVANPSIVFMDEPTSGLDARAAAIVMRTVRNIVNTGRTIVCTIHQPSIDIFESFDELLFMKRGGELIYAGQLGPKSSELITYFEAIEGVPKIRSGYNPATWMLEVTSAAEENRLGVDFAEIYRKSSLYQYNHELVESLSKPSSNSKELHFPSKYCRSSFEQFLTCLWKQNLSYWRNPQYSAVRFFYTVIISLMLGTICWRFGAKRETQQDLFNAMGSMYSAILFIGITNATAVQPVVSVERFVSYRERAAGMYSALSFAFAQVVIEFPYVFAQAMIYSSIFYSMGSFVWTVDRFIWYLFFMYFTMLYFTFYGMMTTAVTPNHNVAAIIAAPFYMLWNLFSGFMIPHKRIPVWWRWYYWANPVAWSLYGLLTSQYGDDNKLVKLSDGNLITIRRVLRDVFGYRHDFLCVAATMVAGFCVFFAFIFAFAIKSFNFQRR
153.	GgABCG30	MT379814	MNMGYSHCVPLGTKLASYDETHDGSRQGESRLEIIGTKLGVGAKCGGEGVMIEVPVDEEEEEGVSLTWEDLCVTVSSSGKDGSKLEILEGLNGYAKPGELLAIMGPSGCGKTTLLDALAGRLSSNAKQSGNILINGHKQELAYGISAYLTQDEAILTTLTVEEAVYYSAQLQLPDSMSKSEKKERANLTIKEMGLQDAIHARIGGCGNSKGISGGQKRRVSICIEILTRPSLLFLDEPTSGLDSAASYYVMSRIARLNQKDGIQRTIIASIHQPSNEVFQLFHNLCLLSSGKIVYFGSAPAANKFFASNGFPCPPLQNPCDHFVKTINKDFEQENPEQGLATGLTNEEAIYVLVKSYKSSEISFQVQTEIAEIRKRDSSAMEKKSHAAFFNQCLILTRRSFVNMYRDVGYYWLRLFIYGALAISLGTIFYGIGSSKISAEARCSLLMFVATFLTFMTIGGFPSFVEEMKVFERERLNGHYGVTAYLIGQTLSSIPYLLLISLIPGALVYYLTALHPGGQHFLYFTCVLFVSVMLVESLMMIVASMVPNFLMGIITGAGILGVMMLAGGFYRLPSDLPKPFWRYPLYYISMHKHAYQGLFKNEFEGLTLTKNQVGETMNISGEEMLRTLWQVEMGHSKWVDLSILLGMVILYRISFLVIIKSMEKVKPIVAAFK
154.	GgABCG31	MT379815	MNMGYSHCVPLGTKLASYDETHDGSRQGESRLEIIGTKLGVGAKCGGEGVMIEVPVDEEEEEGVSLTWEDLCVTVSSSGKDGSKLEILEGLNGYAKPGELLAIMGPSGCGKTTLLDALAGRLSSNAKQSGNILINGHKQELAYGISAYLTQDEAILTTLTVEEAVYYSAQLQLPDSMSKSEKKERANLTIKEMGLQDAIHARIGGCGNSKGISGGQKRRVSICIEILTRPSLLFLDEPTSGLDSAASYYVMSRIARLNQKDGIQRTIIASIHQPSNEVFQLFHNLCLLSSGKIVYFGSAPAANKVGISKHYFMYE
155.	GgABCG32	MT379816	MGGHEGEKGNNITVTWENLEATVTDGKNGKLILNGLTGYAQPGKLLAVMGPSGCGKSTLLDALAGRLRSNVKQSGKILINGQKHALAYGTSGYVTQDDAMLSTLTAGETLYYSAQLQFPDSMSTAEKKRRADITLREMGLQDAINTRVGGWGSKGLSGGQKRRLSICIEILTHPRLLFLDEPTSGLDSAASYYVMSRIASLSQRDDIQRTIVASIHQPSSEVFELFHDLCLLSSGETVYFGPASDANQFFASNGFPCPTLHNPSDHYLRIINKDFEQDAEEGFGKGVTTEEATGILVNSYKTSEIRNQVQIEVTKISEGESGAVGKKRTHAAFLTQCLVLIRRSSVQMYRDISNYWLRLVVFIAIAISLGSIFYHIGPSTGSIQGRGSLLTFFISVLTFMTLVGGFSPLIEEMKVLERERLNGHYGVTAFLIGNIFSAVPYMLMISLIPGAIVYYLSGLHNGLTHFLYFASVLFAIVMWVESLMMVVGSIFPNFVMGVIIAGGVEGLMILTGGFYRLPNELPKPLWKYPFYYVSFLTYAFQGSFKNEFEGLTFVLYQDGGARAVSGRDILAETWHVQMGHSKWVDLAIMFGMILFYRVLFLAINKGKEKYSRRIVPAINGPQAKIFSRTTNMDEL
156.	GgABCG33	MT379817	MGPSGSGKTTLLNVLAGQLAASPRLHLSGLLEFNGKPGSNNTYKFAYVRQEDLFFSQLTVRETLSLATELQLPHISSEEERDEFVNNLLFKIGLVSCADTNVGDAKVRGISGGEKKRLSLACELLASPSVIFADEPTTGKHALVKFGLLWIGTLLTI
157.	GgABCG34	MT379818	MAAACLKPPSNEDDSVILFSTSNSPQDSTSPSSSSFYHYSPSPALHQFRVAYKLSVKNLTYTLHPHKTTPFFSFSHLTKKPQPVINILKHVSFVTTSSEIVAVVGPSGTGKSTLLRVIAGRVKDKDFDPKSVSINDQPMTSPAQLRKICGFVAQEDNLLPLLTVKETLLFSAKFRLKEMTPKDRELRVESLMQELGLFHVADSFVGDEENRGVSGGERKRVSIGVDMIHNPPILVLDEPTSGLDSTSALHVIELLSSMAKAKQRTVVLSIHQPSYRILQYISRFLILSHGSVVHNGSLESLEETISKLGFQIPIQLNALEFSMEIVHRLEDSSSKYEEKEPFLPSSIMWPEEEEYRRESFGYTLCYVNLMEIIFLCSRFWKIIYRTKQLFLARTMQALVGGFGLGSVYIKVRKDEGGVAERLGLFAFSLSFLLSSTVEALPIYLQERSVVMKEASRGAYRISSYMIANTFVFLPFLFVVSLLFAAPVYWLVGLNPSLTAFTFFTLVVWLIVLMASSLVLFLSAVSPDFISGNSLICTVLGAFFLFSGYFIPKESIPKYWLFMYYVSLYRYPLDALLTNEYWNVRNECFSQQTQGSSQCLMTGFDVLKSRGLEKDDRWVNVGIMLGFFVFYRVFCWVVLARKASKTII
158.	GgABCG35	MT379819	MSGNVLLNGKKRRLDYGVVAYVTQEDILLGTLTVRETISYSANLRLPATMTKEEVNDIVEGTIMEMGLQDCADRLIGNWHLRGISGGEKKRLSIALEILTRPCLLFLDEPTSGLDSASAYFVAQTLRNIAHDGKTVISSIHQPSSEVFALFDDLFLLSGGQTIYFGAAEKAVEFFSKAGFPCPSRRNPSDHFLRCINSDFDTVTTTMVASGRNIHEQKSLTPSLVNLSTAAIKAILIEKYRWSEYATSARARIKEISNFEGHDSESKSSKSQAKWWKQLSTLTRRSFVNMSRDVGYYWIRLTIYVALSLCVGTIFFEVGSSYRAIFARGACGAFISGFMTFMSIGGFPSFIEEMKVFYKERLNGYYGISVYILSNFLSSFPFVAMMSIATGTITYYMVKFRPEFSHLLYICLDLLGCIAVVESSMMIIAALVPNFLMGLIIGAGYIGVMMMTAGYFRQIPDLPKFFWRYPMSYINYGAWGLQGAFKNDMIGMEFDPLVPGGPKLKGEIILTSMLGMQVDHSKWWDLAVVITILILLRFSFFFILKFKERAAPFLHSIYAKQTLERIKKRPSFRKTPSPFPSKRHQSLHPLSFQEGLNSPIH
159.	GgABCG36	MT379820	MKVLQINSQVLTIFDGDVIKSTEISGNVLEEGITVTWENLWVTVPNGKERKPILEGLTGYAQPGRLLAIMGPSGCGKSTLLEGLAGRLSSNMKHTGNILINGHKEELAYGISGCVTQDDAMLSTLTAGETLYYSAQLQFPDSMSTAEKKRRADITLREMGLQDAINTRVGGWGSKGLSGGQKRRLSICIEILTHPRLLFLDEPTSGLDSAASYYVMSRIASLSQRDDIQRTIVASIHQPSSEVFELFHDLCLLSSGETVYFGPASDANQFFASNGFPCPTLHNPSDHYLRIINKDFEQDAEEGFGKGVTTEEATGILVNSYKTSEIRNQVQIEVTKISEGESGAVGKKRTHAAFLTQCLVLIRRSSVQMY

			RDISNYWLRLVVFIAIAISLGSIFYHIGPSTGSIQGRGSLLTFFISVLTFMTLVGGFSPLIEEMKVLERERLNGHYGVTAFLIGNIFSAVPYMLMISLIPGAIVYYLSGLHNGLTHFLYFASVLFAIVMWVESLMMVVGSIFPNFVMGVIIAGGVEGLMILTGGFYRLPNELPKPLWKYPFYYVSFLTYAFQGSFKNEFEGLTFVLYQDGGARAVSGRDILAETWHVQMGHSKWVDLAIMFGMILFYRVLFLAINKGKEKYSRRIVPAINGPQAKIFSRTTNMDEL
160.	GgABCG37	MT379821	MMMEPASEQNQPPPKSYTLTATSISYTKQTSSSSTIALFKHCTPTPPTHILKDVSLTAYPSEILAIVGPSGAGKSTLLDILSARRLPSSGTLSLNSSPLNPSTFRKLSSYVPQHDACLPLLTVSETFAFSAQLLRPKTKTSSGDVATIVSSLLNELRLTHLANTRLAHGLSGGERRRVSIGLSLLHDPAVLLLDEPTSGLDSTSAFKVMQILKSTCVSRHRTIVLSIHQPSFKILSCIDRILLLSKGTVVHHGSLASLQAFLHSKGFTVPHQLNALEYAMEILNQLNELKPLTITPPSPPESPESSSSKPDNITTTAAREMIRYKSSRIHEICALYSRFWKIIYRTRQLLLTNTAEALLVGLVLGTIYINIGFDKEGIEKRFGLFAFTLTFLLSSTTETLPIFINERPILLRETSSGVYRLSSYLIANTLVFLPYLLVVAVIYSIPVYFLVGLCASWLSFAYFVLVIWVIVLMANSFVLFLSSLAPNYIAGTSLLTVLLAAFFLFSGYFISKDSLPKYWLFMHFFSMYKYALDALLINEYSCLVSRCMIWYRENQECMVTGGDVLQKRGLHLGERWTNVYFLIGFFVFYRVLCYLVLIRRVSRSKT
161.	GgABCG38	MT379822	MTGNVLLNGKKKSPGYGFVAYVTQEDVLLGTLTVKETISYSAHLRLPTSMPKEEVSSIIDGTIIEMGLQDCADRLIGNWHLRGISGGEKKRLSIALEILTRPRLLFLDEPTSGLDSASAFFVVQTLRNVARDGRTVISSIHQPSSEVFALFDDLFLLSGGETVYFGEAKMAIEFFAEAGFPCPRKRNPSDHFLRCINSDFDIVTATLKGSQRIRDVPNSSDPFMNLATAEIRAMLVEKYRRSTYAKRAKDRIQELSTNEGLETGAQHTSQASWWKQLTTLTRRSFVNMCRDVGYYWLRIIIYTIVSVCVGTIYFDIGYSYTSILARCACGAFISGFMTFMSIGGFPSFIEEMKVFYRERLNGYYGVAAYILANFLSSFPFLVAVSLVTGTITYNMVKFRPGFSHYVFFTLNIYSSISVIESLMMVVASLVPNFLMGIITGAGIIGIMMMTSGFFRLLSDLPKPVWRYPISYISYGAWAIQGAYKNDLLGLEFDPLIPGDPKLKGEYVITHMLGIELNHSKWWDLAALFTILIFYRVLFFTILKFKERASPLFKTLYAKRTIQQLEKRPSFRKIPSFPSKRHQPLHSLSSQEGLNSPLH
162.	GgABCG39	MT379823	MEEIQSQSDNYRSSSSSASSPASRVPSSNFFYLRKPGSIRQPISFEDSPEWGDDTDVDVRVESVDEGGDSINVATTPASPSLSKLNSGSLPSPHLPEGAVIPRKIAGASVAWKDLTVTIKGKRKYSDKVIKSSTGYALPGTMTVIMGPAKSGKSTLLRAIAGRLLPSARMYGEVFVNGTKSQMPYGSYGYVERETTLIGSLTVREFLYYSALLQLPGFFCQKKSVVEDAIHAMSLGEHANKLIGGHCYMKGLPSGERRLVSIARELVMRPRILFIDEPLYHLDSVSALLMMVTLKRFASTGCTLIVTIYQSSTEVFGLFDRICLLSNGNTLFFGETLSCLQHFSNAGFPCPIMQSPSDHFLRAINTDFDRIIAMCKNWQDDNGDFSSVNMDTAVAIRTLEATYKSSADAAAVETMILKLTEKVYYNC
163.	GgABCG40	MT379824	MEEIQSQSDNYRSSSSSASSPASRVPSSNFFYLRKPGSIRQPISFEDSPEWGDDTDVDVRVESVDEGGDSINVATTPASPSLSKLNSGSLPSPHLPEGAVIPRKIAGASVAWKDLTVTIKGKRKYSDKVIKSSTGYALPGTMTVIMGPAKSGKSTLLRAIAGRLLPSARMYGEVFVNGTKSQMPYGSYGYVERETTLIGSLTVREFLYYSALLQLPGFFCQKKSVVEDAIHAMSLGEHANKLIGGHCYMKGLPSGERRLVSIARELVMRPRILFIDEPLYHLDSVSALLMMVTLKRFASTGCTLIVTIYQSSTEVFGLFDRICLLSNGNTLFFGETLSCLQVTYLLYLLAPAHEI
164.	GgABCG41	MT379825	MLSTLTAGETLYYSAQLQFPDSMSTAEKKRRADITLREMGLQDAINTRVGGWGSKGLSGGQKRRLSICIEILTHPRLLFLDEPTSGLDSAASYYVMSRIASLSQRDDIQRTIVASIHQPSSEVFELFHDLCLLSSGETVYFGPASDANQFFASNGFPCPTLHNPSDHYLRIINKDFEQDAEEGFGKGVTTEEATGILVNSYKTSEIRNQVQIEVTKISEGESGAVGKKRTHAAFLTQCLVLIRRSSVQMYRDISNYWLRLVVFIAIAISLGSIFYHIGPSTGSIQGRGSLLTFFISVLTFMTLVGGFSPLIEEMKVLERERLNGHYGVTAFLIGNIFSAVPYMLMISLIPGAIVYYLSGLHNGLTHFLYFASVLFAIVMWVESLMMVVGSIFPNFVMGVIIAGGVEGLMILTGGFYRLPNELPKPLWKYPFYYVSFLTYAFQGSFKNEFEGLTFVLYQDGGARAVSGRDILAETWHVQMGHSKWVDLAIMFGMILFYRVLFLAINKGKEKYSRRIVPAINGPQAKIFSRTTNMDEL
165.	GgABCG42	MT379826	MEEIQSQSDNYRSSSSSASSPASRVPSSNFFYLRKPGSIRQPISFEDSPEWGDDTDVDVRVESVDEGGDSINVATTPASPSLSKLNSGSLPSPHLPEGAVIPRKIAGASVAWKDLTVTIKGKRKYSDKVIKSSTGYALPGTMTVIMGPAKSGKSTLLRAIAGRLLPSARMYGEVFVNGTKSQMPYGSYGYVERETTLIGSLTVREFLYYSALLQLPGFFCQKKSVVEDAIHAMSLGEHANKLIGGHCYMKGLPSGERRLVSIARELVMRPRILFIDEPLYHLDRCFFYCNNSLSVA
166.	GgABCG43	MT379827	MQVALSTLLLIIYGCSDQVLATKERKLAKSREAAARSVRKTTNARQRWKAAKDSAKKASSGLQAQLSRTFSRTKEDMADPEEVKILSQSTTETDIELFSYSHRIITSSMAASSSAVPKQKGKEASDVMQMIHEIENDPDISDNFPVEKETIDKNVTAKRSNGGKQLHTHSQIFKYAYAQLEKEKAQQQENKNLTFSGVISMANKSEQRKRPLIEISFKDLTLTLKAQNKHILRCVTGKLKPGRIAAVMGPSGAGKTTFLSAIAGKAFGCKVTGSILINGKNESIHSYKKIIGFVPQDDIVHGNLTVEENLRFSAQCR
167.	GgABCG44	MT379828	MGSTSEKRCFKLSSCNSNTVTQNMHAYGIMLIVALSTLLLIIYGCSDQVLATKERKLAKSREAAARSVRKTTNARQRWKAAKDSAKKASSGLQAQLSRTFSRTKEDMADPEEVKILSQSTTETDIELFSYSHRIITSSMAASSSAVPKQKGKEASDVMQMIHEIENDPDISDNFPVEKETIDKNVTAKRSNGGKQLHTHSQIFKYAYAQLEKEKAQQQENKNLTFSGVISMANKSEQRKRPLIEISFKDLTLTLKAQNKHILRCVTGKLKPGRIAAVMGPSGAGKTTFLSAIAGKAFGCKVTGSILINGKNESIHSYKKIIGFVPQDDIVHGNLTVEENLRFSAQCR
168.	GgABCG45	MT379829	MTVRETLDFSGRCLGVGTRHDLLVELTRREKEAGITPDPEIDVFMKATAMEGQETSLITDYVLKILGLEICADTLVGDEMRRGISGGEKKRLTTGEMLVGPAKVFLMDEISTGLDSSTTFQIVRFLRQLVHIMDVTMIISLLQPAPETFDLFDDIILLSEGQIVYQGPRESVLNFFESVGFKCPERKGVADFLQEVTSRKDQEQYWFPRGNKPYHYISVPEFVAHFNNYFIGQKLSEELEVPYDRAKTHPAALVKDKYGISKPELFKACFAREWLLLKRSAFIYIFKTTQIMIMSLIAMTVFFRTEMKYSQLEDGRKYYGALFFSLINIMFNGMAELALTIFRLPVFFKQRDLLFYPAWAFALPIWILRIPLSFLESGLWVVLTYYTIGFAPAASRFFRQLLAFFCVHQMGLSLFRFIAALGRTQVVANTLGTFILLLVFLLGGFIIARDDIEPWMIWGYYASPMMYGQNAIAINEFLDKRWSNPNLDPRIPEPTVGKALLKARSMFTEDYWYWICVGALLGFSLLFNICFIVALTFLNPFGNSKSIILEEENEKKGTTKESSVSTAKSFENAEIEMAERKTPESSISKADAATTKRGMVLPFRPLSLAFDQVNYYIDMPTEMKKQGVEESRLQLLRDVSGAFRPGVLTALVGVTGAGKTTLMDVLAGRKTGGYIEGCISISGYPKNQETFARISGYCEQNDIHSPNLTVYESVVFSAWLRLSKEIKSEIQKMFVEEVMKLVELHPVRNFIVGLPGIDGLSTEQRKRLTIAVELVANPSIIFMDEPTSGLDARAAAIVMRTVRNTADTGRTVVCTIHQPSIDIFEAFDEVIRDDILVKRCCYFLIWLSIQVELD
169.	GgABCG46	MT379830	MTTKWGGLLKKNTKAEEKIILNGVTGMVQPGEILAILGPSGSGKTTLLTAFGGRLGGKLYGSITYNGKALSNAMKRNIGFVTQDDVLHPHLTVTETLVFSALLRLPSCFTKEEKIEHAKDVIAQIGLTKCKDSIIGGPMLRGVSGGERKRISIGQEMLINPSLLFLDEPTSGLDSTTAQRIVSTLWDLARGGRTIVMTIHQPSSRLYYLFHKVLLLAEGNPL

			YFGKGSEAIEYFSNIGYSPAMAMNPSDFLLDLANGIYTDDLNQDHIIDKQQLTSAFKSNFDAQLKPSAQEEISD<br>PDQSQGGFQDKGIGKWPTSWSQQFFVLLRRDIKERKYESFSGLRICQVLVVALISGLLWYKSDISHLQDQIGLL<br>FFITGFWGFFPLFQAIFTFPQELMMLEKERSSGMYRLSSYFMSRMVADLPMELVLPTIFLLITYWMAALKANM<br>VNFLYTLFSLLLNVLVSQGLGLAIGAVVLDQKSAATLASVIMLCFLLAGGFYVQNVPKFIAWVKYISINYYAY<br>QLLVGSQYDTSDTYPCSNGHQCSIAEFPTIKKMGLHLQGQVMAALALFIMLIGYRLMAYFALMRIGVTKKSV
170.	GgABCG47	MT379831	MEPQTDIFTWKDLVYDIEIKGEPRRLLDNVTGWVKPGTLTALMGVSGAGKTTLLDVLAQRTTMGVITGDLFV<br>NGQPLDASFQRKTGYVQQQDLHLDTSTVRESLRFSAMLRQPKSVSTEEKHQWVEEVIDMLNMRDFANAVV<br>GVPGEGLNVEQRKLLTIGVELAAKPKLLLFLDEPTSGLDSQSSWAIVAFLRKLADAGQAILCTVHQPSAVLFQ<br>QFDRLLFLARGGKTVYFGDIGDNSRKLLTYFENNGARACGDEENPAEWMLEIVNNGTNSSGQDWHSVWKA<br>SQERADVYAEVDRIHMEKPNSSSNQDTADSHSEFAMPFADQLREVTVRVFQQYWRMPSYILSKLMLGTIAGL<br>FVGFSFWKADGTLAGMQNIMFAVFMIITIFSTIVQQIQPHFVTQRSLYEVRERPSKAYSWKAFMIANVIVEIPY<br>QILTGILIFATFYYPIVGIQSSARQGLVLLFMIQLLLYASSFAQMTIAALPDALTASGIVTLLVLLSLTFCGVMQS<br>PTALPGFWIFMYRVSPFTYWVAGIVSTQLAGRAVTCSAAETSIFNPPDNQTCGEYLADYLKMAPGQLQNPDA<br>RENCSYCSLTNADQFMAGSNIYNSERWRNFGIVWVYIVFNIFVAVVSYYLFRVKKWNIGKKKKD
171.	GgABCG48	MT379832	MVGHTGKMVLPFEPLTIAFKDVQYFVDTPPEMKRHGSNEKKLQLLRDITGAFRPGILTALMGVSGAGKTTLM<br>DVLSGRKTGGVIEGDIRIGGYPKVQKTFARVSGYCEQNDIHSPYITVEESVRYSAWLRLPREIDSATKGKFVEE<br>VLETIELDDIKDSLVGIPGQSGLSTEQRKRLTIAVELVSNPSIIFMDEPTSGLDARAAAVVMRAVKNVVATGRT<br>IVCTIHQPSIDIFETFDELILMKSGGQIIYNGMLGHHSSRLIEYFQSIPGVPKIKDNYNPATWMLEATSASVEHEL<br>KIDFAKIYKESHLNRDTLELVRQLSEPLPGSKDLHFSTRFPQNLVGQFMACLWKQHLSYWRSPEYNLTRFVF<br>MVVAAIIFGAVFWQKGKEM
172.	GgABCG49	MT379833	MGQPDRKRGMVLPFEPYSITFDEIVYAVDMPQEMKGQGVTENKLVLLKGISGAFRPGVLTALMGVSGAGKT<br>TLMDVLAGRKTGGYIDGTIKISGYPKRQETFARISGYCEQNDIHSPHVTVYESLIYSAWLRLSAEVDVNTRMV<br>FTEEVMELVELNPLRNSLVGMPGESGLSTEQRKRLTIAVELVANPSIIFMDEPTSGLDARAAAIVMRTVRNTV<br>DTGRTVVCTIHQPSIDVFEAFDELLLLKRGGQEVYVGPLGRHSSQMIKYFESIEGVAKIKDGYNPATWMLEAT<br>TPAQEHALGVDFYEIYKNSDLYRRNKQLIEELGKPAPGSKDLNFPTQYSQPLWVQCKACLWKQRWSYWRNP<br>PYTAVRFFFTMVVSLMLGTMFWDLGQKYSKGQDLSNAMGSMYTALLFVGIMNSASVQPVVAVERTVFYRE<br>RAAGMYSALPYALAQVLIELPYIFAQSGTYCLLVYAMIGFDWTVKKFFWYLFFSYFTLSYFTFYGMMAVAVT<br>PNHHIASIVGAAFYGLWNLFSGFIIARPGMPIWWRWYYWANPLAWTLYGMIGSQYGDVTTIMKAENMSVQE<br>YVRSSLGIKHDFIGVCAVVVFGCAILFAFIFAVSIKLFNFQKR
173.	GgABCG50	MT379834	MKKQGVEESRLQLLRDVSGAFRPGVLTALVGVTGAGKTTLMDVLAGRKTGGYIEGCISISGYPKNQETFARIS<br>GYCEQNDIHSPFVTVYESLLYSAWLRLSAEINAETRKVGI
174.	GgABCI1	MT379835	MMAVVSLPLQFRYSPTTLRSPPKSTLSTHRRVSHRTCVASSERPLLQVNDLRAKIVESNVEILHGVNLTVNRG<br>EVHAIMGKNGSGKSTFAKVLVGHPDYEVTGGTVVFKGENLLEMEPEERSLAGLFMSFQSPVEIPGVSNDEFL<br>VMAYNARRKKLGLPELGPLECFSYLMEKLQLVNMKPDFLNRNVNEGFSGGERKRNEILQLAVLGADMAILD<br>EIDSGLDVDALRDVASAVNKILTPENSLLMITHYRRILDLLNPTHVHVMDKGKITRTGDISMVEAIEAKGYET<br>ASALN
175.	GgABCI2	MT379836	MMAVVSLPLQFRYSPTTLRSPPKSTLSTHRRVSHRTCVASSERPLLQVNDLRAKIVESNVEILHGVNLTVNRG<br>EVHAIMGKNGSGKSTFAKVLVGHPDYEVTGGTVVFKGENLLEMEPEERSLAGLFMSFQSPVEIPGVSNDEFL<br>VMAYNARRKKLGLPELGPLEVTLSFLFTPAFVKLFNNQSSFNNNVSSK
176.	GgABCI3	MT379837	MLCYAMNLPSLARFPERLVAPLYSTVPTTATRSKSVDNFAIEGRNLNFSFTTRQTKGVPILRDCSLRIPSGQFW<br>MLLGPNGCGKSTLLKILAGLLTPTSGTVYVNEPKSFVFQNPDHQVVMPTVDSDVAFGLGKINLTNDEVRSRV<br>SRALHAVGLSDYMKRSVQTLSGGQKQRVAIAGALAEACKVLLLDELTTFLDETDQK
177.	GgABCI4	MT379838	MLCYAMNLPSLARFPERLVAPLYSTVPTTATRSKSVDNFAIEGRNLNFSFTTRQTKGVPILRDCSLRIPSGQFW<br>MLLGPNGCGKSTLLKILAGLLTPTSGTVYVNEPKSFVFQNPDHQVVMPTVDSDVAFGLGKINLTNDEVRSRV<br>SRALHAVGLSDYMKRSVQTLSGGQKQRVAIAGALAEACKVLLLDELTTFLDETDQMGVIKAVRNCLDTSAE<br>VTALWVTHRLEELEYADGAIYMEDGKVVMHGDAASIRSFIEARQSDYINQINS
178.	GgABCI5	MT379839	MSSPLLEECREHLLEVEEDGYANGSGATKPKFQIRNLTKVSDDGVPILKGITVDIPKGVIVGVIGPSGSGKSTLL<br>RALNRLWEPPSSSVFLDGHDISHLDVLSLRRKVGMLFQLPALFQGTVADNVRYGPQLRGKKLTDEEVRGLLV<br>MADLDPSFLDKSGADLSVGQAQRVALARTLANSPEVISN
179.	GgABCI6	MT379840	MSSPLLEECREHLLEVEEDGYANGSGATKPKFQIRNLTKVSDDGVPILKGITVDIPKGVIVGVIGPSGSGKSTLL<br>RALNRLWEPPSSSVFLDGHDISHLDVLSLRRKVGMLFQLPALFQGTVADNVRYGPQLRGKKLTDEEVRGLLV<br>MADLDPSFLDKSGADLSVGQAQRVALARTLANSPEVLLLDEPTSALDPISTENIEEALMKLNKSRGMTLVMV<br>SHSIKQIQRMADIVCLLVDGQIVEVLKPDKLSQASHPMAQRFLQLSS
180.	GgABCI7	MT379841	MPIRKTPFPRLLLNNVSCMRNAQQVLRHVNISLRYGGALVLTGANGSGKTTFLRMLAGFSRPSAGEILWNGH<br>NTQDSGIYQQYKLQLNWLSLKDAINEKLTVLENVQWFEILEGKQGKSLPALELMGLGRLAREKPRMLSMGQ<br>RKRLQLARLLAIDRPIWLLDEPSVALDDEGVNLLEFIIAEHRKHGGIVIVATHIPIKIEDSMVLRLPPRFPRRMTF<br>VDMLDRADIS
181.	GgABCI8	MT379842	MVSLSTNPFFPLTAQKASPSSTTIRFPPPNKVIPLYHHTKDHRRKVVCACIAPPPHFNTHDSSAIQFNGSSKSEQ<br>LSTARDHEDDSDVLIECRDVYKSFGEKKILNGVSFKIRHGEAVGIIGPSGTGKSTVLKIIAGLLAPDNGEVYIRG<br>RKRVGLVSDDEISGLRIGLVFQSAALFDSLTVRENVGFLLYEHSSMPEGRISELVKESLAAVGLKGVENRLPSE<br>LSGGMKKRVALARSIIYDTSRDSIEPEVLLYDEPTAGLDPIASTVVEDLIRSVHIKGRDAVGKPGNIASYVVVT<br>HQHSTIKRAIDRLG

Supplementary Table S2. Primer sequences (forward/reverse) of the Real-time expression studies for the selected *GgABCBs*, *GgPIN1*, *GgPIN3/4/7*, *GgTWD1* and *Actin* genes investigated in the present study.

SNo.	Gene Name	Name	Sequence (5' to 3')
1	*Actin*	Forward	AGCCTGGATGGCAACATACATAGC
		Reverse	ATGACATGGAGAAGATCTGGCATCA
2	*GgABCB6*	Forward	AGGTTTAGTCGTAGCATTCATCAAGGGT
		Reverse	TTCAGAATAAGCTGCTTGTCCACGGGAT
3	*GgABCB14*	Forward	ATGTGGATCTCTTAGTGCCTTCTTTGCT
		Reverse	GATGAAAGCCCAATCAACAAGTAGCAGT
4	*GgABCB15*	Forward	AGACGGTTGCGCAAATCCGAGTAGTGCT
		Reverse	CATTCCCTTTGCTAACCCAGTTTTGTA
5	*GgABCB25*	Forward	AGTTTTCTGGTATGCTGGAGTTTTTATT
		Reverse	CTGAAAGCACCCAAGTTTGAGAATGAC
6	*GgABCB26*	Forward	GCTTCAACAGGAGTTTGGGTCAGTCATT
		Reverse	GGTAAGCACTTGCAATCTGATGGATCTT
7	*GgABCB31*	Forward	TAGCCAACGCCCATAGGTTCATTAGTGG
		Reverse	ATATCTTCGGACTTTTGATTATAGCAC
8	*GgABCB40*	Forward	TTGTAGGCCTGAGGTAACTGTGTTGAG
		Reverse	CAAATCCACACCACTCATCATAACCTTT
9	*GgABCB42*	Forward	TCTAAGACAATCCGAGTTTTCATGGTCC
		Reverse	TGACCGTAGAACTGAAATTGAACCGGAT
10	*GgABCB44*	Forward	TTGTTGGTGATGCGCTGGGACTATTAGT
		Reverse	GGAATCAAGACAAGAATAATCAACGA
11	*GgPIN1*	Forward	GGTCCAAATTGTGGTCCTCC
		Reverse	CATGACATCAGAGTCCACGTG
12	*GgPIN3/4/7*	Forward	ATGATGACTCCACGGCCTTC
		Reverse	GGAATACAAATCGGCGGCAC
13	*GgTWD1*	Forward	AAAGAAGGAAAAGCTCGCAGTG
		Reverse	CATACTGTTGCATAGCCTCCTCT

Supplementary Table S3. Topological inventory of ABC transporters identified from *Glycyrrhiza glabra* with their AtABC homologs identified in *Arabidopsis thaliana*. * indicates the trimmed 5' or 3' domain. Genes were identified by comparing with the domain architecture of *Arabidopsis thaliana* homolgous gene sequences.

Subfamily	Gene	Topology	Homologous with Arabidopsis	Sequence Comparison	Functions as reported	Reference
A (11)	GgABCA1	*TMD-NBD (Half)	AtABCA1(Full)	Incomplete	Seed and Pollen germination	24, 25
	GgABCA2	*TMD-NBD (Half)	AtABCA1(Full)	Incomplete	Seed and Pollen germination	24, 25
	GgABCA3	*TMD-NBD (Half)	AtABCA1(Full)	Incomplete	Seed and Pollen germination	24, 25
	GgABCA4	*TMD-NBD (Half)	AtABCA1(Full)	Incomplete	Seed and Pollen germination	24, 25
	GgABCA5	TMD-NBD (Half)	AtABCA7(Half)	Complete	Uncharacterized	
	GgABCA6	TMD-NBD (Half)	AtABCA2(Half)	Complete	Uncharacterized	
	GgABCA7	NBD(Quarter)	AtABCA1(Full)	Incomplete	Seed and Pollen germination	24, 25
	GgABCA8	NBD(Quarter)	AtABCA1(Full)	Incomplete	Seed and Pollen germination	24, 25
	GgABCA9	NBD(Quarter)	AtABCA2(Half)	Incomplete	Uncharacterized	
	GgABCA10	NBD(Quarter)	AtABCA2(Half)	Incomplete	Uncharacterized	
	GgABCA11	NBD(Quarter)	AtABCA7(Half)	Incomplete	Uncharacterized	
	GgABCB1	TMD-NBD(Half)	AtABCB29(Half)	Complete	Uncharacterized	
	GgABCB2	TMD-NBD(Half)	AtABCB12(Full)	Incomplete	Uncharacterized	
	GgABCB3	NBD(Quarter)	AtABCB26(Half)	Incomplete	Uncharacterized	
	GgABCB4	TMD-NBD(Half)	AtABCB14(Full)	Incomplete	Auxin and Malate transport	34, 35, 83
	GgABCB5	TMD-NBD(Half)	AtABCB27(Half)	Complete	Al and Fe sequestration	33
	GgABCB6	TMD-NBD-TMD*(Half)	AtABCB11(Full)	Incomplete	Uncharacterized	
	GgABCB7	*TMD-NBD(Half)	AtABCB13(Full)	Incomplete	Uncharacterized	
	GgABCB8	TMD-NBD(Half)	AtABCB13(Full)	Incomplete	Uncharacterized	
	GgABCB9	TMD-NBD(Half)	AtABCB10(Full)	Incomplete	Uncharacterized	
	GgABCB10	*TMD-NBD(Half)	AtABCB10(Full)	Incomplete	Uncharacterized	

B (45)	GgABCB11	TMD-NBD(Half)	AtABCB2(Full)	Incomplete	Uncharacterized	
	GgABCB12	TMD-NBD(Half)	AtABCB10(Full)	Incomplete	Uncharacterized	
	GgABCB13	*NBD-TMD-NBD(Half)	AtABCB1(Full)	Incomplete	Auxin transport	34, 35
	GgABCB14	TMD-NBD(Half)	AtABCB1(Full)	Incomplete	Auxin transport	34, 35
	GgABCB15	TMD-NBD- TMD-NBD(Full)	AtABCB1(Full)	Complete	Auxin transport	34, 35
	GgABCB16	NBD(Quarter)	AtABCB28(Half)	Incomplete	Uncharacterized	
	GgABCB17	NBD(Quarter)	AtABCB26(Half)	Incomplete	Uncharacterized	
	GgABCB18	NBD(Quarter)	AtABCB4(Full)	Incomplete	Auxin transport	73
	GgABCB19	TMD-NBD(Half)	AtABCB6(Full)	Incomplete	Uncharacterized	
	GgABCB20	TMD-NBD(Half)	AtABCB11(Full)	Incomplete	Uncharacterized	
	GgABCB21	NBD(Quarter)	AtABCB21(Full)	Incomplete	Auxin transport	72
	GgABCB22	TMD-NBD(Half)	AtABCB26(Half)	Complete	Uncharacterized	
	GgABCB23	TMD-NBD(Half)	AtABCB26(Half)	Complete	Uncharacterized	
	GgABCB24	NBD(Quarter)	AtABCB11(Full)	Incomplete	Uncharacterized	
	GgABCB25	TMD-NBD(Half)	AtABCB19(Full)	Incomplete	Auxin transport	34, 35
	GgABCB26	NBD-TMD-NBD(Half)	AtABCB19(Full)	Incomplete	Auxin transport	34, 35
	GgABCB27	*TMD-NBD(Half)	AtABCB19(Full)	Incomplete	Auxin transport	34, 35
	GgABCB28	TMD-NBD- TMD-NBD(Full)	AtABCB15(Full)	Complete	Auxin transport	34, 35
	GgABCB29	TMD-NBD(Half)	AtABCB15(Full)	Incomplete	Auxin transport	34, 35
	GgABCB30	TMD-NBD- TMD-NBD(Full)	AtABCB6(Full)	Complete	Uncharacterized	
	GgABCB31	NBD(Quarter)	AtABCB21(Full)	Incomplete	Auxin transport	72
	GgABCB32	TMD-NBD(Half)	AtABCB20(Full)	Incomplete	Uncharacterized	
	GgABCB33	TMD-NBD- TMD-NBD(Full)	AtABCB20(Full)	Complete	Uncharacterized	
	GgABCB34	*TMD-NBD-TMD-NBD(Full)	AtABCB20(Full)	Complete	Uncharacterized	
	GgABCB35	NBD(Quarter)	AtABCB28(Half)	Incomplete	Uncharacterized	
	GgABCB36	NBD(Quarter)	AtABCB28(Half)	Incomplete	Uncharacterized	
	GgABCB37	NBD(Quarter)	AtABCB15(Full)	Incomplete	Auxin transport	34, 35
	GgABCB38	NBD-TMD(Half)	AtABCB6(Full)	Incomplete	Uncharacterized	
	GgABCB39	TMD-NBD(Half)	AtABCB2(Full)	Incomplete	Uncharacterized	
	GgABCB40	*NBD-TMD-NBD(Half)	AtABCB19(Full)	Incomplete	Auxin transport	34, 35
	GgABCB41	*NBD –TMD(Half)	AtABCB19(Full)	Incomplete	Auxin transport	34, 35
	GgABCB42	*NBD-TMD-NBD(Half)	AtABCB1(Full)	Incomplete	Auxin transport	34, 35
	GgABCB43	*NBD-TMD-NBD(Half)	AtABCB13(Full)	Incomplete	Uncharacterized	
	GgABCB44	NBD-TMD(Half)	AtABCB11(Full)	Incomplete	Uncharacterized	
	GgABCB45	*NBD-TMD-NBD(Half)	AtABCB15(Full)	Incomplete	Auxin transport	34, 35
C (54)	GgABCC1	TMD-NBD-TMD-NBD*(Full)	AtABCC8(Full)	Complete	Uncharacterized	
	GgABCC2	TMD-NBD-TMD-NBD(Full)	AtABCC8(Full)	Complete	Uncharacterized	
	GgABCC3	TMD-NBD-TMD*(Half)	AtABCC8(Full)	Incomplete	Uncharacterized	
	GgABCC4	TMD-NBD-TMD-NBD(Full)	AtABCC4(Full)	Complete	Transport antifolate	84
	GgABCC5	TMD-NBD-TMD*(Half)	AtABCC4(Full)	Incomplete	Transport antifolate	84
	GgABCC6	TMD-NBD-TMD-NBD(Full)	AtABCC4(Full)	Complete	Transport antifolate	84
	GgABCC7	TMD-NBD(Half)	AtABCC14(Full)	Incomplete	Uncharacterized	
	GgABCC8	TMD-NBD-TMD-NBD(Full)	AtABCC8(Full)	Complete	Uncharacterized	
	GgABCC9	TMD-NBD(Half)	AtABCC10(Full)	Incomplete	Uncharacterized	
	GgABCC10	TMD-NBD-TMD-NBD(Full)	AtABCC10(Full)	Complete	Uncharacterized	
	GgABCC11	TMD-NBD-TMD-NBD(Full)	AtABCC10(Full)	Complete	Uncharacterized	
	GgABCC12	NBD(Quarter)	AtABCC10(Full)	Incomplete	Uncharacterized	
	GgABCC13	TMD-NBD(Half)	AtABCC9(Full)	Incomplete	Uncharacterized	
	GgABCC14	TMD-NBD(Half)	AtABCC9(Full)	Incomplete	Uncharacterized	
	GgABCC15	TMD-NBD-TMD-NBD(Full)	AtABCC9(Full)	Complete	Uncharacterized	
	GgABCC16	NBD(Quarter)	AtABCC9(Full)	Incomplete	Uncharacterized	
	GgABCC17	NBD(Quarter)	AtABCC9(Full)	Incomplete	Uncharacterized	
	GgABCC18	TMD-NBD-TMD*(Half)	AtABCC14(Full)	Incomplete	Uncharacterized	
	GgABCC19	TMD-NBD-TMD-NBD(Full)	AtABCC4(Full)	Complete	Transport antifolate	84
	GgABCC20	TMD-NBD(Half)	AtABCC14(Full)	Incomplete	Uncharacterized	
	GgABCC21	NBD(Quarter)	AtABCC4(Full)	Incomplete	Transport antifolate	84
	GgABCC22	TMD-NBD(Half)	AtABCC10(Full)	Incomplete	Uncharacterized	
	GgABCC23	TMD-NBD(Half)	AtABCC4(Full)	Incomplete	Uncharacterized	
	GgABCC24	NBD(Quarter)	AtABCC5(Full)	Incomplete	Inositol hexakisphosphate transport and Stomatal regulation	40, 41
	GgABCC25	TMD-NBD(Half)	AtABCC5(Full)	Incomplete	Inositol hexakisphosphate transport and Stomatal regulation	40, 41
	GgABCC26	*NBD-TMD-NBD(Half)	AtABCC5(Full)	Incomplete	Inositol hexakisphosphate transport and Stomatal regulation	40, 41
	GgABCC27	TMD-NBD-TMD*(Half)	AtABCC14(Full)	Incomplete	Uncharacterized	
	GgABCC28	TMD-NBD-TMD-NBD*(Full)	AtABCC10(Full)	Complete	Uncharacterized	
	GgABCC29	TMD-NBD(Half)	AtABCC14(Full)	Incomplete	Uncharacterized	
	GgABCC30	TMD-NBD-TMD(Half)	AtABCC3(Full)	Incomplete	Cd and As tolerance	85
	GgABCC31	TMD-NBD-TMD*(Half)	AtABCC3(Full)	Incomplete	Cd and As tolerance	85
	GgABCC32	TMD-NBD(Half)	AtABCC13(Full)	Incomplete	Uncharacterized	
	GgABCC33	*TMD-NBD(Half)	AtABCC13(Full)	Incomplete	Uncharacterized	
	GgABCC34	NBD(Quarter)	AtABCC3(Full)	Incomplete	Cd and As tolerance	85

	GgABCC35	TMD-NBD(Half)	AtABCC3(Full)	Incomplete	Cd and As tolerance	85
	GgABCC36	TMD-NBD(Half)	AtABCC3(Full)	Incomplete	Cd and As tolerance	85
	GgABCC37	NBD(Quarter)	AtABCC3(Full)	Incomplete	Cd and As tolerance	85
	GgABCC38	NBD(Quarter)	AtABCC3(Full)	Incomplete	Cd and As tolerance	85
	GgABCC39	*NBD-TMD-NBD(Half)	AtABCC2(Full)	Incomplete	Cd and Hg tolerance, transport Glutathione S-conjugates and chlorophyll catabolites.	86, 87
	GgABCC40	TMD-NBD(Half)	AtABCC2(Full)	Incomplete	Cd and Hg tolerance, transport Glutathione S-conjugates and chlorophyll catabolites.	86, 87
	GgABCC41	TMD-NBD-TMD-NBD(Full)	AtABCC3(Full)	Complete	Cd and As tolerance	85
	GgABCC42	TMD-NBD(Half)	AtABCC3(Full)	Incomplete	Cd and As tolerance	85
	GgABCC43	TMD-NBD(Half)	AtABCC7(Full)	Incomplete	Uncharacterized	
	GgABCC44	TMD-NBD*(Half)	AtABCC2(Full)	Incomplete	Cd and Hg tolerance, transport Glutathione S-conjugates and chlorophyll catabolites	86, 87
	GgABCC45	TMD-NBD*(Half)	AtABCC2(Full)	Incomplete	Cd and Hg tolerance, transport Glutathione S-conjugates and chlorophyll catabolites	86, 87
	GgABCC46	NBD(Quarter)	AtABCC4(Full)	Incomplete	Transport antifolate	84
	GgABCC47	NBD(Quarter)	AtABCC14(Full)	Incomplete	Uncharacterized	
	GgABCC48	TMD-NBD*(Half)	AtABCC10(Full)	Incomplete	Uncharacterized	
	GgABCC49	TMD-NBD-TMD*(Half)	AtABCC3(Full)	Incomplete	Cd and As tolerance	85
	GgABCC50	TMD-NBD-TMD-NBD(Full)	AtABCC6(Full)	Complete	Cd tolerance	88
	GgABCC51	TMD-NBD-TMD*(Half)	AtABCC5(Full)	Incomplete	Inositol hexakisphosphate transport and Stomatal regulation.	40, 41
	GgABCC52	TMD-NBD-TMD-NBD*(Full)	AtABCC3(Full)	Complete	Cd and As tolerance	85
	GgABCC53	TMD-NBD-TMD-NBD*(Full)	AtABCC3(Full)	Complete	Cd and As tolerance	85
	GgABCC54	TMD-NBD*(Half)	AtABCC12(Full)	Incomplete	Uncharacterized	
F (13)	GgABCF1	Xtn-NBD(Quarter)	AtABCF1(Soluble)	Incomplete	Translational regulation in response to pathogen infection.	45
	GgABCF2	NBD- Xtn-NBD(Soluble)	AtABCF1(Soluble)	Complete	Translational regulation in response to pathogen infection	45
	GgABCF3	Xtn-NBD(Quarter)	AtABCF3(Soluble)	Incomplete	Translational regulation in response to pathogen infection, root growth and hydrogen peroxide (H_2O_2) transport	45, 46, 47
	GgABCF4	NBD(Quarter)	AtABCF3(Soluble)	Incomplete	Translational regulation in response to pathogen infection, root growth and hydrogen peroxide (H_2O_2) transport	45, 46, 47
	GgABCF5	NBD(Quarter)	AtABCF3(Soluble)	Incomplete	Translational regulation in response to pathogen infection, root growth and hydrogen peroxide (H_2O_2) transport.	45, 46, 47
	GgABCF6	NBD(Quarter)	AtABCF3(Soluble)	Incomplete	Translational regulation in response to pathogen infection, root growth and hydrogen peroxide (H_2O_2) transport.	45, 46, 47
	GgABCF7	$(NBD)_2$(Soluble)	AtABCF4(Soluble)	Complete	Protein degradation.	48
	GgABCF8	NBD- Xtn-NBD(Soluble)	AtABCF5(Soluble)	Complete	Uncharacterized	
	GgABCF9	NBD- Xtn-NBD(Soluble)	AtABCF5(Soluble)	Complete	Uncharacterized	
	GgABCF10	NBD- Xtn-NBD(Soluble)	AtABCF5(Soluble)	Complete	Uncharacterized	
	GgABCF11	NBD- Xtn-NBD(Soluble)	AtABCF5(Soluble)	Complete	Uncharacterized	
	GgABCF12	NBD-Xtn(Quarter)	AtABCF5(Soluble)	Incomplete	Uncharacterized	
	GgABCF13	NBD(Quarter)	AtABCF5(Soluble)	Incomplete	Uncharacterized	
	GgABCG1	NBD-TMD(Half)	AtABCG20(Half)	Complete	Seed dormancy, stomatal regulation and suberin barrier formation.	55, 56, 57
	GgABCG2	NBD-TMD(Half)	AtABCG20(Half)	Complete	Seed dormancy, Stomatal regulation and Suberin barrier formation.	55, 56, 57
	GgABCG3	NBD-TMD(Half)	AtABCG22(Half)	Complete	Stomatal regulation, ABA signaling and lignifications	56
	GgABCG4	NBD-TMD(Half)	AtABCG27(Half)	Complete	Cellulose synthesis	89
	GgABCG5	TMD-PDR-NBD-TMD(Half)	AtABCG39(Full)	Incomplete	Herbicide resistance	90
	GgABCG6	TMD-PDR-NBD-TMD(Half)	AtABCG39(Full)	Incomplete	Herbicide resistance	90
	GgABCG7	NBD-TMD(Half)	AtABCG39(Full)	Incomplete	Herbicide resistance	90
	GgABCG8	NBD-TMD-PDR-NBD-TMD(Full)	AtABCG36(Full)	Complete	Cd, Pb resistance, Pathogen defense, drought resistance and auxin precursor transport.	91, 92, 93, 94

G (50)	GgABCG9	NBD-TMD(Half)	AtABCG14(Half)	Complete	Lipid/sterol homoeostasis, vascular system development, cytokinin translocation, plant growth and immunity	51, 52, 53, 54
	GgABCG10	NBD-TMD(Half)	AtABCG14(Half)	Complete	Lipid/sterol homoeostasis, vascular system development, cytokinin translocation, plant growth and immunity	51, 52, 53, 54
	GgABCG11	NBD(Quarter)	AtABCG9(Half)	Incomplete	Lipid/sterol homoeostasis, vascular system development, and pollen coat formation.	51, 95, 96
	GgABCG12	NBD-TMD(Half)	AtABCG25(Half)	Complete	Stomatal regulation, ABA signaling and ABA secretion from endosperm.	97, 98, 99
	GgABCG13	NBD-TMD(Half)	AtABCG25(Half)	Complete	Stomatal regulation, ABA signaling and ABA secretion from endosperm	97, 98, 99
	GgABCG14	TMD-PDR-NBD-TMD(Half)	AtABCG31(Full)	Incomplete	Transport sterols and ABA uptake	98
	GgABCG15	NBD-TMD(Half)	AtABCG31(Full)	Incomplete	Transport sterols and ABA uptake	98
	GgABCG16	TMD-PDR-NBD-TMD*(Half)	AtABCG31(Full)	Incomplete	Transport sterols and ABA uptake	98
	GgABCG17	NBD-TMD(Half)	AtABCG21(Half)	Complete	Stomatal regulation	56
	GgABCG18	NBD-TMD(Half)	AtABCG21(Half)	Complete	Stomatal regulation	56
	GgABCG19	NBD(Quarter)	AtABCG15(Half)	Incomplete	Uncharacterized	
	GgABCG20	NBD(Quarter)	AtABCG40(Full)	Incomplete	ABA uptake, Pathogen defense and detoxification.	6, 100, 101
	GgABCG21	NBD-TMD(Half)	AtABCG22(Half)	Complete	Stomatal regulation, ABA Signaling and lignifications.	56
	GgABCG22	NBD-TMD(Half)	AtABCG11(Half)	Complete	Transport suberin precursors	102
	GgABCG23	NBD-TMD(Half)	AtABCG5(Half)	Complete	Secretion of Cutin and wax precursors	107
	GgABCG24	NBD-TMD(Half)	AtABCG11(Half)	Complete	Transport suberin precursors	102
	GgABCG25	NBD(Quarter)	AtABCG11(Half)	Incomplete	Transport suberin precursors	102
	GgABCG26	NBD(Quarter)	AtABCG11(Half)	Incomplete	Transport suberin precursors	102
	GgABCG27	NBD(Quarter)	AtABCG11(Half)	Incomplete	Transport suberin precursors	102
	GgABCG28	NBD-TMD(Half)	AtABCG10(Half)	Complete	Uncharacterized	
	GgABCG29	TMD-PDR-NBD-TMD(Half)	AtABCG32(Full)	Incomplete	Secretion of cutin and wax precursors	103
	GgABCG30	NBD-TMD(Half)	AtABCG11(Half)	Complete	Transport suberin precursors	102
	GgABCG31	NBD(Quarter)	AtABCG11(Half)	Incomplete	Transport suberin precursors	102
	GgABCG32	NBD-TMD(Half)	AtABCG11(Half)	Complete	Transport suberin precursors	102
	GgABCG33	NBD(Quarter)	AtABCG7(Half)	Incomplete	Uncharacterized	
	GgABCG34	NBD-TMD(Half)	AtABCG23(Half)	Complete	Uncharacterized	
	GgABCG35	NBD-TMD(Half)	AtABCG15(Half)	Complete	Uncharacterized	
	GgABCG36	NBD-TMD(Half)	AtABCG11(Half)	Complete	Transport suberin precursors	102
	GgABCG37	NBD-TMD(Half)	AtABCG8(Half)	Complete	Uncharacterized	
	GgABCG38	NBD-TMD(Half)	AtABCG15(Half)	Complete	Uncharacterized	
	GgABCG39	NBD(Quarter)	AtABCG3(Half)	Incomplete	Uncharacterized	
	GgABCG40	NBD(Quarter)	AtABCG3(Half)	Incomplete	Uncharacterized	
	GgABCG41	*NBD-TMD(Half)	AtABCG11(Half)	Complete	Transport suberin precursors	102
	GgABCG42	NBD(Quarter)	AtABCG3(Half)	Incomplete	Uncharacterized	
	GgABCG43	NBD(Quarter)	AtABCG28(Half)	Incomplete	Sequestering polyamines	104
	GgABCG44	NBD(Quarter)	AtABCG28(Half)	Incomplete	Sequestering polyamines	104
	GgABCG45	TMD-PDR-NBD(Half)	AtABCG39(Full)	Incomplete	Herbicide resistance	90
	GgABCG46	NBD-TMD(Half)	AtABCG9(Half)	Complete	Lipid/ sterol homoeostasis, vascular system development, and pollen coat formation.	51, 95, 96
	GgABCG47	NBD-TMD(Half)	AtABCG41(Full)	Incomplete	Uncharacterized	
	GgABCG48	NBD-TMD*(Half)	AtABCG29(Full)	Incomplete	Transport Monolignol	105
	GgABCG49	NBD-TMD(Half)	AtABCG40(Full)	Incomplete	ABA uptake, Pathogen defense and detoxification	6, 100, 101
	GgABCG50	NBD(Quarter)	AtABCG34(Full)	Incomplete	Camalexin secretion	106
I (8)	GgABCI1	NBD(Quarter)	AtABCI6(Quarter)	Complete	Biosynthesis of Fe-S cluster	58
	GgABCI2	NBD(Quarter)	AtABCI6(Quarter)	Complete	Biosynthesis of Fe-S cluster	58
	GgABCI3	NBD(Quarter)	AtABCI10(Quarter)	Complete	Chloroplast metal transportation	62
	GgABCI4	NBD(Quarter)	AtABCI10(Quarter)	Complete	Chloroplast metal transportation	62
	GgABCI5	NBD(Quarter)	AtABCI17(Quarter)	Complete	Al tolerance	61
	GgABCI6	NBD(Quarter)	AtABCI17(Quarter)	Complete	Al tolerance	61
	GgABCI7	NBD(Quarter)	AtABCI1(Quarter)	Complete	Cytochrome c maturation	59
	GgABCI8	NBD(Quarter)	AtABCI13(Quarter)	Complete	Lipid formation	60

Supplementary Table S4: Co-expression score between the interactive partners.

S.No.	Node1	Node2	Co-expression Score
1	ABCB1	AUX1	0.059
2	ABCB1	PIN3	0.097
3	ABCB19	AUX1	0.205
4	ABCB19	PIN1	0.062
5	ABCB19	PIN3	0.162
6	ABCB4	EIR1	0.129
7	AUX1	ABCB1	0.059
8	AUX1	ABCB19	0.205
9	AUX1	PIN3	0.213
10	EIR1	ABCB4	0.129
11	PIN1	ABCB19	0.062
12	PIN3	ABCB1	0.097
13	PIN3	ABCB19	0.162
14	PIN3	AUX1	0.213

Supplementary Table S5: Component localization of interactive partners in protein-protein interaction network.

S.No.	Interactive Partners in PPI	Localization
1	PIN1	apical part of cell, basal plasma membrane and cytoplasm
2	PIN3	lateral plasma membrane, vesicle membrane and cell surface
3	ABCB19	cytosol, integral component of membrane, nucleus and plasma membrane
4	ABCB1	endoplasmic reticulum, integral component of membrane, nucleus, plasma membrane and plasmodesma
5	ABCB4	cytosol, integral component of membrane, nucleus, plasma membrane and plasmodesma
6	AUX1	golgi apparatus, plasma membrane, cell surface and endosome
7	EIR1	basal plasma membrane, chloroplast, lytic vacuole and plasma membrane
8	TWD1	plasma membrane
9	ABCB11	extracellular region, integral component of membrane, nucleus, plasma membrane and plasmodesma
10	ABCB21	cytosol, integral component of membrane, nucleus, plasma membrane and vacuolar membrane

Supplementary Table S6: The quality control summary of RNA isolated for library preparation.

Sample Name	Nano Drop Concentration (ng/µl)	Qubit Concentration (ng/µl)	A260/280	RIN value
Gly Shoot 1	2505.5	>1000	2.20	5.3
Gly Shoot 2	897.2	>1000	2.14	5.6
Gly Shoot 3	1188.4	>1000	2.33	5.5
Gly Shoot 4	968.1	>1000	2.14	4.9
Gly Shoot 5	2257.1	>1000	2.22	5.7
Gly Root 1	781	670	2.10	5.6
Gly Root 2	546	412	2.0	5.6
Gly Root 3	394	406	2.11	5.6
Gly Root 4	408	392	2.10	5.8
Gly Root 5	604	552	2.10	5.0

RIN Values: >7.5(Good for RNA Seq library prep), >6- 7.5(Optimal for RNA Seq library prep), <6(Poor for RNA Seq library prep)
